# Cytotoxic Polyketides from the Deep-Sea-Derived Fungus *Engyodontium album* DFFSCS021

**DOI:** 10.3390/md12125902

**Published:** 2014-12-09

**Authors:** Qifeng Yao, Jie Wang, Xiaoyong Zhang, Xuhua Nong, Xinya Xu, Shuhua Qi

**Affiliations:** 1 Key Laboratory of Tropical Marine Bio-resources and Ecology, Guangdong Key Laboratory of Marine Materia Medica, RNAM Center for Marine Microbiology, South China Sea Institute of Oceanology, Chinese Academy of Sciences, 164 West Xingang Road, Guangzhou 510301, Guangdong, China; E-Mails: yqf20081@163.com (Q.Y.); wangjielangjing@126.com (J.W.); zhangxiaoyong@scsio.ac.cn (X.Z.); nongxuhua4883@163.com (X.N.); xuxinya@scsio.ac.cn (X.X.); 2 University of Chinese Academy of Sciences, Beijing 100049, China

**Keywords:** *Engyodontium album*, polyketides, spectroscopic data, cytotoxicity, antilarval

## Abstract

Eight new chromones, engyodontiumones A–H (**1**–**8**), and three new phenol derivatives (**9**–**11**) together with eight known polyketides (**12**–**19**) were isolated from the deep-sea-derived fungus *Engyodontium album* DFFSCS021. Their structures were identified by extensive spectroscopic analysis. Compounds **8** and **16** showed significant selective cytotoxicity against human histiocytic lymphoma U937 cell line with IC_50_ values of 4.9 and 8.8 μM, respectively. In addition, this is the first time to report that **8**, **15** and **16** had mild antibacterial activity against *Escherichia coli* and *Bacillus subtilis*, and **15** showed potent antilarval activity against barnacle *Balanus amphitrite* larval settlement.

## 1. Introduction

**Figure 1:**
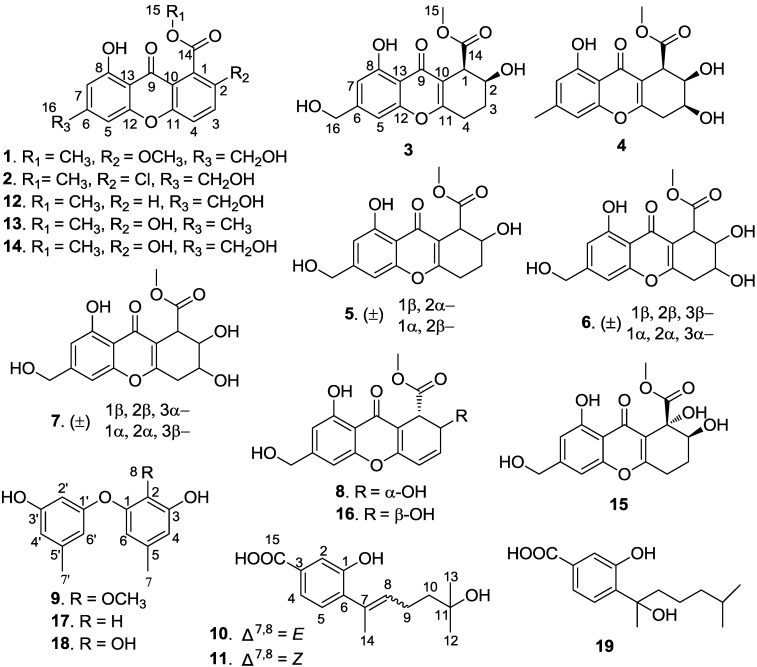
Structures of compounds **1**–**19**.

Recently, with the development of methods for sample collection and culturing technologies, an increasing number of deep-sea-derived fungi have been reported to produce novel bioactive secondary metabolites [1,2]. In our ongoing search for structurally unique and biologically active compounds from deep-sea-derived microorganisms [3], a fungal strain, identified as *Engyodontium album*, isolated from a marine sediment sample collected in the South China Sea (19°00′368″N, 117°58′223″E, 3739 m depth), attracted our attention. Preliminary experiments demonstrated that the extract of the culture medium of the strain showed toxicity towards brine shrimp and antibacterial activity. Further investigation on the chemical constituents of the extract resulted in the isolation of eight new chromones (**1**–**8**) and three new phenol derivatives (**9**–**11**) together with eight known compounds: sydowinin A (**12**) [4], pinselin (**13**) [5], sydowinin B (**14**) [5,6], aspergillusone B (**15**) [7], AGI-B4 (**16**) [8], diorcinol (**17**) [9], cordyol C (**18**) [10], and hydroxysydonic acid (**19**) [11] (Figure 1). The cytotoxicity, antibacterial, and antilarval activities of these compounds were individually evaluated. This paper describes the isolation, structure elucidation, and bioactivity of these compounds.

## 2. Results and Discussion

Engyodontiumone A (**1**) had a molecular formula of C_17_H_14_O_7_ as established by HRESIMS (*m/z* 331.0801 [M + H]^+^). Its NMR data (Table 1 and Table 2) was similar to that of sydowinin B [5,6] with the only difference of one methoxyl (δ_C_ 56.8) and the absence of a hydroxyl proton signal in **1**, which indicated that **1** was a chromone skeleton. In the HMBC spectrum (see Supporting Information Figure S3), correlations of δ_H_ 12.11 with C-7/C-8/C-13 suggested a hydroxyl group located at C-8, correlations of δ_H_ 3.88 (3H, s) with C-2 suggested a methoxy group located at C-2, and correlations of δ_H_ 4.60 (2H, s) with C-5/C-6/C-7 suggested a hydroxymethene group attached at C-6. In addition, HMBC correlations of δ_H_ 3.87 (3H, s) with C-14 combined with the chemical shift of C-1 (δ_C_ 120.3, C), suggested a methoxycarbonyl group located at C-1. Therefore, the structure of **1** was determined as shown.

Engyodontiumone B (**2**) had a molecular formula of C_16_H_11_ClO_6_ as inferred from its HRESIMS (*m/z* 335.0309 [M + H]^+^) showing peaks at *m/z* 335.0309 and 337.0264 with the height ratio of 3:1. Its NMR data (Table 1 and Table 2) showed great similarity to those of sydowinin B [5,6] with the only obvious difference of the high-field shift of the chemical shift of C-2 (from δ_C_ 148.9 in sydowinin B to δ_C_ 125.5 in **2**) suggesting the loss of the hydroxyl functionality. Combined with the molecular formula and HMBC spectral data (see Supporting Information Figure S8), these data suggested a chlorine atom attached at C-2. Therefore, the structure of **2** was determined as shown.

**Figure 2:**
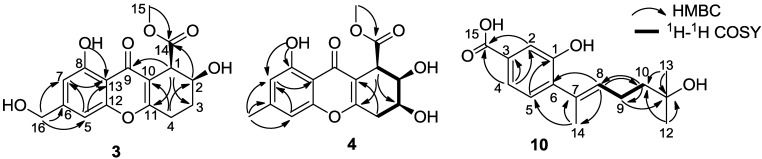
Key HMBC and ^1^H–^1^H COSY correlations of compounds **3**, **4** and **10**.

The molecular formula C_16_H_16_O_7_ of engyodontiumone C (**3**) was deduced from its HRESIMS (*m/z* 321.0972 [M + H]^+^). Its NMR data (Table 1 and Table 2) showed great similarity to those of aspergillusone B^7^ with the only difference is the presence of one extra methine (δ_H_ 3.84 and δ_C_ 43.5) and the absence of one oxygenated quaternary carbon (δ_C_ 74.3 (C-1) in aspergillusone B), which suggested that **3** had the same skeleton as aspergillusone B with the only difference being the substitution at C-1. This was proved by the HMBC spectrum (Figure 2) showing correlation of H-1 (δ_H_ 3.84) with C-3/C-9/C-11. The coupling constant values of *J*_H-1,H-2_ (6.0 Hz) and *J*_H-2,H-3_ (3.5, 9.5 Hz) indicated that H-1 and H-2 were equatorial–axial relationship and required the placement of H-1 and H-2 in pseudo equatorial and axial orientations [12], respectively (Table 3). We failed to determine the absolute structure of **3** by Mosher ester methods, because the OH- at C-16 was easier to be esterified than OH- at C-2. In the experiment, the main product of the Mosher ester reaction of **3** was the C-16 esterification, and the trace product was the ester esterified at C-2, C-8, and C-16.

**Table 1:** ^1^H NMR Data for **1**–**8** (500 MHz, in DMSO-*d*_6_, δ ppm, *J* in Hz).

Position	1	2	3	4	5	6	7	8
	δ_H_ (*J*)	δ_H_ (*J*)	δ_H_ (*J*)	δ_H_ (*J*)	δ_H_ (*J*)	δ_H_ (*J*)	δ_H_ (*J*)	δ_H_ (*J*)
1			3.84, d (6.0)	3.78, d (5.5)	3.62, d (3.5)	3.60, d (4.5)	3.63, d (6.0)	4.03, d (10.0)
2			4.11, ddd (3.5, 6.0, 9.5)	3.99, br t (5.5)	4.14, ddd (2.5, 3.5, 6.0)	4.15, br t (4.5)	3.99, dd (1.5, 6.0)	5.01, br d (10.0)
3	7.79, d (9.0)	8.06, d (9.0)	1.84, m 2.01, m	4.04, ddd (4.5, 5.0, 5.5)	1.80, m 1.90, m	3.96, ddd (3.5, 4.5, 4.5)	3.92, ddd (1.5, 4.5, 6.5)	6.49, d (2.0)
4	7.82, d (8.5)	7.84, d (9.0)	2.73–2.89, m	3.10, dd (4.0, 18.5)	2.73–2.87, m	3.03, dd (4.5, 18.5)	3.07, dd (4.5, 18.5)	6.36, d (2.0)
				2.60, dd (5.0, 18.5)		2.70, dd (3.5, 18.5)	2.79, dd (6.5, 18.5)	
5	7.02, s	7.05, s	6.96, s	6.89, s	6.98, s	6.98, s	6.98, s	6.98, s
7	6.77, s	6.81, s	6.73, s	6.65, s	6.74, s	6.74, s	6.74, s	6.75, s
15	3.87, s	3.95, s	3.60, s	3.60, s	3.64, s	3.65, s	3.65, s	3.58, s
16	4.60, s	4.62, d (4.5)	4.56, s	2.37, s	4.58, s	4.56, s	4.56, s	4.56, s
2-OH					5.49, brs			5.90, brs
8-OH	12.11, s	11.88, brs	12.29, s		12.29, s	12.41, brs	12.25, brs	12.39, s
16-OH		5.58, t (4.5)				5.50, brs		5.50, brs
8-OCH_3_	3.88, s							

**Table 2:** ^13^C NMR Data of **1**–**8** (125 MHz, in DMSO-*d*_6,_ δ ppm).

Position	1	2	3	4	5	6	7	8
	δ_C_	δ_C_	δ_C_	δ_C_	δ_C_	δ_C_	δ_C_	δ_C_
1	120.3, C	131.0, C	43.5, CH	42.3, CH	46.2, CH	43.5, CH	44.6, CH	41.7, CH
2	154.4, C	125.5, C	65.1, CH	68.1, CH	65.4, CH	66.3, CH	65.8, CH	65.9, CH
3	121.3, CH	136.4, CH	26.1, CH_2_	66.1, CH	26.1,CH_2_	69.8, CH	69.9, CH	145.0, CH
4	120.8, CH	121.3, CH	25.7, CH_2_	32.8, CH_2_	23.8, CH_2_	32.9, CH_2_	33.4, CH_2_	119.7, CH
5	103.9, CH	104.1, CH	103.8, CH	107.2, CH	103.9, CH	103.9, CH	103.9, CH	104.1, CH
6	155.5, C	154.8, C	155.5, C	147.2, C	152.3, C	152.1, C	152.3, C	152.2, C
7	107.2, CH	107.8, CH	107.7, CH	111.4, CH	107.8, CH	107.9, CH	107.9, CH	108.3, C
8	160.2, C	160.4, C	159.3, C	159.2, C	159.3, C	159.4, C	159.3, C	159.3, C
9	180.2, C	179.2, C	181.1, C	180.7, C	181.2, C	180.1, C	180.8, C	180.0, C
10	117.1, C	118.0, C	113.9, C	113.1, C	113.1, C	112.7, C	113.0, C	111.0, C
11	152.0, C	154.3, C	166.9, C	164.1, C	166.7, C	164.3, C	164.8, C	160.4, C
12	156.2, C	155.3, C	152.2, C	155.6, C	155.5, C	155.7, C	155.7, C	154.9, C
13	107.0, C	106.9, C	107.9, C	107.2, C	107.9, C	107.8, C	107.9, C	108.3, C
14	166.2, C	165.7, C	171.4, C	170.0, C	172.0, C	170.7, C	172.3, C	169.7, C
15	52.4, CH_3_	52.9, CH_3_	51.5, CH_3_	51.5, CH_3_	52.0, CH_3_	51.5, CH_3_	51.9, CH_3_	51.5, CH_3_
16	62.3, CH_2_	62.2, CH_2_	62.1, CH_2_	21.7, CH_3_	62.2, CH_2_	62.2, CH_2_	62.1, CH_2_	62.1, CH_2_
2-OCH_3_	58.6, OCH_3_							

**Table 3:** ^1^H NMR (DMSO-*d*_6_) *J* values (Hz) for compounds **3**–**7**. 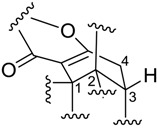

Compound	*J*_H-1,H-2_	*J*_H-2,H-3_	*J*_H-3,H-4_	Orientations of H-1, H-2, H-3
**3**	6.0	3.5, 9.5		H-1: equatorial, H-2: axial
**4**	5.5	5.5	4.5, 5.0	H-1: equatorial, H-2: axial, H-3: equatorial
**5**	3.5	2.5, 6.0		H-1: equatorial, H-2: equatorial
**6**	4.5	4.5	3.5, 4.5	H-1: equatorial, H-2: axial, H-3: equatorial
**7**	6.0	1.5	4.5, 6.5	H-1: axial, H-2: equatorial, H-3: equatorial

The molecular formula C_16_H_16_O_8_ of engyodontiumone D (**4**) was inferred from its HRESIMS (*m/z* 321.0974 [M + H]^+^). Its NMR data (Table 1 and Table 2) were very similar to those of **3**, except for the presence of one methyl group (δ_H_ 2.37, δ_C_ 21.7) and one oxygenated methine (δ_H_ 4.06, δ_C_ 66.1) and absence of two methylenes. The HMBC spectrum (Figure 2) showing correlations of H-1 with C-2/C-10/δ_C_ 66.1, H-2 with C-1/δ_C_ 66.1, and ^1^H–^1^H COSY spectrum (Figure 2) showing correlations of H-2 with H-1/δ_H_ 4.06, suggested the oxygenation of C-3 (δ_H_ 4.06, δ_C_ 66.1). In addition, correlations of δ_H_ 2.37 with C-5/C-6/C-7 suggested that a methyl group CH_3_-16 was attached at C-6. The coupling constant values of *J*_H-1,H-2_ (5.5 Hz), *J*_H-2,H-3_ (5.5 Hz) and *J*_H-3,H-4_ (4.5, 5.0 Hz) indicated the equatorial–axial relationship for H-1/H-2 and H-2/H-3 placing H-1, H-2 and H-3 in pseudo equatorial, axial and equatorial orientations [12], respectively (Table 3). Therefore, the structure of **4** was determined as shown. 

Engyodontiumone E (**5**) had the same molecular formula of C_16_H_16_O_7_ as **3**, which was deduced from its HRESIMS (*m/z* 321.0968 [M + H]^+^). Its NMR data (Table 1 and Table 2) were very similar to those of **3** except for the obvious changes of the chemical shifts of H-1 and C-1. Further detailed analysis of HSQC and HMBC spectra (see Supporting Information Figure S27) suggested that **3** and **5** had the same planar structure, and **5** that was an isomer of **3**. The coupling constant values of *J*_H-1,H-2_ (3.5 Hz) and *J*_H-2,H-3_ (2.5, 6.0 Hz) indicated the equatorial–equatorial relationship for H-1 and H-2 placing of H-1 and H-2 in pseudo equatorial orientation (Table 3). Compound **5** was inferred to be racemic by its zero value of specific optical rotation and HPLC analysis using a chiral column (see Supporting Information Figure S68).

Engyodontiumone F (**6**) had a molecular formula of C_16_H_16_O_8_ as deduced from its HRESIMS (*m/z* 337.0922 [M + H]^+^). Its NMR data (Table 1 and Table 2) showed great similarity to those of **4** with the only difference of one methyl group (CH_3_-16) oxygenated to be methylene (δ_H_ 5.50, δ_C_ 62.2). This was supported by the HMBC spectrum (see Supporting Information Figure S34) showing correlation of H-16 (δ_H_ 5.50) with C-5/C-6/C-7. The coupling constant values of *J*_H-1, H-2_ (4.5 Hz), *J*_H-2,H-3_ (4.5 Hz) and *J*_H-3,H-4_ (3.5, 4.5 Hz) indicated the axial–equatorial relationship for H-1/H-2 and H-2/H-3 placing H-1, H-2 and H-3 in pseudo equatorial, axial and equatorial orientations, respectively (Table 3). The relative configuration of H-2 and H-3 was supported by the NOESY spectrum showing correlations of OH-2/OH-3 with OH-8. Compound **6** was also inferred to be racemic by its zero value of specific optical rotation and HPLC analysis using a chiral column (see Supporting Information Figure S69).

Engyodontiumone G (**7**) had the same molecular formula of C_16_H_16_O_8_ as **6**, which was deduced from its HRESIMS (*m/z* 337.0922 [M + H]^+^). Its NMR data (Table 1 and Table 2) was similar to those of **6**. Further detailed analysis of HSQC and HMBC spectra (see Supporting Information Figures S41 and S42) suggested that **7** and **6** shared the same planar structure suggesting that **7** is an isomer of **6**. The coupling constant values of *J*_H-1,H-2_ (4.5 Hz), *J*_H-2,H-3_ (1.5 Hz), and *J*_H-3,H-4_ (4.5, 6.5 Hz) indicated that axial–equatorial relationship for H-1 and H-2 and equatorial–equatorial relationship for H-2 and H-3 placing H-1, H-2 and H-3 in pseudo axial, equatorial and equatorial orientations, respectively (Table 3). Compound **7** was also inferred to be racemic by its zero value of specific optical rotation and HPLC analysis using a chiral column (see Supporting Information Figure S70).

Engyodontiumone H (**8**) had the same molecular formula of C_16_H_14_O_7_ as AGI-B4 [8] as deduced from its HRESIMS (*m/z* 321.0496 [M + H]^+^). Its NMR data (Table 1 and Table 2) also showed great similarity to those of AGI-B4 [7] except for a change in the chemical shift of H-2 (from δ_H_ 4.60 in AGI-B4 to δ_H_ 5.01 in **8**) and the coupling constant value *J*_H-1,H-2_ (from 3.5 Hz in AGI-B4 to 10.0 Hz in **8**), suggesting **8** is an epimer of AGI-B4 at C-2. Further detailed analysis of HMBC spectrum (see Supporting Information Figure S48) proved that **8** and AGI-B4 shared the same planar structure, and the coupling constant value of *J*_H-1,H-2_ (10.0 Hz) indicated that H-1 and H-2 were in a *cis*-configuration. We also failed to determine the absolute structure of **8** by Mosher ester methods as compound **3**.

The molecular formula of **9** was established as C_15_H_16_O_4_ by its HRESIMS (*m/z* 261.1122 [M + H]^+^). Its NMR data showed close similarity to those of cordyol C [10] with the exception of one methoxyl group (δ_C_ 60.1, δ_H_ 3.60), which indicated that **9** had the same skeleton as cordyol C. The HMBC spectrum showed correlations of δ_H_ 3.60 (H-8) with C-2 (δ_C_137.3), suggesting the methoxyl group (δ_C_ 60.1, δ_H_ 3.60) attached at C-2. Therefore, the structure of **9** was determined as shown, and named 2-methoxyl cordyol C.

Engyodontiumone I (**10**) had a molecular formula of C_15_H_20_O_4_ as inferred from its HRESIMS data (*m/z* 265.1426 [M + H]^+^). Its ^1^H NMR spectrum exhibited characteristic signals for a trisubstituted benzene ring (δ_H_ 7.40 (1H, d, *J* = 1.5 Hz), 7.33 (1H, dd, *J* = 1.5, 7.5 Hz), 7.09 (1H, d, *J* = 7.5 Hz)) and a trisubstituted double-bond olefinic proton (δ_H_ 5.49 (1H, m)). The ^13^C NMR and DEPT spectral data revealed 15 carbon signals including six quaternary carbons, four methines, two methylenes, and three methyls. These data showed similarity to those of hydroxysydonic acid [11] with the obvious difference of the additional appearance of one trisubstituted double-bond (δ_C_ 129.9 (C), 130.8 (CH)) and disappearance of one methine and one methene. In the HMBC spectrum (Figure 2), correlations of H-8 with C-6/C-7/C-10/C-14 suggested one double bond between C-7 and C-8, and correlations of H-12/H-13 with δ_C_ 68.5 (C, C-11) suggested the oxygenation of C-11. The chemical shift of C-14 (δ_C_ 16.4) indicated that the double-bond between C-7 and C-8 was in an *E*-configuration [13]. Therefore, the structure of **10** was determined as shown.

Engyodontiumone J (**11**) had the same molecular formula C_15_H_20_O_4_ as **10**, which was deduced from its HRESIMS (*m/z* 265.1428 [M + H]^+^). Its NMR data were very similar to those of **10** except for the chemical shift changes of H-9, C-3 and C-14. Further detailed analysis of the HMBC spectrum suggested that **11** and **10** had the same planar structure. The chemical shift of C-14 (δ_C_ 23.8) indicated that the double bond between C-7 and C-8 was in a Z-configuration [13].

Antibacterial activities of compounds **1**–**19** were tested against *Escherichia coli* and *Bacillus subtilis*. Preliminary antibacterial assay results (Table 4) showed that at a concentration of 25 μg/disc compounds **8**, **10**, **15**, **16** and **19** inhibiting the growth of *E. coli* and *B. subtilis*, while the remaining compounds had no obvious antibacterial activity. The MIC values of **8**, **10**, **15**, **16** and **19** were further tested using micro-dilution method. The results (Table 4) showed that **8**, **15** and **16** had mild antibacterial activity against *E. coli* and *B. subtilis* with MIC ≤ 64 μg/mL.

Chromones (**2**–**8**, **12**–**14**, **16**) were also tested for cytotoxicity against human helacyton gartleri Hela, breast cancer MCF-7, liver hepatocellular carcinoma HepG2 and Huh7 cell lines (Table 4) by MTT method, and against human histiocytic lymphoma U937 cell line by CCK-8 (Cell Counting Kit-8) method. Compounds **8** and **16** showed significant selective cytotoxicity against U937 cell line with IC_50_ values of 4.9 and 8.8 μM, respectively. According to the literature, **16** was an inhibitor of VEGF signaling with the ability of inhibiting the proliferation of HUVECs induced by VEGF, bFGF or ECGS with IC_50_ values of 1.4, 2.8, and 6.2 μM [8], respectively, and showed weak cytotoxicity against human cancer cell lines SGC-7901 and BEL-7404 [14]. By comparison of the structures and cytotoxicities of these compounds, it seems that the substituent group at C-2 and the retainment of double bond between C-3 and C-4 are important for the cytotoxicity of this kind of chromone. 

**Table 4:** Cytotoxicity and Antibacterial Activity of Compounds **2**–**19**.

Compound	Zone of Inhibition (mm) ^a^		MIC (μg/mL)		Cytotoxicity (IC_50_ μM)				
	*E. coli*	*B. subtilis*	*E. coli*	*B. subtilis*	U937	Hela	MCF-7	HepG2	Huh7
**2**	- ^b^	-	-	-	55.5	96.1	172.3	73.8	>300
**3**	-	-	-	-	218.4	>300	>300	>300	>300
**4**	-	-	-	-	208.6	>300	>300	>300	>300
**5**	-	-	-	-	15.9	205.2	>300	>300	>300
**6**	-	-	-	-	192.7	>300	>300	>300	>300
**7**	-	-	-	-	287.2	>300	>300	>300	>300
**8**	13.8	16.5	64.0	32.0	4.9	24.8	38.5	60.5	53.3
**10**	-	10.0	-	256.0	^c^ NT	NT	NT	NT	NT
**12**	-	-	-	-	75.6	>300	>300	>300	>300
**14**	-	-	-	-	127.0	>300	>300	>300	>300
**15**	11.0	14.4	64.0	64.0	NT	NT	NT	NT	NT
**16**	15.8	17.5	64.0	64.0	8.8	60.0	102.2	52.7	133.3
**19**	11.4	13.6	64.0	128.0	NT	NT	NT	NT	NT
**^d^ Dox**					0.06	0.8	23.1	3.3	1.2
**^d^ PG**	31.8	43.3	2.0	2.0					

^a^ Zones of inhibition of compounds **8**, **10**, **15**, **16**, and **19** compounds at concentration of 25 μg/disc and penicillin at concentration of 10 μg/disc; ^b^ “-”: trace or no effect was observed; ^c^ “NT”: not tested; ^d^ Doxorubicin (Dox) and penicillin (PG) were used as positive control.

In addition, antilarval activities of the known compounds **12**–**18** were evaluated in settlement inhibition assays with laboratory-reared *Balanus amphitrite* larvae. Larval settlement bioassays revealed that **15** showed potent antilarval activity with EC_50_ value of 19.1 μg/mL and low toxicity with LC_50_/EC_50_ value of 70, and at the primal tested concentration of 25 μg/mL, **16** and **18** exhibited strong toxicity towards *B. amphitrite* larvae with lethality rates of 87.5% and 81.3% respectively, while other compounds showed a weak effect on larvae.

## 3. Experimental Section

### 3.1. General Experimental Procedure

Optical rotations were measured with an Anton Paar MCP 500 polarimeter. UV spectra were obtained using a Shimadzu UV-2600 UV-Vis spectrophotometer. CD spectra were measured with a Chirascan circular dichroism spectrometer (Applied Photophysics). IR spectra were measured with a Shimadzu IR Affinity-1 Fourier transform infrared spectrophotometer. ^1^H, ^13^C NMR and 2D NMR spectra were recorded on a Bruker AV-500 MHz NMR spectrometer with TMS as reference. MS spectroscopic data were obtained on a LCQDECA XP HPLC/MS^n^ spectrometer for ESIMS. High-resolution electrospray-ionization (HRESIMS) was performed on a UPLC/Q-TOF Micro MS spectrometer under 70 eV. Semi-preparative MPLC was performed on a CHEETAHTM MP 200. Semi-preparative HPLC was performed on a Shimadzu LC-20A preparative liquid chromatography with an YMC-Pack ODS column, 250 × 20 mm i.d., S-5 μm. Sephadex LH-20 (GE Healthcare) was used for CC. Silica gel (200–300 mesh) for columm CC and GF254 for TLC were obtained from the Qindao Marine Chemical Factory, Qindao, China.

### 3.2. Fungal Material

The fungal strain DFFSCS021 (GenBank access number JX156368) was isolated from a marine sediment sample collected in the South China Sea (19°00′368″N, 117°58′223″E; 3739 m depth), and identified as *Engydontium album* by ITS rDNA sequence homolgy (99% similarity with *E. album* isolate AHF (Genbank accession No. KC3111469)). The fungal strain was inoculated into a 7 mL centrifuge tube containing 1 mL potato glucose medium and cultured at 28 °C, shaking at 150 rpm for 7 days. Total genomic DNA was extracted from the strain, as described by Lai *et al.* [15]. From the genomic DNA (gDNA), the ITS region of rDNA was amplified by PCR using primers ITS1 (5′-TCCGTAGGT GAACCTGCGG-3′) and ITS4 (5′-TCCT CCGCT TATTG AT ATGC-3′). The primers were synthesized by SBS Genetech (China). The PCR mixtures consisted of 12.5 μL Taq premix (TakaRa, China), 0.25 μL (10 μM) of each primer (Taka Ra, China), 0.75 μL DMSO, 10.25 μL water, and 1 μL template gDNA. After denaturation at 94 °C for 6 min, amplification was performed with 30 cycles of 45 s at 94 °C, 45 s at 53 °C, 2 min at 72 °C and a final extension at 72 °C for 10 min. In addition, the macro- and micro-morphological characteristics of strain DFFSCS021 were examined (see Supporting Information Figure S71). The strain was deposited in the RNAM Center, South China Sea Institute of Oceanology, Chinese Academy of Sciences. 

### 3.3. Fermentation and Extraction

The fungal strain was inoculated in soluble starch medium (containing 10 g/L glucose, 10 g/L starch soluble, 1 g/L KH_2_PO_4_, 1 g/L MgSO_4_, 1 g/L bacterial peptone and 30 g/L sea salt) in 500 mL shake flask loading 120 mL as seed culture and incubated on a rotary shaker (200 rpm) at 28 °C for 2 days. Then, each of the seed cultures (100 mL) was transferred into autoclaved 5000 mL Erlenmeyer flasks that contained solid rice medium (each flask contained 500 g of commercially available rice, 2.5 g of yeast extract, 2.5 g of glucose, 22.5 g of sea salt, and 750 mL of water). Ultimately, the fungal stain was statically cultured in 5 kg rice medium at 26 °C for 28 days.

After incubation, the 5 kg rice culture was crushed and extracted with 80% acetone four times. The acetone extract was evaporated under reduced pressure to afford an aqueous solution, and then the aqueous solution was extracted with EtOAc to afford a 121 g brown crude gum.

### 3.4. Purification

The EtOAc extract (121 g) was chromatographed on a silica gel column eluting with a CHCl_3_/MeOH step gradient system at the ratios of 100:0, 99:1, 98:2, 95:5, 90:10, 80:20 and 0:100 (v/v) to yield nine fractions. Fraction 4 (5 g) was subjected to MPLC with an ODS column, eluting with MeOH/H_2_O (from 10:90 to 0:100, 120 min, 20 mL/min) to give five sub-fractions (Fr.4-1−Fr.4-5). Fr.4-1 was purified by semi-preparative reversed-phase (SP-RP) HPLC (YMC-Pack, ODS-A S-5 μm × 12 nm 250 × 20 mm i.d., 5 mL/min) eluting with C_2_H_3_N-H_2_O-TFA (46:54:0.03) to afford compounds **1** (10.3 mg, 0.085%) and **12** (20.9 mg, 0.173%). Fr.4-2 was purified by (SP-RP) HPLC eluting with C_2_H_3_N-H_2_O (38:62) to afford compound **9** (4.5 mg, 0.037%). Fr.4-3 was purified by (SP-RP) HPLC eluting with C_2_H_3_N-H_2_O-TFA (50:50:0.03) to afford compound **2** (7.9 mg, 0.065%). Fr.4-4 was purified by (SP-RP) HPLC eluting with C_2_H_3_N-H_2_O-TFA (45:55:0.03) to give compound **13** (16.7 mg, 0.138%). Fraction 6 (2.3 g) was isolated by MPLC with an ODS column, eluting with MeOH/H_2_O (from 20:80 to 0:100, 90 min, 20 mL/min) to give three sub-fractions (Fr.6-1−Fr.6-3) and compound **16** (18.5 mg, 0.153%). Fr.6-1 was purified by (SP-RP) HPLC eluting with MeOH-H_2_O (30:70) to give compound **15** (25.5 mg, 0.211%). Fr.6-2 was purified by (SP-RP) HPLC eluting with MeOH-H_2_O (60:40) to give compound **14** (15.5 mg, 0.128%). Fr.6-3 was purified by preparative TLC with a mobile phase of CHCl_3_-MeOH (9:1) to give compound **18** (14.3 mg, 0.118%). Fraction 7 (4.3 g) was subjected to silica gel CC eluting with a CHCl_3_/MeOH step gradient system at the ratios of 100:0, 98:2, 95:5, 90:10, 80:20, 50:50, and 0:100 (v/v) to give nine sub-fractions (Fr.7-1−Fr.7-9). Fr.7-2 was purified by (SP-RP) HPLC eluting with MeOH-H_2_O-TFA (38:62:0.03) to give compounds **6** (3.2 mg, 0.026%) and **7** (2.2 mg, 0.018%). Fr.7-5 was purified by (SP-RP) HPLC eluting with MeOH-H_2_O-TFA (52:48:0.03) to give compounds **10** (45.4 mg, 0.375%) and **11** (2.5 mg, 0.021%). Fr.7-8 was purified by (SP-RP) HPLC eluting with C_2_H_3_N-H_2_O-TFA (52:48:0.03) to give compound **19** (55.4 mg, 0.458%). Fraction 8 (3.9 g) was separated by MPLC with an ODS column, eluting with MeOH/H_2_O (from 10:90 to 0:100, 120 min, 20 mL/min) to give six sub-fractions (Fr.8-1−Fr.8-6). Fr.8-1 was purified by (SP-RP) HPLC eluting with C_2_H_3_N-H_2_O-TFA (12:88:0.03) to offer compound **15** (26.2 mg, 0.217%). Fr.8-2 was purified by (SP-RP) HPLC eluting with C_2_H_3_N-H_2_O-TFA (20:80:0.03) to afford compounds **3** (9.0 mg, 0.074%) and **16** (89.3 mg, 0.738%). Fr.8-3 was further purified by (SP-RP) HPLC eluting with C_2_H_3_N-H_2_O-TFA (35:65:0.03) to give compounds **5** (4.0 mg, 0.033%) and **8** (13.4 mg, 0.111%). Fr.8-4 was purified by (SP-RP) HPLC eluting with MeOH-H_2_O-TFA (38:62:0.03) to yield compounds **14** (8.0 mg, 0.066%) and **4** (4.6 mg, 0.038%). Fr.8-5 was purified by (SP-RP) HPLC eluting with C_2_H_3_N-H_2_O-TFA (38:62:0.03) to yield compound **17** (123.0 mg, 1.017%).

**Engyodontiumone A (1):** Pale yellow powder; UV (MeOH) λ_max_ (log ε) 385 (2.91), 293 (3.13), 263 (3.66), 236 (3.63) nm; FT-IR: 3356, 1678, 1647 cm^−1^; ^1^H and ^13^C NMR data, see Table 1 and Table 2; HRESIMS *m/ z* 331.0801 [M + H]^+^ (calcd for C_17_H_14_O_7_, 331.0812).

**Engyodontiumone B (2):** Yellow needles; UV (MeOH) λ_max_ (log ε) 372 (2.75), 287 (3.05), 259 (3.52), 234 (3.55) nm; FT-IR: 3394, 1674 cm^−1^; ^1^H and ^13^C NMR data, see Table 1 and Table 2; HRESIMS *m/ z* 335.0309 [M + H]^+^ (calcd for C_17_H_11_ClO_6_, 335.0317).

**Engyodontiumone C (3):** Brown needles; ${\left[\alpha \right]}_{D}^{25}$ 5 (*c* 0.5, MeOH); UV (MeOH) λ_max_ (log ε) 328 (3.38), 239 (4.13), 203 (4.06) nm; FT-IR: 3417, 1728, 1697, 1651 cm^−1^; ^1^H and ^13^C NMR data, see Table 1 and Table 2; HRESIMS *m/z* 321.0972 [M + H]^+^ (calcd for C_16_H_16_O_8_ , 321.0969).

**Engyodontiumone D (4):** Brown needles; ${\left[\alpha \right]}_{D}^{25}$ 14 (*c* 0.3, MeOH); UV (MeOH) λ_max_ (log ε) 325 (3.38), 322 (3.38), 238 (4.07), 230 (4.05), 202 (4.07) nm; FT-IR: 3456,1728,1658, 1627 cm^−1^; ^1^H and ^13^C NMR data, see Table 1 and Table 2; HRESIMS *m/z* 321.0974 [M + H]^+^ (calcd for C_16_H_16_O_7_, 321.0969).

**Engyodontiumone E (5):** Brown needles; ${\left[\alpha \right]}_{D}^{25}$ 0 (*c* 0.4, MeOH); UV (MeOH) λ_max_ (log ε) 329 (3.41), 258 (3.97), 238 (4.10), 203 (4.07) nm; FT-IR: 3336,1724,1654, 1620 cm^−1^; ^1^H and ^13^C NMR data, see Table 1 and Table 2; HRESIMS *m/z* 321.0968 [M + H]^+^ (calcd for C_16_H_16_O_8_, 321.0969).

**Engyodontiumone F (6):** Brown needles; ${\left[\alpha \right]}_{D}^{25}$ 0 (*c* 0.3, MeOH); UV (MeOH) λ_max_ (log ε) 328 (3.42), 238 (4.17), 203 (4.06) nm; FT-IR: 3334, 1732, 1651 cm^−1^; ^1^H and ^13^C NMR data, see Table 1 and Table 2; HRESIMS *m/z* 337.0922 [M + H]^+^ (calcd for C_16_H_16_O_8_ , 337.0918).

**Engyodontiumone G (7):** Brown needles; ${\left[\alpha \right]}_{D}^{25}$ 0 (*c* 0.2, MeOH); UV (MeOH) λ_max_ (log ε) 328 (3.14), 238 (3.94), 202 (3.94) nm; FT-IR: 3336, 1728, 1651 cm^−1^; ^1^H and^13^C NMR data, see Table 1 and Table 2; HRESIMS *m/z* 337.0922 [M + H]^+^ (calcd for C_16_H_16_O_8_ , 337.0918).

**Engyodontiumone H (8):** Brown needles; ${\left[\alpha \right]}_{D}^{25}$ −56 (*c* 1, MeOH); UV (MeOH) λ_max_ (log ε) 344 (3.31), 272 (3.98), 213 (3.90), 203 (3.97) nm; FT-IR: 3420, 1735, 1658 cm^−1^; ^1^H and^13^C NMR data, see Table 1 and Table 2; HRESIMS *m/z* 319.0922 [M + H]^+^ (calcd for C_16_H_14_O_7_ , 319.0812).

**2-methoxyl cordyol C (9):** Yellow amorphous solid; UV (MeOH) λ_max_ (log ε) 360 (2.64), 356 (2.64), 274 (3.20) nm; FT-IR: 3271, 1678 cm^−1^; ^1^H NMR (500 MHz, DMSO-*d*_6_) δ_H_: 9.35 (2H, brs, OH-3/3′), 6.49 (1H, s, H-6), 6.26 (1H, s, H-6′), 6.24 (1H, s, H-4), 6.17 (1H, s, H-4′), 6.06 (1H, s, H-3′) , 3.60 (3H, s, H-8), 2.16 (3H, s, H-7′) and 2.14 (3H, s, H-7); ^13^C NMR (125 MHz, DMSO-*d*_6_) δ_C_: 158.7 (s, C-1′), 158.2 (s, C-3′), 151.0 (s, C-3) , 148.3 (s, C-1), 139.7 (s, C-5′), 137.3 (s, C-2), 133.1 (s, C-5), 113.2 (d, C-6), 112.4 (d, C-4), 110.1 (d, C-6′), 108.0 (d, C-4′), 100.9 (d, C-2′), 60.1 (q, C-8), 21.1 (q, C-7′) and 20.6 (q, C-7). HRESIMS *m/z* 261.1122 [M + H]^+^ (calcd for C_15_H_16_O_4_ , 261.1121).

**Engyodontiumone I (10):** Yellow gum; UV (MeOH) λ_max_ (log ε) 302 (3.40), 255 (3.64), 211 (4.16) nm; FT-IR: 3402, 3132, 1689, 1651, 979, 948 cm^−1^; ^1^H NMR (500 MHz, DMSO-*d*_6_) δ_H_: 7.40 (1H, d, *J* = 1.5 Hz, H-2), 7.33 (1H, dd, *J* = 1.5, 7.5 Hz, H-4), 7.09 (1H, d, *J* = 7.5 Hz, H-5), 5.49 (1H, m, H-5), 2.18 (2H, m, H-9), 1.93 (3H, s, H-14), 1.48 (2H, m, H-10), 1.11 (6H, s, H-12/13); ^13^C NMR (125 MHz, DMSO-*d*_6_) δ_C_: 167.2 (C, C-15), 154.3 (C, C-1), 136.7 (C, C-3), 133.4 (C, C-6), 130.8 (CH, C-8), 129.9 (CH, C-7), 129.2 (CH, C-5), 120.1 (CH, C-4), 116.1 (CH, C-2), 68.8 (C, C-11), 43.1 (CH_2_, C-10), 29.2 (CH_3_, C-12/13), 23.1 (CH_2_, C-9), 16.4 (CH_3_, C-14); HRESIMS *m/z* 265.1426 [M + H]^+^ (calcd for C_15_H_20_O_4_ , 265.1434).

**Engyodontiumone J (11):** Pale yellow gum; UV (MeOH) λ_max_ (log ε) 297 (3.26), 244 (3.57), 207 (4.10) nm; FT-IR: 3344, 1689, 1612 cm^−1^; ^1^H NMR (500 MHz, DMSO-*d*_6_) δ_H_: 7.42 (1H, d, *J* = 2.0 Hz, H-2), 7.35 (1H, dd, *J* = 2.0, 6.0 Hz, H-4), 7.03 (1H, d, *J* = 6.0Hz, H-5), 5.44 (1H, m, H-5), 2.18 (2H, m, H-9), 1.93 (3H, m, H-14), 1.81 (2H, s, H-9), 1.37 (3H, s, H-10), 0.95 (6H, s, H-12/13); ^13^C NMR (125 MHz, DMSO-*d*_6_) δ_C_: 167.2 (C, C-15), 154.2 (C, C-1), 133.3 (C, C-3), 132.5 (C, C-6), 130.1 (CH, C-8), 129.6 (CH, C-7), 128.7 (CH, C-5), 119.7 (CH, C-4), 116.0 (CH, C-2), 68.5 (C, C-11), 43.4 (CH_2_, C-10), 29.0 (CH_3_, C-12/13), 24.0 (CH_2_, C-9), 23.8 (CH_3_, C-14); HRESIMS *m/z* 265.1428 [M + H]^+^ (calcd for C_15_H_20_O_4_ , 265.1434).

### 3.5. Cytotoxicity

Chromones (**2**–**8**, **12**–**14**, **16**) were tested for cytotoxicity against Hela, MCF-7, HepG2 and Huh7 human carcinoma cell lines using a MTT (3-(4,5-dimethyl-2-thiazolyl)-2,5-diphenyl-2-*H*-tetrazolium bromide) method, and against U937 human carcinoma cell line using a CCK-8 (2-(2-methoxy-4-nitrophenyl)-3-(4-nitrophenyl)-5-(2,4-disulfophenyl)-2*H*-tetrazolium, monosodium salt) method. The media for U937 and the other four cell lines were RPMI1640 and DMEM, respectively. Cells in the logarithmic phase were seeded into 96-well plates in a 100 μL volume of medium, maintained at 37 °C in a 5% CO_2_ incubator overnight, then treated with the corresponding compounds or vehicle control at the indicated concentration. After 48 h of incubation, 5 μL of MTT or CCK-8 was added into the 96-well plates and incubated for additional 2–3 h, then the absorbance was measured in a microplate reader at 450 nm and 650 nm. Cell toxicity was evaluated as the ratio of the absorbance of the sample to that of the control. The IC_50_ were further calculated using Graphpad Prism 5 (Graphpad Software, Inc., San Diego, CA, USA).

### 3.6. Antibacterial Activities

Antibacterial activities of **1**−**19** were tested against common bacteria *Bacillus subtilis* and *Escherichia coli* using a standard disc diffusion assay [16]. Briefly, the tested bacteria were cultured on nutrient agar slants at 37 °C for 1−2 days, then a 40 μL cell suspension (10^8^ CFU/mL) was mixed with 20 mL of corresponding nutrient agar and poured in 100 mm sterile petri plates. Sterile 6 mm diameter circular discs of filter paper were loaded with 25 μg of tested compound dissolved in methanol, evaporated to dryness, and then placed onto the seeded bacterial plates. An additional set of discs with 10 μg penicillin was used as the positive controls. The agar plates were incubated for 18 h at 37 °C. The inhibition zones were measured. 

The minimum inhibitory concentration (MIC) of active compounds were determined by a microbroth dilution method [17]. Briefly, the tested bacteria were cultured on starch culture-medium including tryptone (10 g/L), yeast extract (5 g/L), NaCl (10 g/L) at 37 °C for 1–2 days on a rotary shaker (200 rpm), then scraped to make a cell suspension (10^5^ CFU/mL) in the starch culture-medium. Tested samples and penicillin (antibiotic control) were dissolved in DMSO and dispensed into 96-well plates to create a dilution series (half fold dilutions ranging from 1024 to 0.5 µg/mL) in a 100 μL volume of starch culture-medium. Following incubation at 37 °C for 12–24 h, the tubes were examined for visible bacterial growth as evidenced by turbidity. The lowest concentration of test compound that prevented 100% growth represented the minimal inhibitory concentration (MIC). The experiment was run in 3 replicates.

### 3.7. Barnacle Balanus amphitrite Larval Settlement Bioassays

Larval settlement bioassays were performed using sterile 24-well polystyrene plates. Tested samples were dissolved in DMSO to a concentration of 25 μg mL^−1^ for preliminary bioassay. To define the EC_50_ values of antilarval compounds found in the preliminary bioassay, different dilutions of the tested compounds were further prepared to the concentrations ranging from 1 to 200 μg mL^−1^ in autoclaved FSW (filtered seawater). About 20 competent larvae were added to each well in 1 mL of the test solution. Wells containing only FSW with DMSO served as the controls. The plates were incubated at 27 °C for 24 h. The percentage of larval settlement was determined by counting the settled, live individuals under a dissecting microscope and expressing the result as a proportion of the total number of larvae in the well [18].

## 4. Conclusions

In conclusion, eight new chromones (**1**–**8**) and three new phenol derivatives (**9**–**11**) together with eight known polyketides were isolated from the culture medium of the deep-sea-derived fungus *Engyodontium album* DFFSCS021. Among then, **8** and **16** showed significant selective cytotoxicity against U937 cell line with IC_50_ < 10 μM. This is the first time to report that **8**, **15** and **16** had mild antibacterial activity against *E. coli* and *B. subtilis* with MIC ≤ 64.0 μg/mL, and **15** showed potent antilarval activity against barnacle *B. amphitrite* larval settlement with EC_50_ value of 19.1 μg/mL and low toxicity.

## Supplementary Files

Supplementary File 1

## References

[1] Blunt J.W., Copp B.R., Munro M.H.G., Northcote P.T., Prinsep M.R. (2011). Marine natural products. Nat. Prod. Rep..

[2] Skropeta D. (2008). Deep-sea natural products. Nat. Prod. Rep..

[3] Peng J., Zhang X.Y., Tu Z.C., Xu X.Y., Qi S.H. (2013). Alkaloids from the deep-sea-derived fungus *Aspergillus westerdijkiae* DFFSCS013. J. Nat. Prod..

[4] Hamasaki T., Sato Y., Hatsuda Y. (1975). Structure of sydowinin A, sydowinin B, and sydowinol, metabolites from *Aspergillus sydowi*. Agric. Biol. Chem..

[5] Healy P.C., Hocking A., Tran-Dinh N., Pitt J.I., Shivas R.G., Mitchell J.K., Kotiw M., Davis R.A. (2003). Xanthones from a microfungus of the genus *Xylaria*. Phytochemistry.

[6] Little A., Porco J.J. (2012). Total syntheses of graphisin A and sydowinin B. Org. Lett..

[7] Trisuwan K., Rukachaisirikul V., Kaewpet M., Phongpaichit S., Hutadilok-Towatana N., Preedanon S., Sakayaroj J., Rukachaisirikul V. (2011). Sesquiterpene and xanthone derivatives from the sea fan-derived fungus *Aspergillus sydowii* PSU-F154. J. Nat. Prod..

[8] Kim H.S., Park I.Y., Park Y.J., Lee J.H., Hong Y.S., Lee J.J. (2002). A novel dihydroxanthenone, AGI-B4 with inhibition of VEGF-induced endothelial cell growth. J. Antibiot.

[9] Philip M.H., Bijay K.S., Iwona T.S., Jeffrey H.B. (1995). Synthesis and herbicidal activity of cyperin. J. Agric. Food Chem..

[10] Bunyapaiboonsri T., Yoiprommarat S., Intereya K., Kocharin K. (2007). New diphenyl ethers from the insect pathogenic fungus *Cordyceps* sp. BCC 1861. Chem. Pharm. Bull..

[11] Takashi H., Kouzou N., Yuichi H. (1978). Two new metabolites, sydonic acid and hydroxysydonic acid, from *Aspergillus sydowi*. Agric. Biol. Chem..

[12] Ratnayake R., Lace E., Tennant S., Gill J.H., Capon R.J. (2007). Kibdelones: Novel anticancer polyketides from a rare Australian actinomycete. Chem. Eur. J..

[13] Lange G.L., Lee M. (1986). ^13^C NMR determination of the configuration of methyl substituted double bonds in medium- and large-ring terpenoids. Mag. Res. Chem..

[14] Tan Q.W., Ouyang M.A., Shen S., Li W. (2012). Bioactive metabolites from a marine-derived strain of the fungus *Neosartorya fischeri*. Nat. Prod. Res..

[15] Lai X., Cao L., Tan H., Fang S., Huang Y., Zhou S. (2007). Fungal communities from methane hydrate-bearing deep-sea marine sediments in South China Sea. ISME J..

[16] Lorian V. (1980). Antibiotics in Laboratory Medicine: The Disc Susceptibility Test.

[17] Brantner A., Grein E. (1994). Antibacterial activity of plant extracts used externally in traditional medicine. J. Ethnopharmacol..

[18] Qi S.H., Zhang S., Qian P.Y., Xiao Z.H., Li M.Y. (2006). Ten new antifouling briarane diterpenoids from the South China Sea gorgonian *Junceella juncea*. Tetrahedron.

