# Supplementary material for: Cytotoxic Polyketides from the Deep-Sea-Derived Fungus Engyodontium album DFFSCS021
Source: Mar Drugs. 2014 Dec 9;12(12):5902–15. doi: 10.3390/md12125902 (PMC4278208; doi:10.3390/md12125902)
Supplement: Supplementary File 1 [file marinedrugs-12-05902-s001.pdf]

Supplementary Information

Contents **page**

Figure S1. ^{1}H NMR spectrum of **1** 3
Figure S2. ^{13}C NMR spectrum of **1** 3
Figure S3. HMBC spectrum of **1** 4
Figure S4. HRESIMS spectrum of **1** 4
Figure S5. IR spectrum of **1** 5
Figure S6. ^{1}H NMR spectrum of **2** 5
Figure S7. ^{13}C NMR spectra of **2** 6
Figure S8. HMBC spectrum of **2** 6
Figure S9. HRESIMS spectrum of **2** 7
Figure S10. IR spectrum of **2** 7
Figure S11. ^{1}H NMR spectrum of **3** 8
Figure S12. ^{13}C NMR spectra of **3** 8
Figure S13. HSQC spectrum of **3** 9
Figure S14. HMBC spectrum of **3** 9
Figure S15. NOESY spectrum of **3** 10
Figure S16. HRESIMS spectrum of **3** 10
Figure S17. IR spectrum of **3** 11
Figure S18. ^{1}H NMR spectrum of **4** 11
Figure S19. ^{13}C NMR spectra of **4** 12
Figure S20. HSQC spectrum of **4** 12
Figure S21. HMBC spectrum of **4** 13
Figure S22. NOESY spectrum of **4** 13
Figure S23. HRESIMS spectrum of **4** 14
Figure S24. IR spectrum of **4** 14
Figure S25. ^{1}H NMR spectrum of **5** 15
Figure S26. ^{13}C NMR spectra of **5** 15
Figure S27. HMBC spectrum of **5** 16
Figure S28. NOESY spectrum of **5** 16
Figure S29. HRESIMS spectrum of **5** 17
Figure S30. IR spectrum of **5** 17
Figure S31. ^{1}H NMR spectrum of **6** 18
Figure S32. ^{13}C NMR spectra of **6** 18
Figure S33. HSQC spectrum of **6** 19
Figure S34. HMBC spectrum of **6** 19
Figure S35. ^{1}H-^{1}H COSY spectrum of **6** 20
Figure S36. NOESY spectrum of **6** 20
Figure S37. HRESIMS spectrum of **6** 21
Figure S38. IR spectrum of **6** 21
Figure S39. ^{1}H NMR spectrum of **7** 22
Figure S40. ^{13}C NMR spectra of **7** 22

Figure S41. HSQC spectrum of **7** 23
Figure S42. HMBC spectrum of **7** 23
Figure S43. NOESY spectrum of **7** 24
Figure S44. HRESIMS spectrum of **7** 24
Figure S45. IR spectrum of **7** 25
Figure S46. ^{1}H NMR spectrum of **8** 25
Figure S47. ^{13}C NMR spectra of **8** 26
Figure S48. HMBC spectrum of **8** 26
Figure S49. NOESY spectrum of **8** 27
Figure S50. HRESIMS spectrum of **8** 27
Figure S51. IR spectrum of **8** 28
Figure S52. ^{1}H NMR spectrum of **9** 28
Figure S53. ^{13}C NMR spectra of **9** 29
Figure S54. HMBC spectrum of **9** 29
Figure S55. HRESIMS spectrum of **9** 30
Figure S56. IR spectrum of **9** 30
Figure S57. ^{1}H NMR spectrum of **10** 31
Figure S58. ^{13}C NMR spectra of **10** 31
Figure S59. HMBC spectrum of **10** 32
Figure S60. ^{1}H-^{1}H COSY spectrum of **10** 32
Figure S61. HRESIMS spectrum of **10** 33
Figure S62. IR spectrum of **10** 33
Figure S63. ^{1}H NMR spectrum of **11** 34
Figure S64. ^{13}C NMR spectra of **11** 34
Figure S65. HMBC spectrum of **11** 35
Figure S66. HRESIMS spectrum of **11** 35
Figure S67. IR spectrum of **11** 36
Figure S68. HPLC analysis of compound **5** on a chiral column (IPA/n-hexane 30:70) 37
Figure S69. HPLC analysis of compound **6** on a chiral column (IPA/n-hexane 30:70) 38
Figure S70. HPLC analysis of compound **7** on a chiral column (IPA/n-hexane 22:78) 39
Figure S71. Macro- and micro-morphological observations of strain DFFSCS021 40

Figure S1. ^{1}H NMR spectrum of **1**.

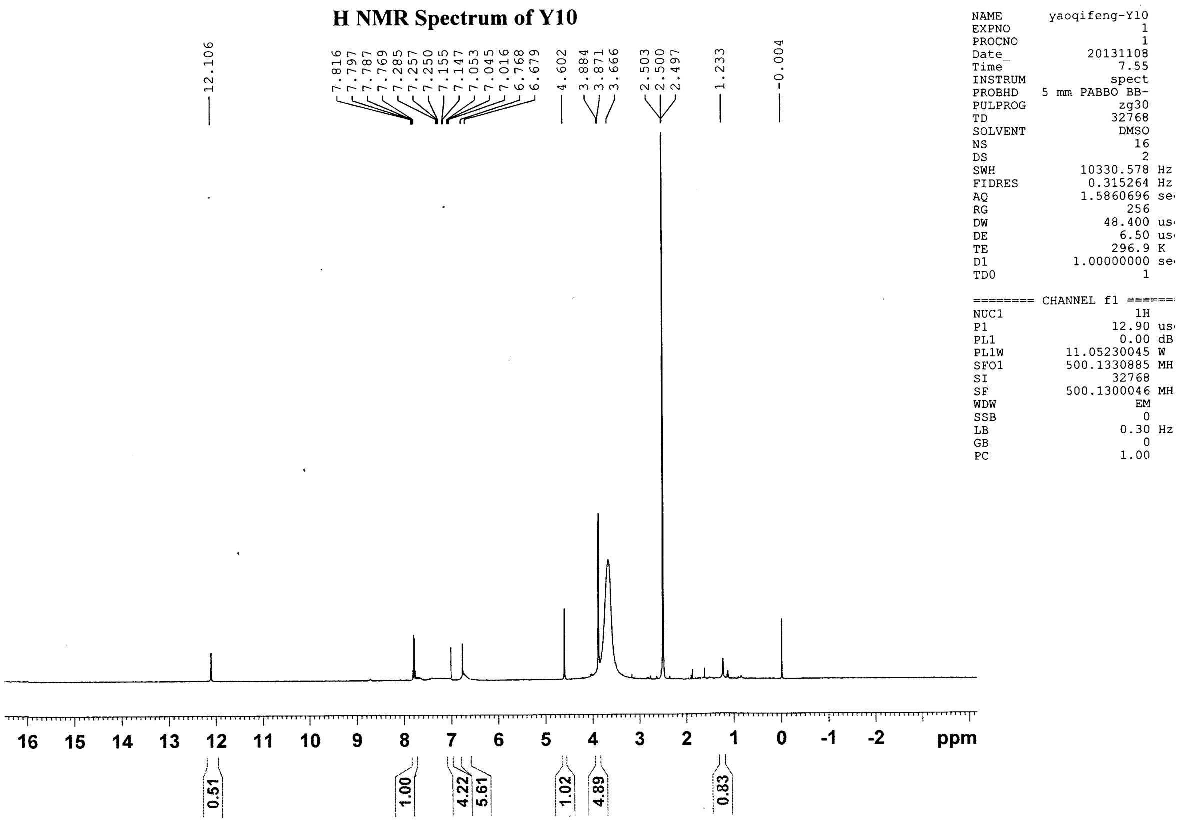

Figure S2. ^{13}C NMR spectrum of **1**.

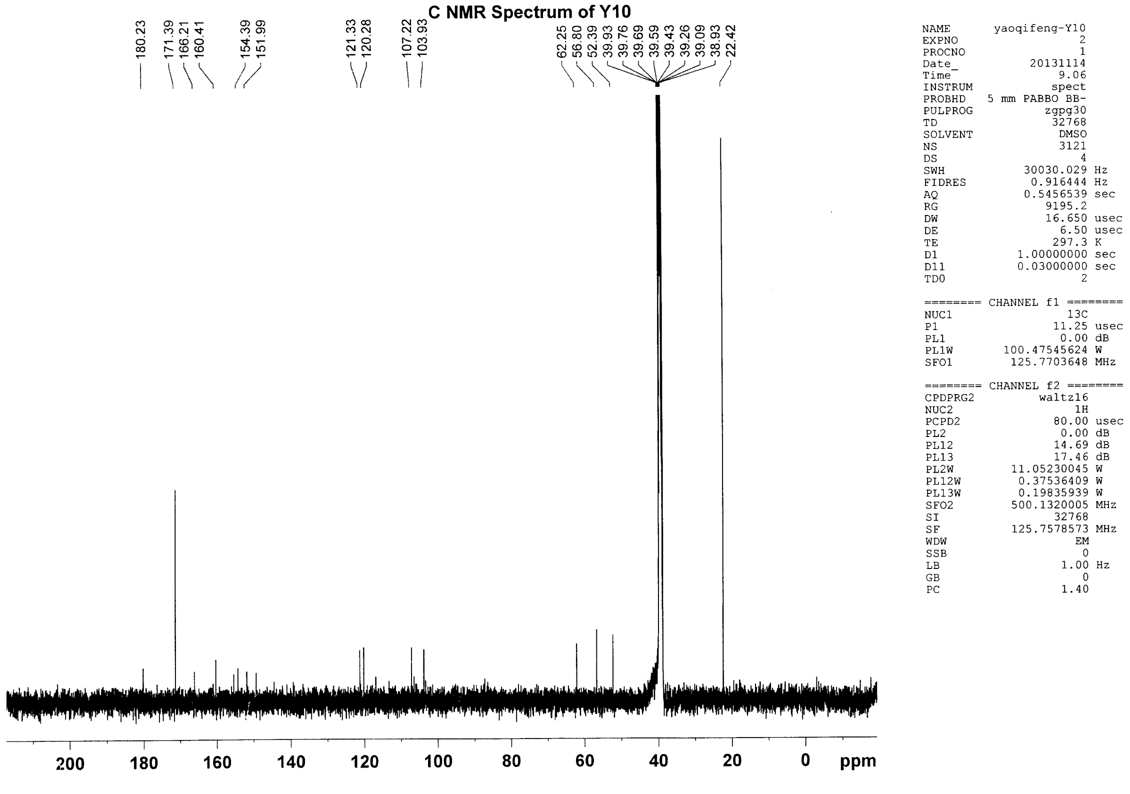

Figure S3. HMBC spectrum of **1** and partial enlarged view for the assignment of C-13.

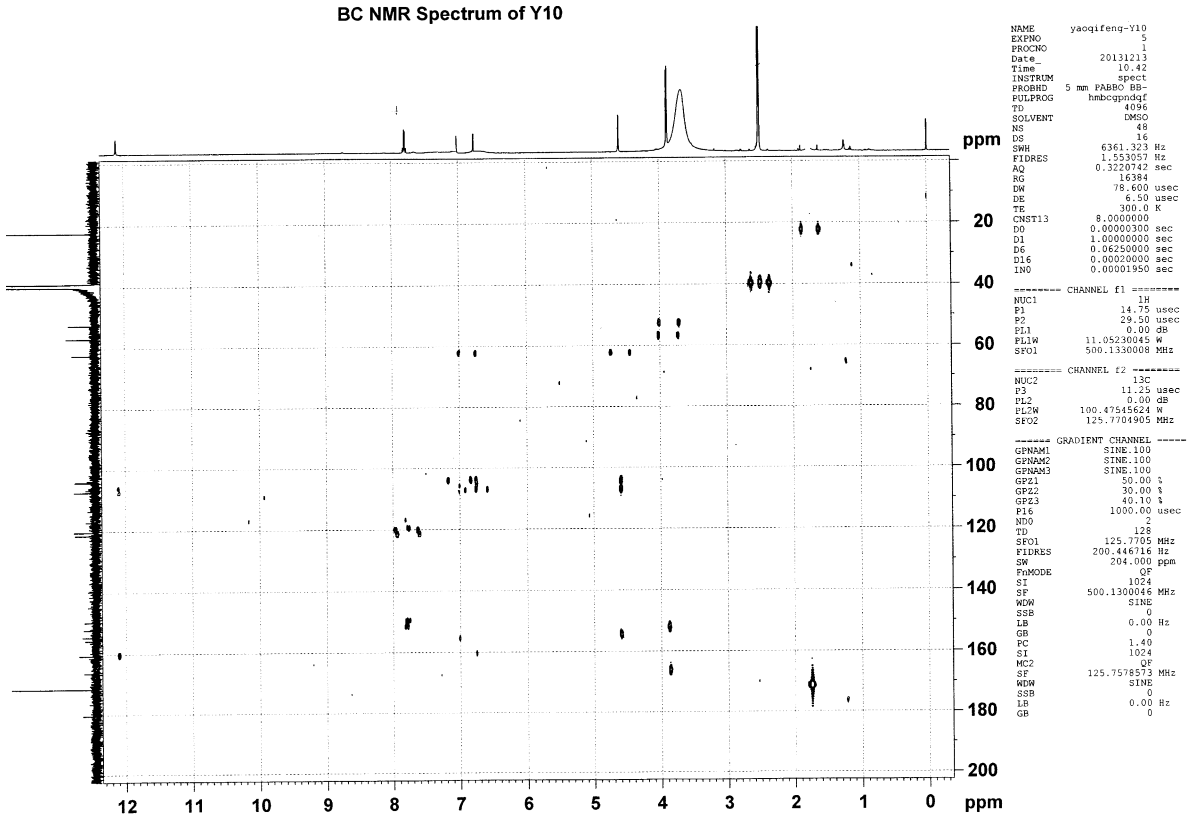

Figure S4. HRESIMS spectrum of **1**.

Mass Spectrum SmartFormula Report

Analysis Info

Analysis Name	D:\Data\MS\data\201401\yaoqifeng_y10_pos.d	Acquisition Date	1/17/2014 10:36:17 AM
Method	POS_100-2000_Dirrect Infusion.m	Operator	SCSIO
Sample Name	SCSIO	Instrument / Ser#	maXis 29
Comment			

Acquisition Parameter

Source Type	ESI	Ion Polarity	Positive	Set Nebulizer	0.3 Bar
Focus	Not active	Set Capillary	4500 V	Set Dry Heater	180 °C
Scan Begin	100 m/z	Set End Plate Offset	-500 V	Set Dry Gas	4.0 l/min
Scan End	2000 m/z	Set Collision Cell RF	2000.0 Vpp	Set Divert Valve	Waste

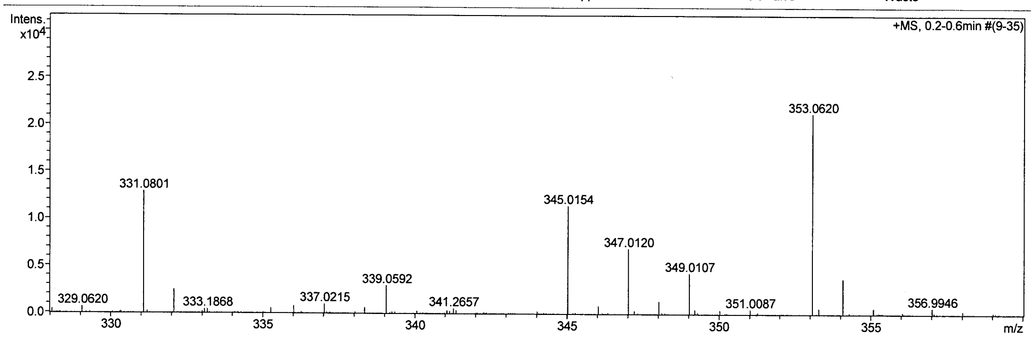

Meas. m/z	#	Formula	Score	m/z	err [mDa]	err [ppm]	mSigma	rdb	e⁻ Conf	N-Rule
331.0801	1	C 17 H 15 O 7	66.26	331.0812	1.1	3.4	5.3	10.5	even	ok
	2	C 14 H 7 N 10 O	100.00	331.0799	-0.2	-0.6	8.6	16.5	even	ok
	3	C 13 H 11 N 6 O 5	39.87	331.0785	-1.6	-4.7	18.0	11.5	even	ok
353.0620	1	C 17 H 14 Na O 7	65.67	353.0632	1.2	3.3	5.6	10.5	even	ok
	2	C 13 H 10 N 6 Na O 5	50.84	353.0605	-1.5	-4.3	7.4	11.5	even	ok
	3	C 14 H 6 N 10 Na O	100.00	353.0618	-0.2	-0.5	10.3	16.5	even	ok
661.1523	1	C 34 H 29 O 14	18.74	661.1552	2.9	4.4	15.0	20.5	even	ok
	2	C 28 H 13 N 20 O 2	100.00	661.1525	0.2	0.3	22.8	32.5	even	ok
	3	C 26 H 21 N 12 O 10	15.76	661.1498	-2.5	-3.7	39.9	22.5	even	ok
683.1344	1	C 26 H 20 N 12 Na O 10	21.80	683.1318	-2.6	-3.9	4.1	22.5	even	ok

Bruker Compass DataAnalysis 4.0 printed: 1/17/2014 10:48:18 AM Page 1 of 2

Figure S5. IR spectrum of **1**.

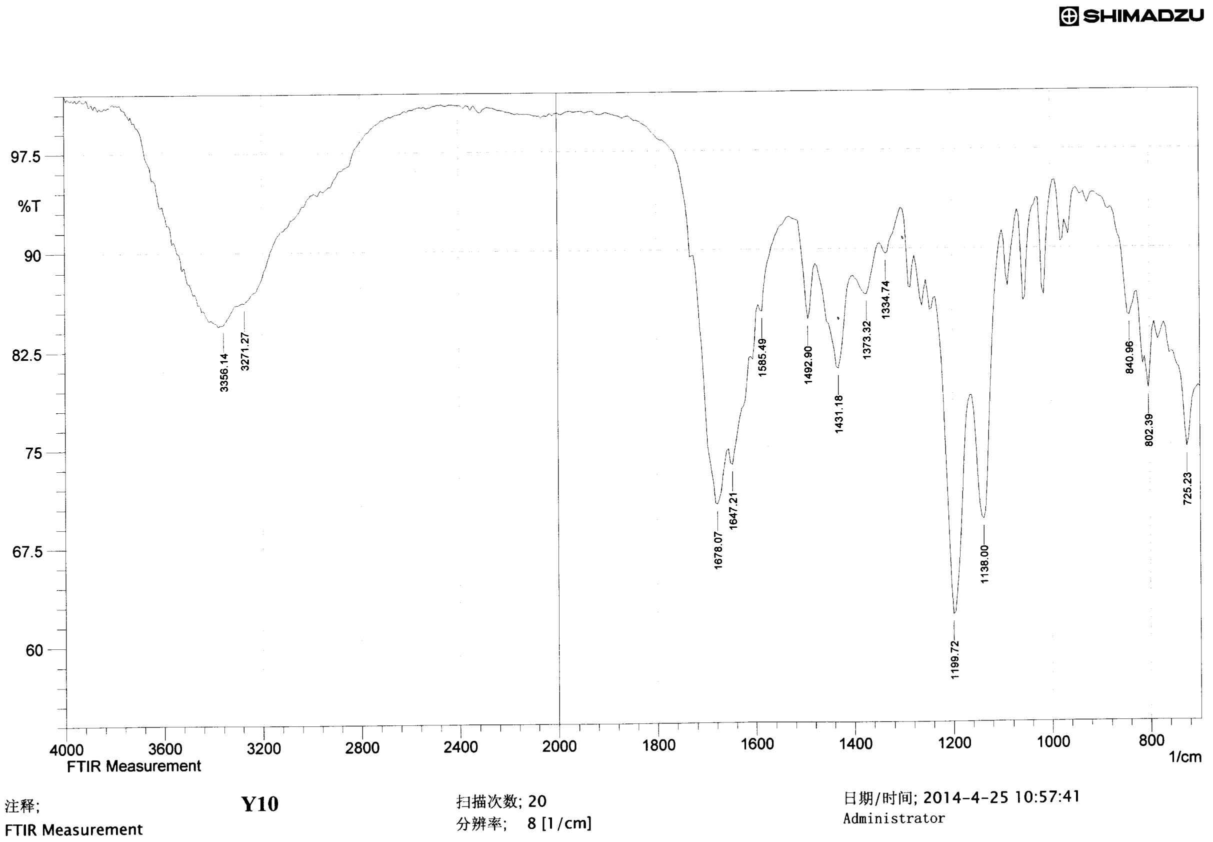

Figure S6. ^{1}H NMR spectrum of **2**.

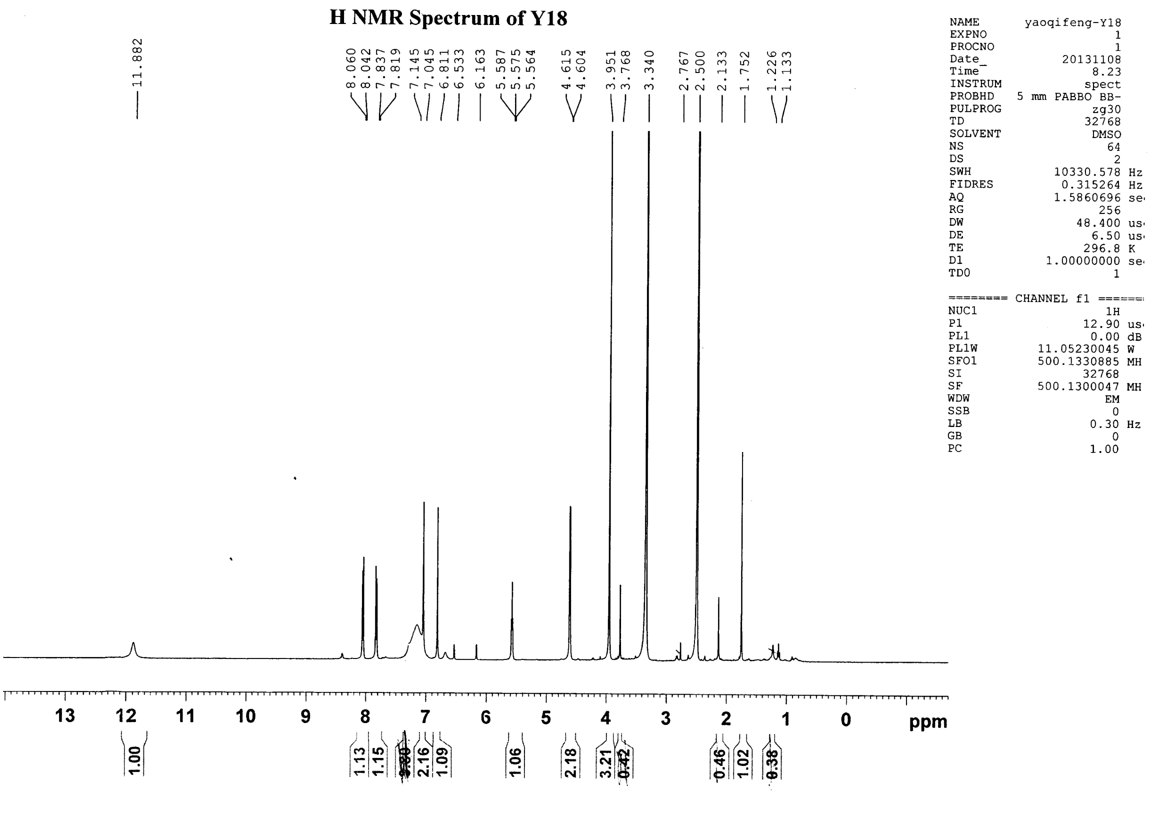

Figure S7. ^{13}C NMR spectrum of **2**.

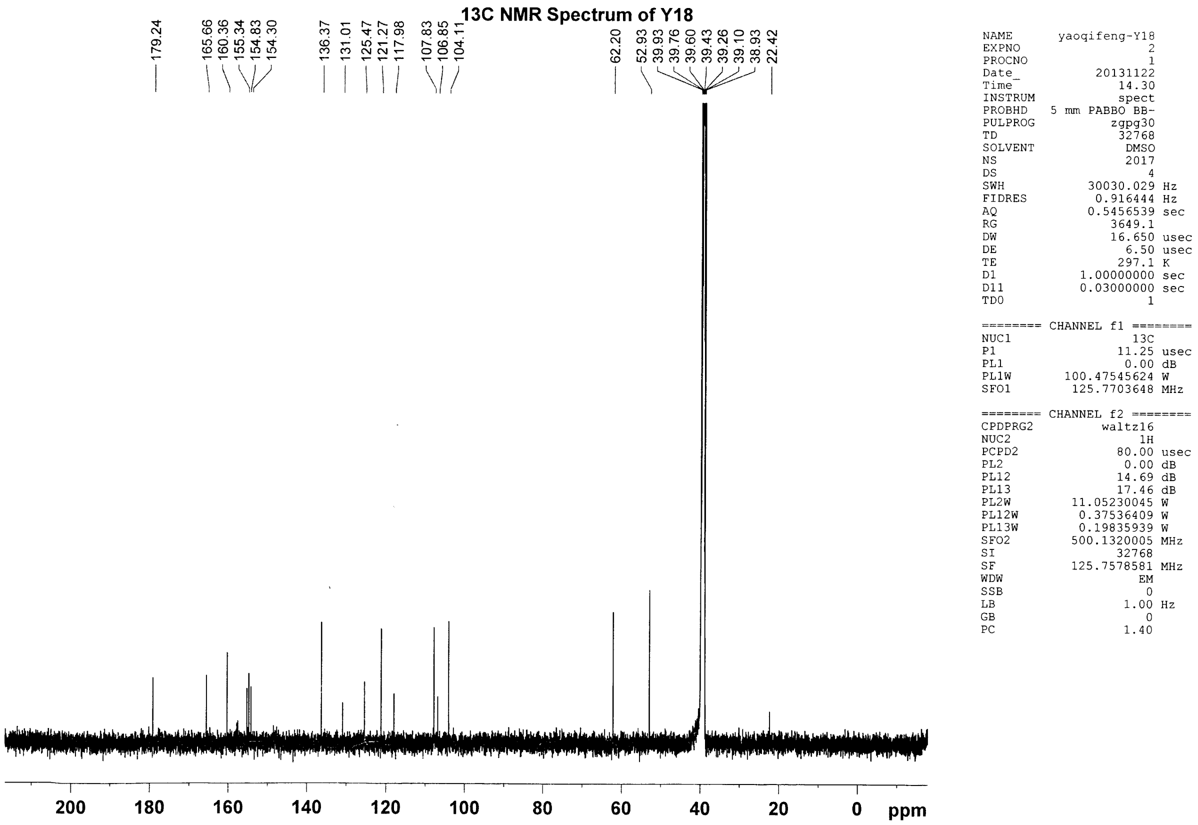

Figure S8. HMBC spectrum of **2**.

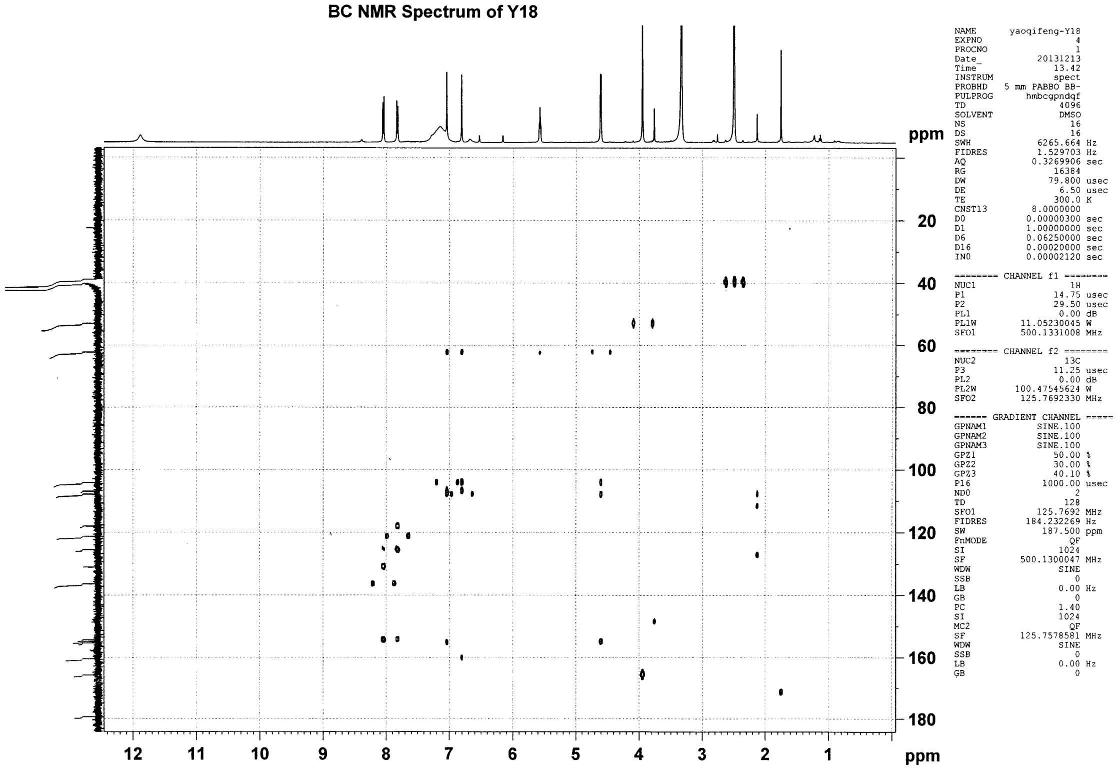

Figure S9. HRESIMS spectrum of **2**.

Mass Spectrum SmartFormula Report

Analysis Info

Analysis Name	D:\Data\MS\data\201312\yaoqifeng-Y18_pos.d	Acquisition Date	12/4/2013 4:47:12 PM
Method	POS_100-2000_Dirrect Infusion.m	Operator	SCSIO
Sample Name	SCSIO	Instrument / Ser#	maXis 29
Comment			

Acquisition Parameter

Source Type	ESI	Ion Polarity	Positive	Set Nebulizer	0.3 Bar
Focus	Not active	Set Capillary	4500 V	Set Dry Heater	180 °C
Scan Begin	100 m/z	Set End Plate Offset	-500 V	Set Dry Gas	2.0 l/min
Scan End	1500 m/z	Set Collision Cell RF	2000.0 Vpp	Set Divert Valve	Waste

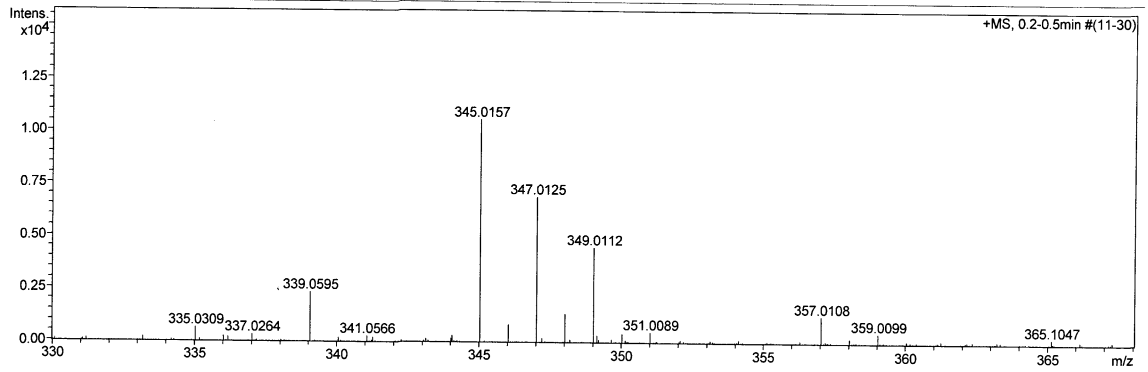

Meas. m/z	#	Formula	Score	m/z	err [mDa]	err [ppm]	mSigma	rdb	e⁻ Conf	N-Rule
335.0309	1	C 16 H 12 Cl O 6	100.00	335.0317	0.8	2.4	119.6	10.5	even	ok
357.0108	1	C 16 H 11 Cl Na O 6	20.43	357.0136	2.8	7.9	12.4	10.5	even	ok
691.0362	1	C 32 H 22 Cl 2 Na O 12	37.63	691.0381	1.9	2.7	64.4	20.5	even	ok

Figure S10. IR spectrum of **2**.

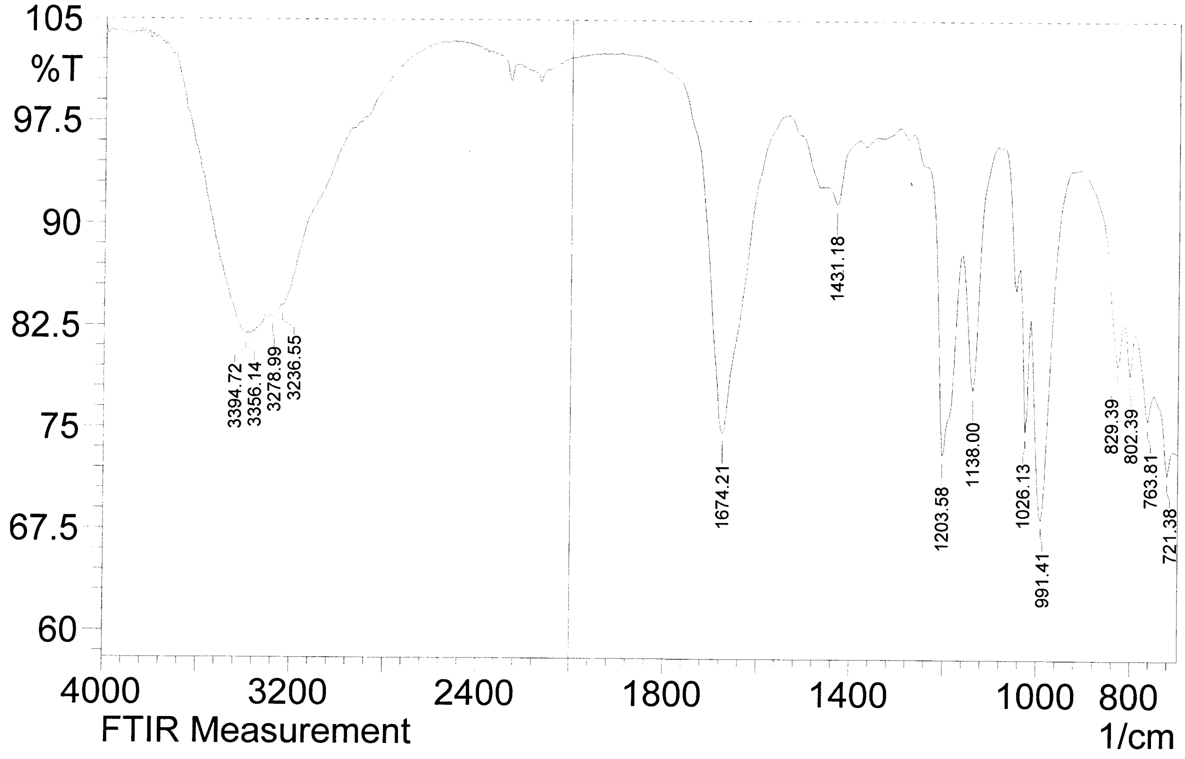

SCSIO Y18 2014-4-25 10:30:59

Figure S11. ^{1}H NMR spectrum of **3**.

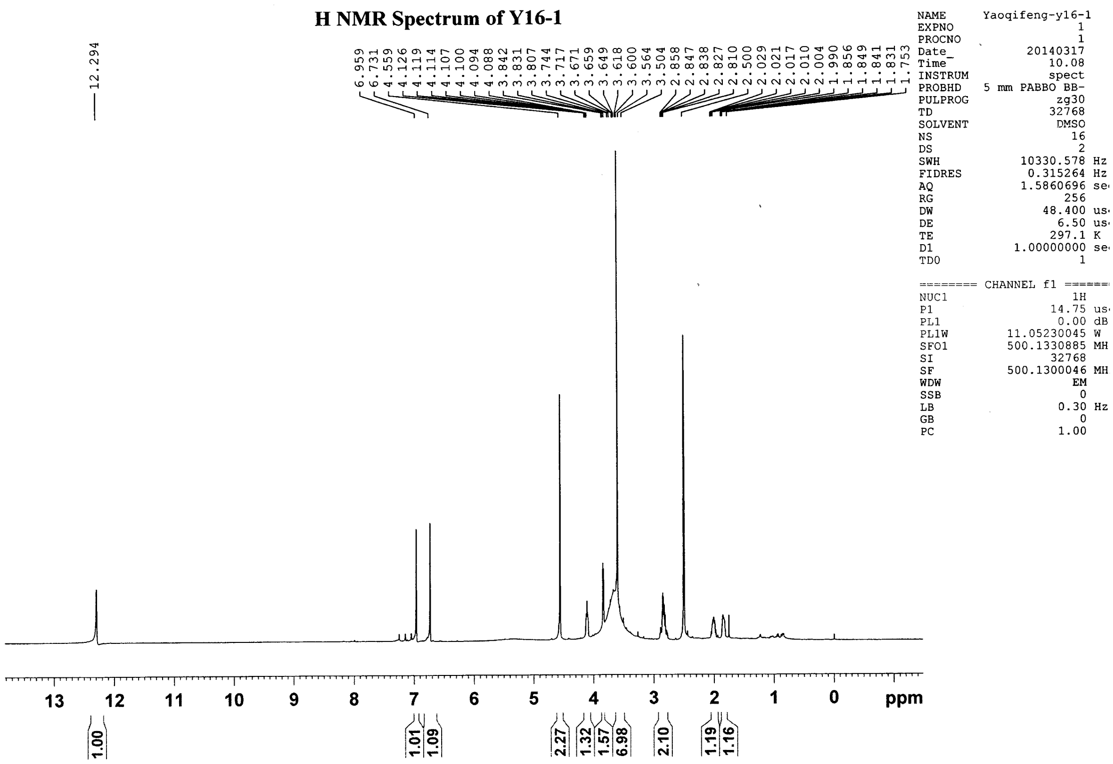

Figure S12. ^{13}C NMR spectrum of **3**.

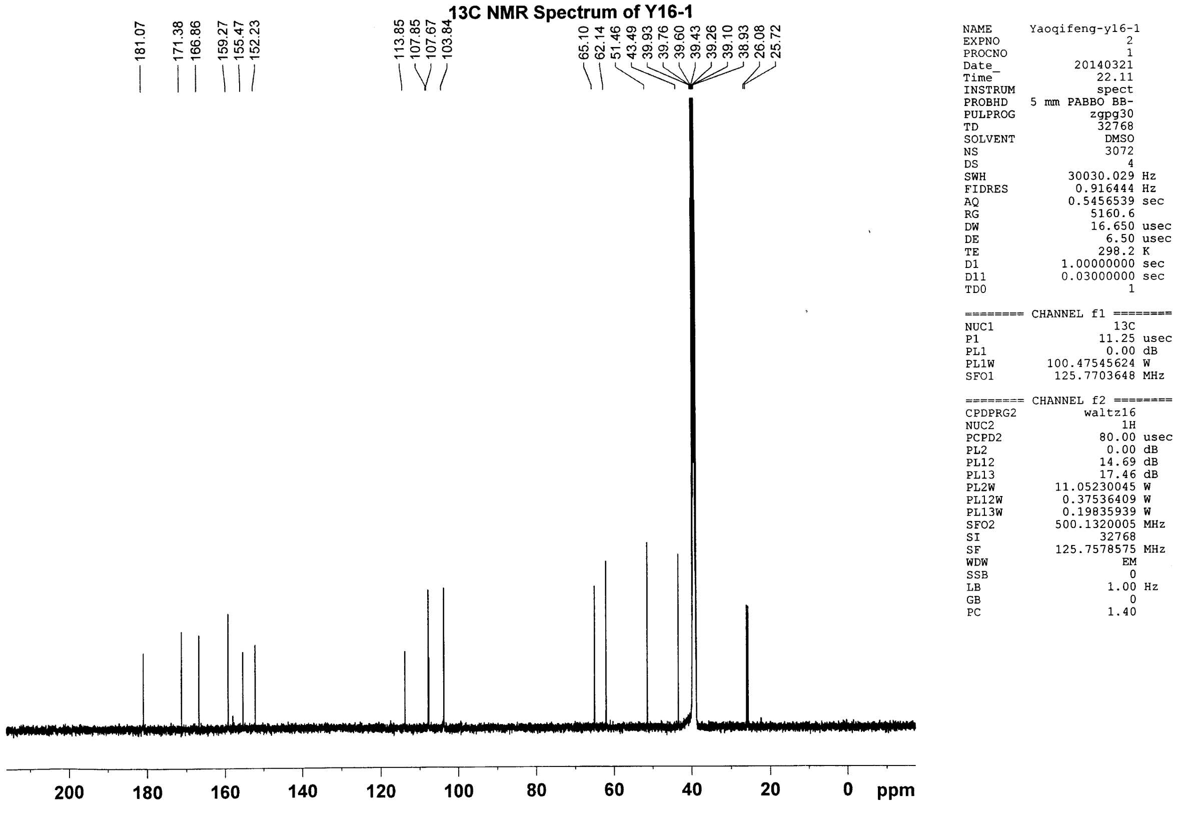

Figure S13. HSQC spectrum of **3**.

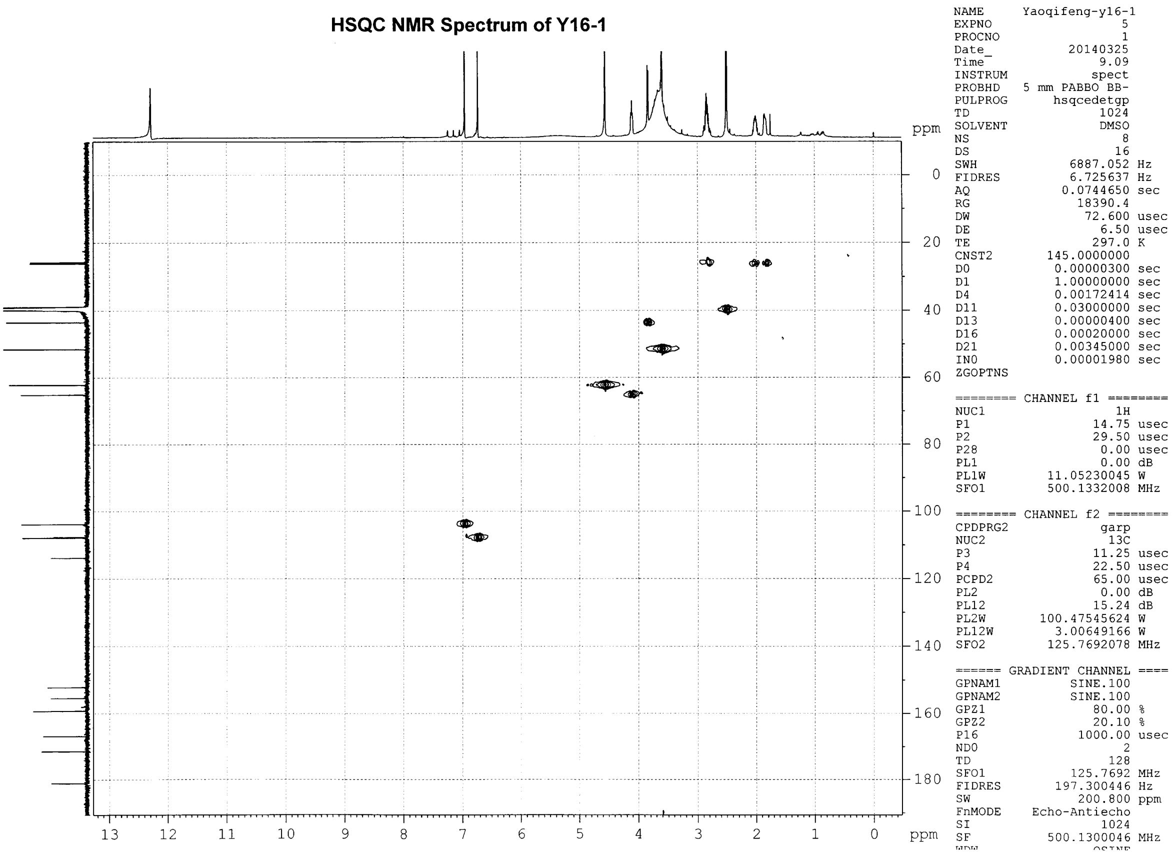

Figure S14. HMBC spectrum of **3**.

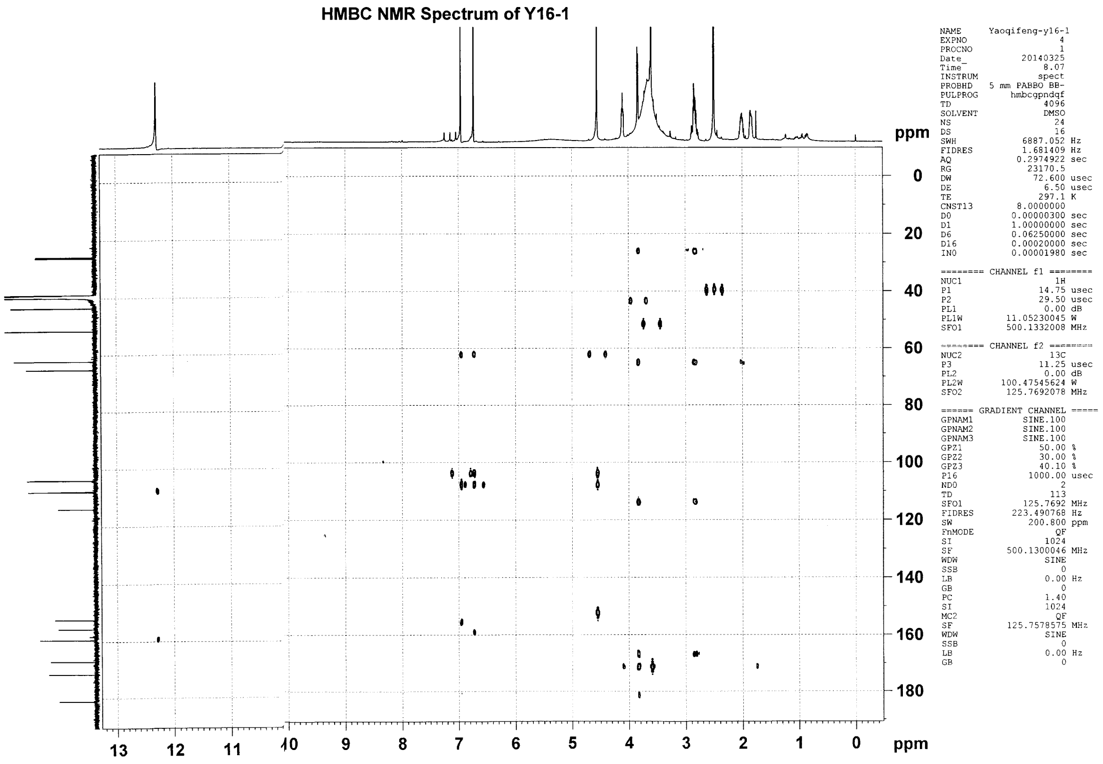

Figure S15. NOESY spectrum of **3**.

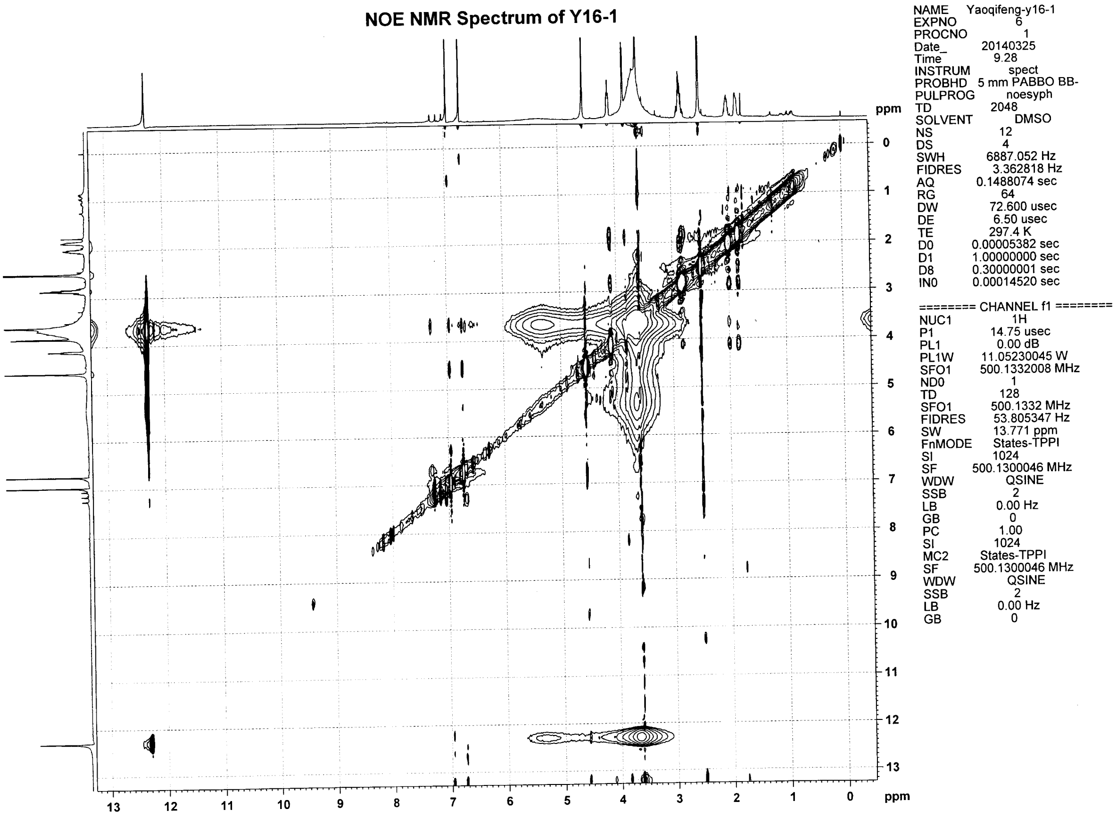

Figure S16. HRESIMS spectrum of **3**.

Mass Spectrum SmartFormula Report

Analysis Info		Acquisition Date	4/23/2014 3:07:54 PM	
Analysis Name	D:\Data\MS\data\201404\yaoqifeng-Y16-1_pos.d			
Method	POS_100-2000_Dirrect Infusion.m	Operator	SCSIO	
Sample Name	SCSIO	Instrument / Ser#	maXis	29
Comment				

Acquisition Parameter

Source Type	ESI	Ion Polarity	Positive	Set Nebulizer	0.4 Bar
Focus	Not active	Set Capillary	4500 V	Set Dry Heater	180 °C
Scan Begin	100 m/z	Set End Plate Offset	-500 V	Set Dry Gas	4.0 l/min
Scan End	2000 m/z	Set Collision Cell RF	2000.0 Vpp	Set Divert Valve	Waste

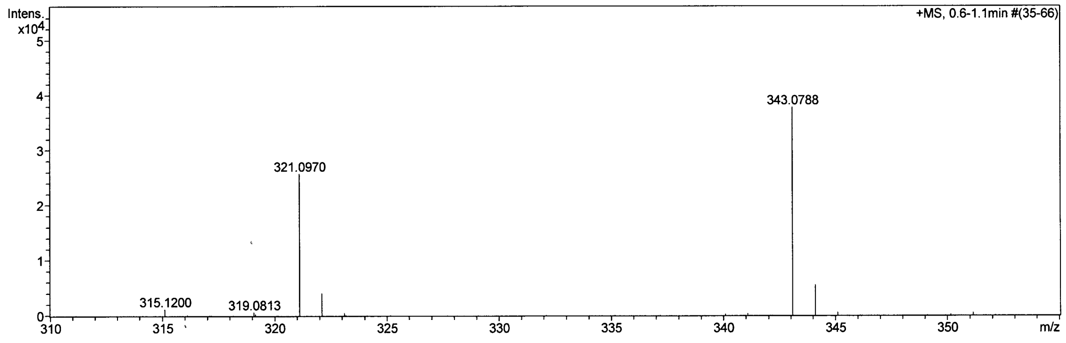

Meas. m/z	#	Formula	Score	m/z	err [mDa]	err [ppm]	mSigma	rdb	e⁻ Conf	N-Rule
321.0970	1	C 16 H 17 O 7	100.00	321.0969	-0.1	-0.2	11.1	8.5	even	ok
343.0788	1	C 16 H 16 Na O 7	100.00	343.0788	0.0	0.0	17.2	8.5	even	ok

Figure S17. IR spectrum of **3**.

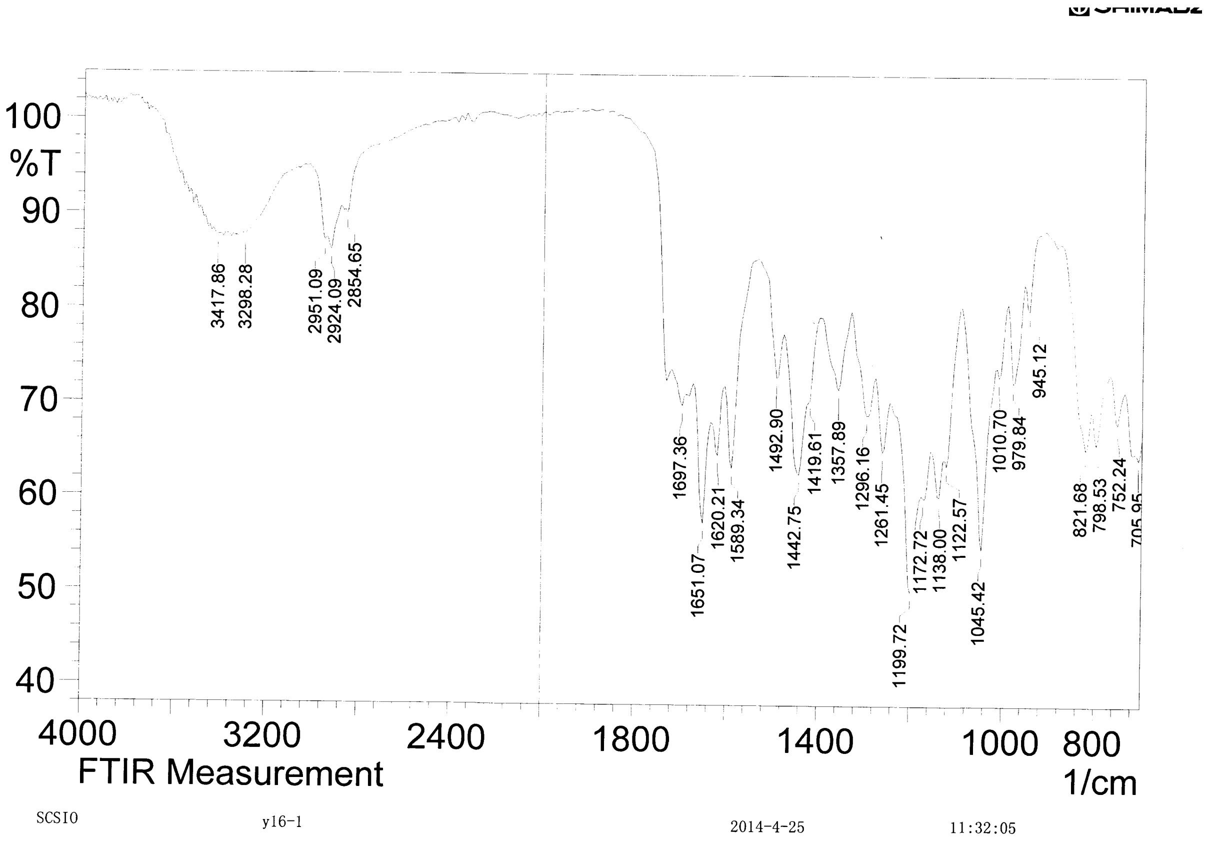

Figure S18. ^{1}H NMR spectrum of **4**.

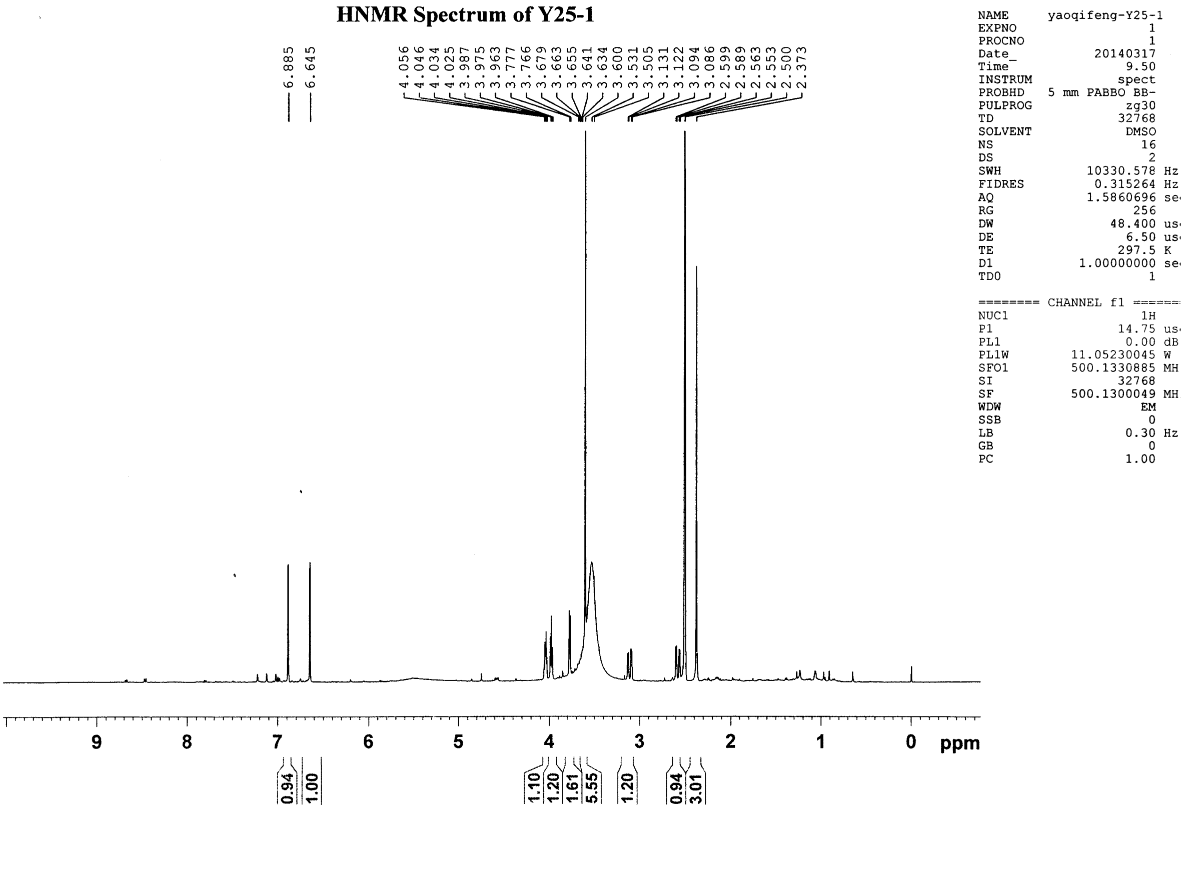

Figure S19. ^{13}C NMR spectrum of **4**.

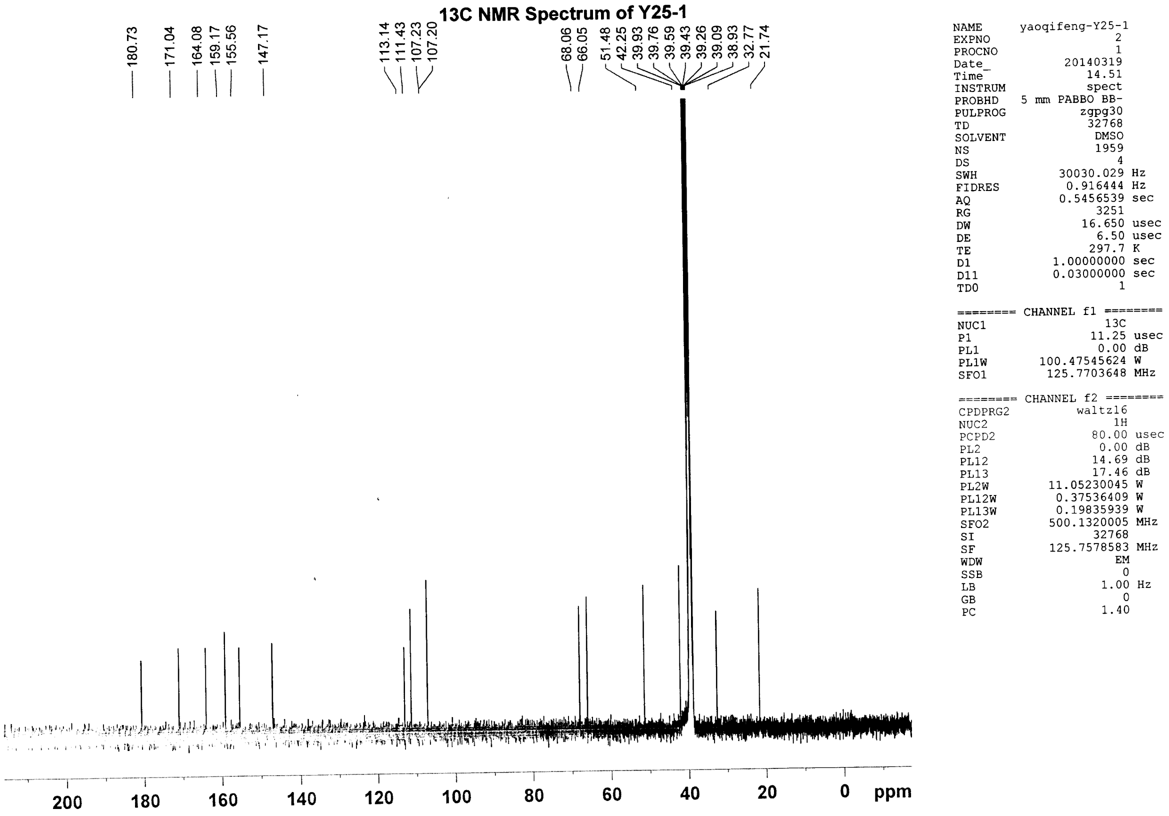

Figure S20. HSQC spectrum of **4**.

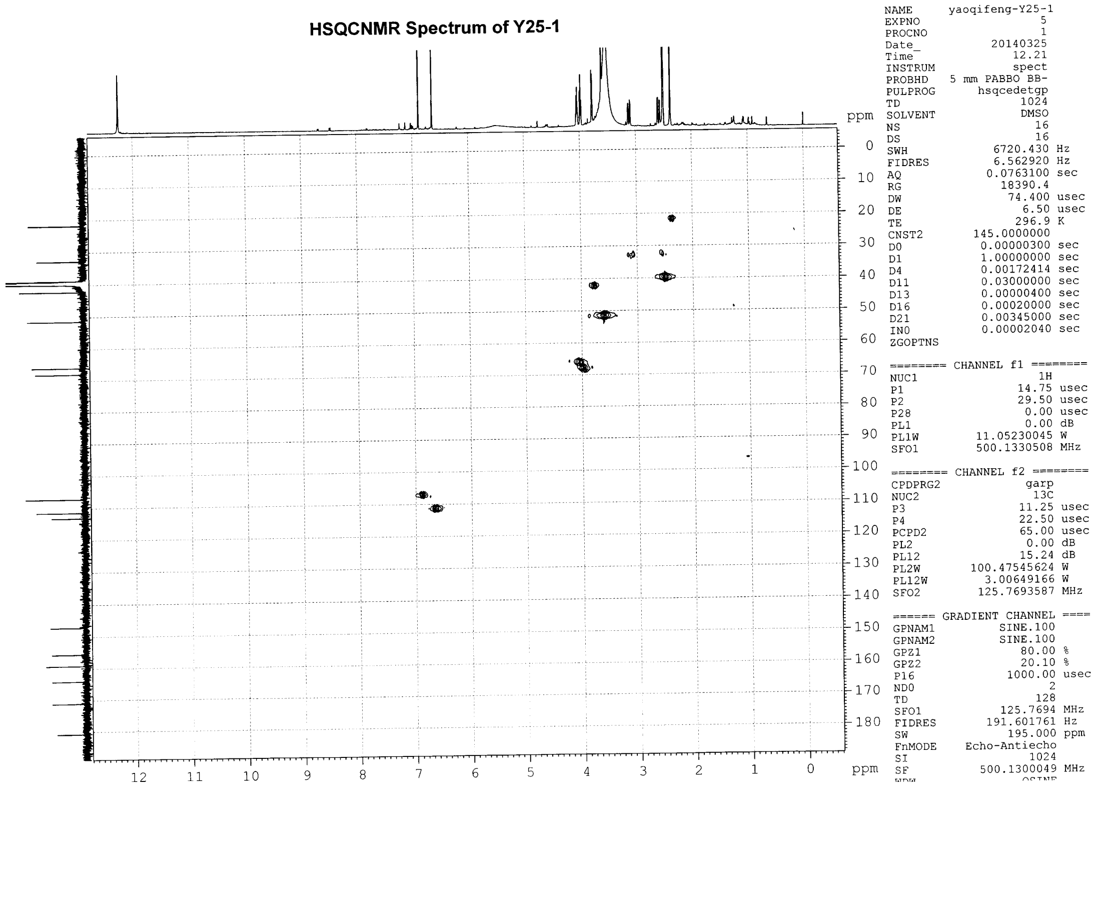

Figure S21. HMBC spectrum of **4**.

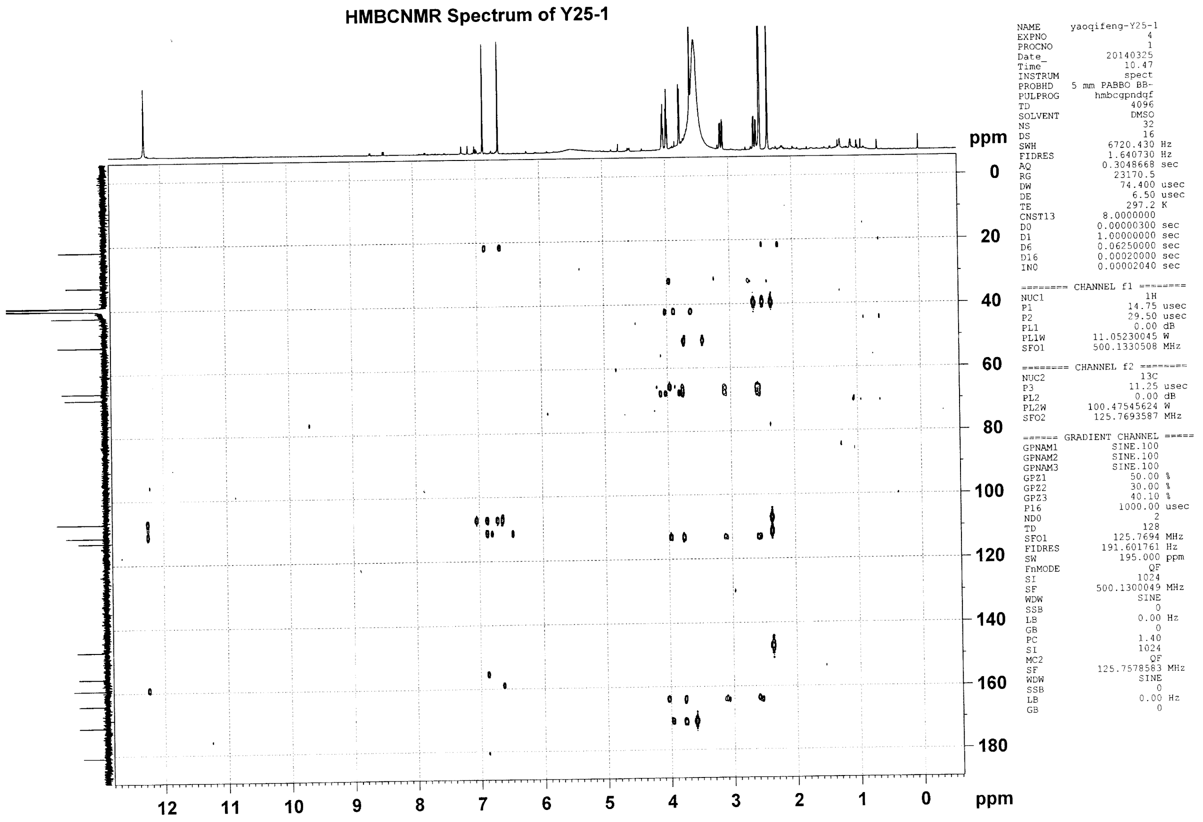

Figure S22. NOESY spectrum of **4**.

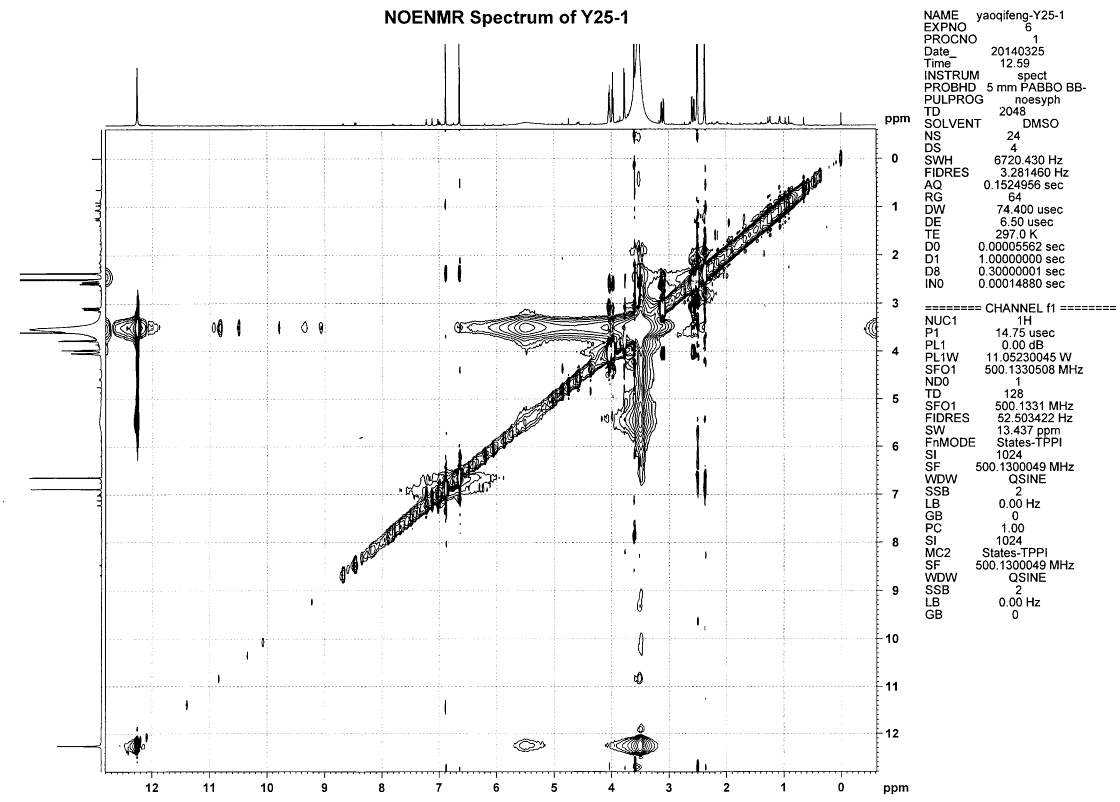

Figure S23. HRESIMS spectrum of **4**.

Mass Spectrum SmartFormula Report

**Analysis Info**		Acquisition Date	4/23/2014 3:25:15 PM
Analysis Name	D:\Data\MS\data\201404\yaoqifeng-Y25-1_pos.d		
Method	POS_100-2000_Dirrect Infusion.m	Operator	SCSIO
Sample Name	SCSIO	Instrument / Ser#	maXis 29
Comment			

Acquisition Parameter

Source Type	ESI	Ion Polarity	Positive	Set Nebulizer	0.4 Bar
Focus	Not active	Set Capillary	4500 V	Set Dry Heater	180 °C
Scan Begin	100 m/z	Set End Plate Offset	-500 V	Set Dry Gas	4.0 l/min
Scan End	2000 m/z	Set Collision Cell RF	2000.0 Vpp	Set Divert Valve	Waste

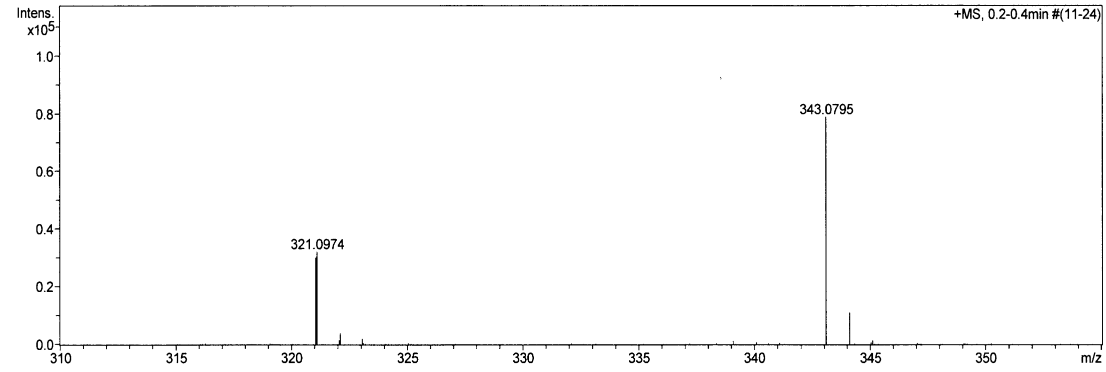

Meas. m/z	#	Formula	Score	m/z	err [mDa]	err [ppm]	mSigma	rdb	e⁻ Conf	N-Rule
321.0974	1	C 16 H 17 O 7	100.00	321.0969	-0.5	-1.6	30.1	8.5	even	ok
343.0795	1	C 16 H 16 Na O 7	100.00	343.0788	-0.7	-2.0	21.3	8.5	even	ok

Figure S24. IR spectrum of **4**.

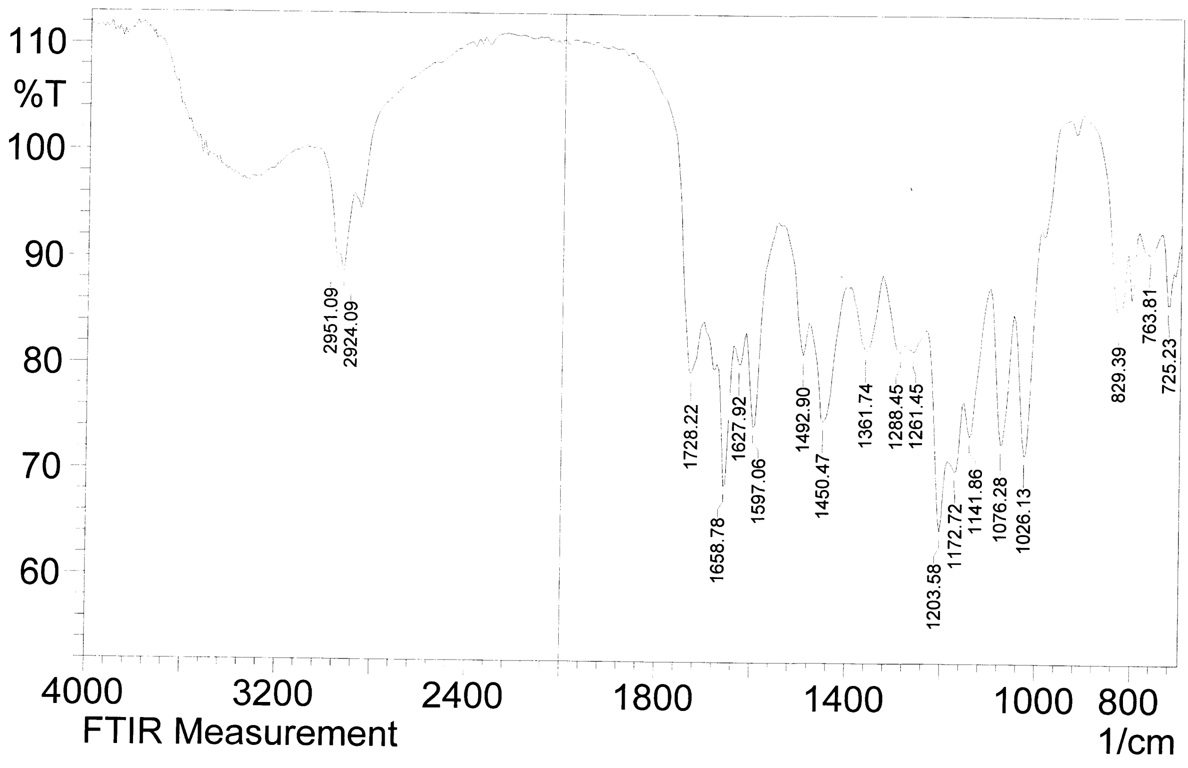

SCSIO y25-1 2014-4-25 11:27:26

Figure S25. ^{1}H NMR spectrum of **5**.

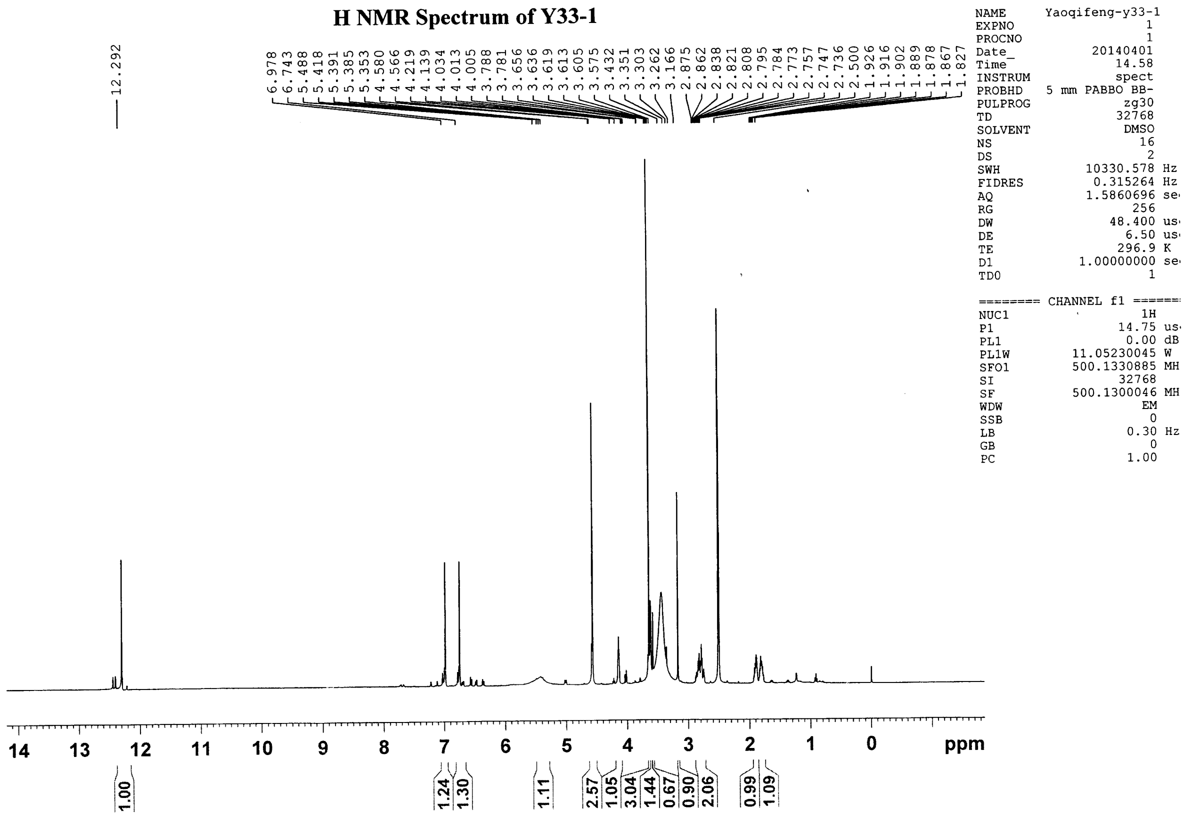

Figure S26. ^{13}C NMR spectrum of **5**.

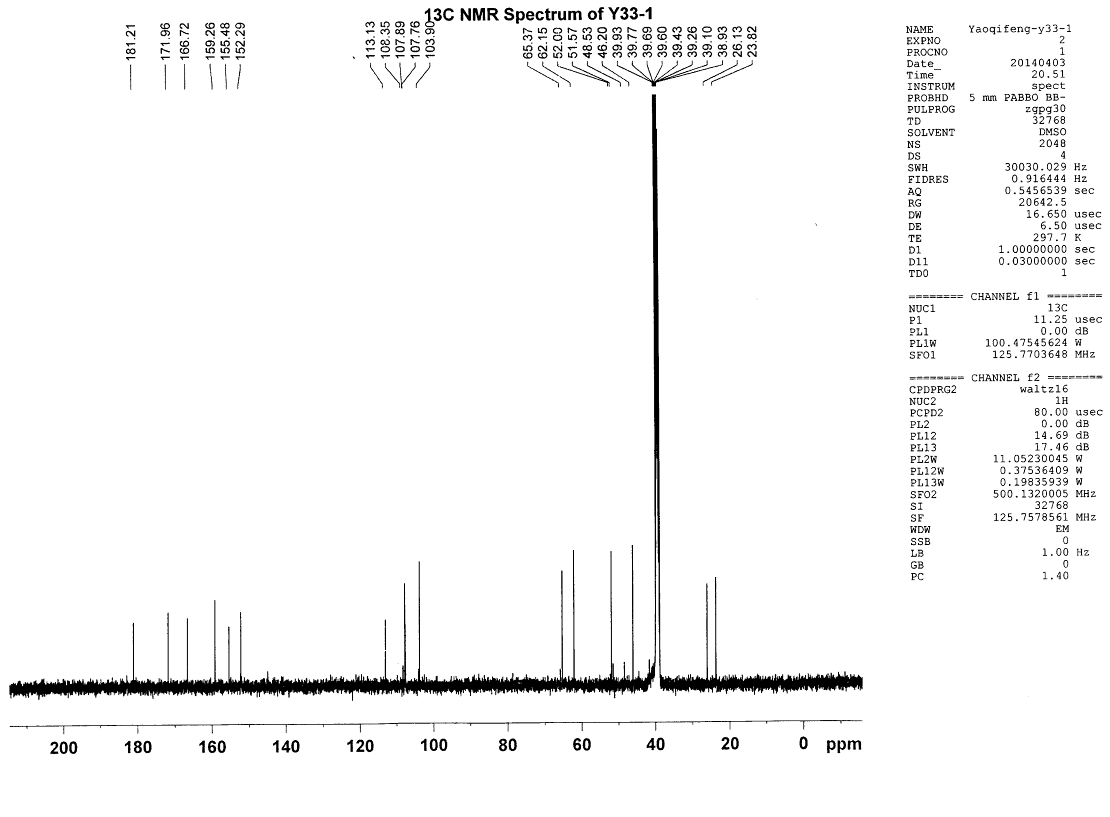

Figure S27. HMBC spectrum of **5**.

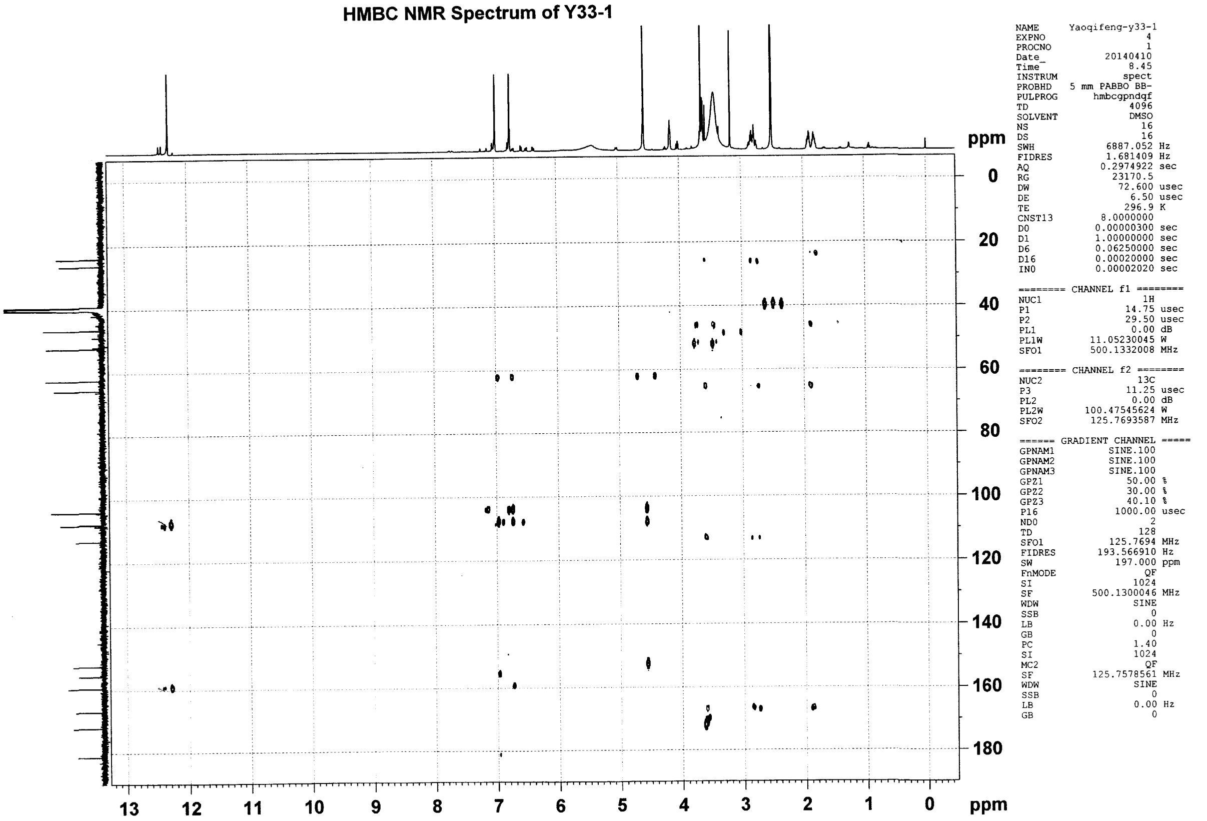

Figure S28. NOESY spectrum of **5**.

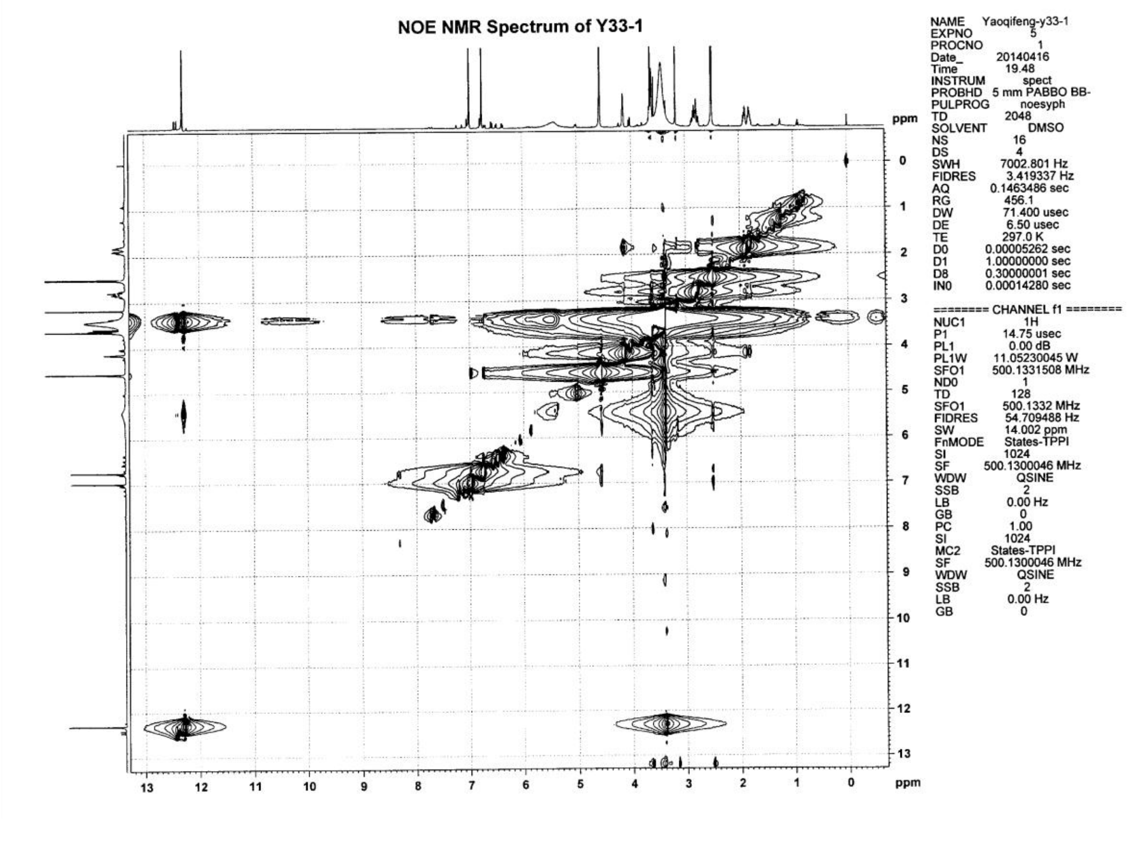

Figure S29. HRESIMS spectrum of **5**.

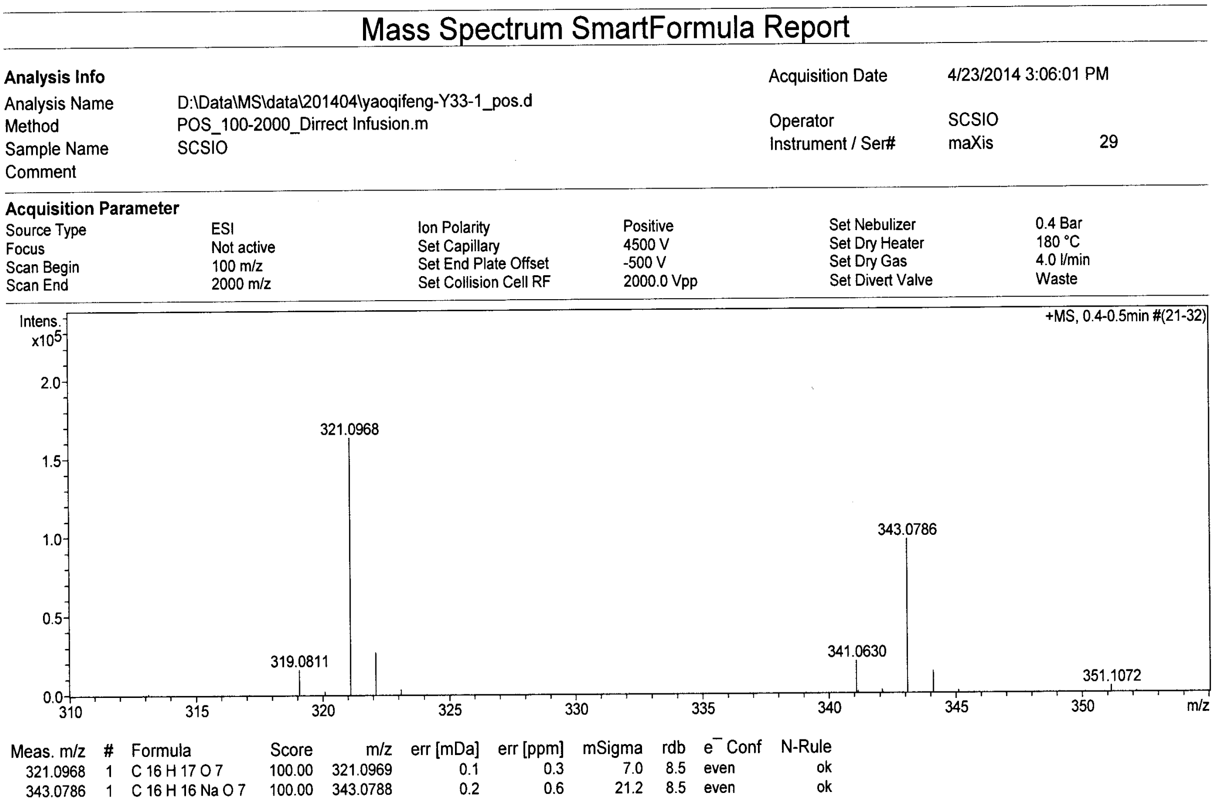

Figure S30. IR spectrum of **5**.

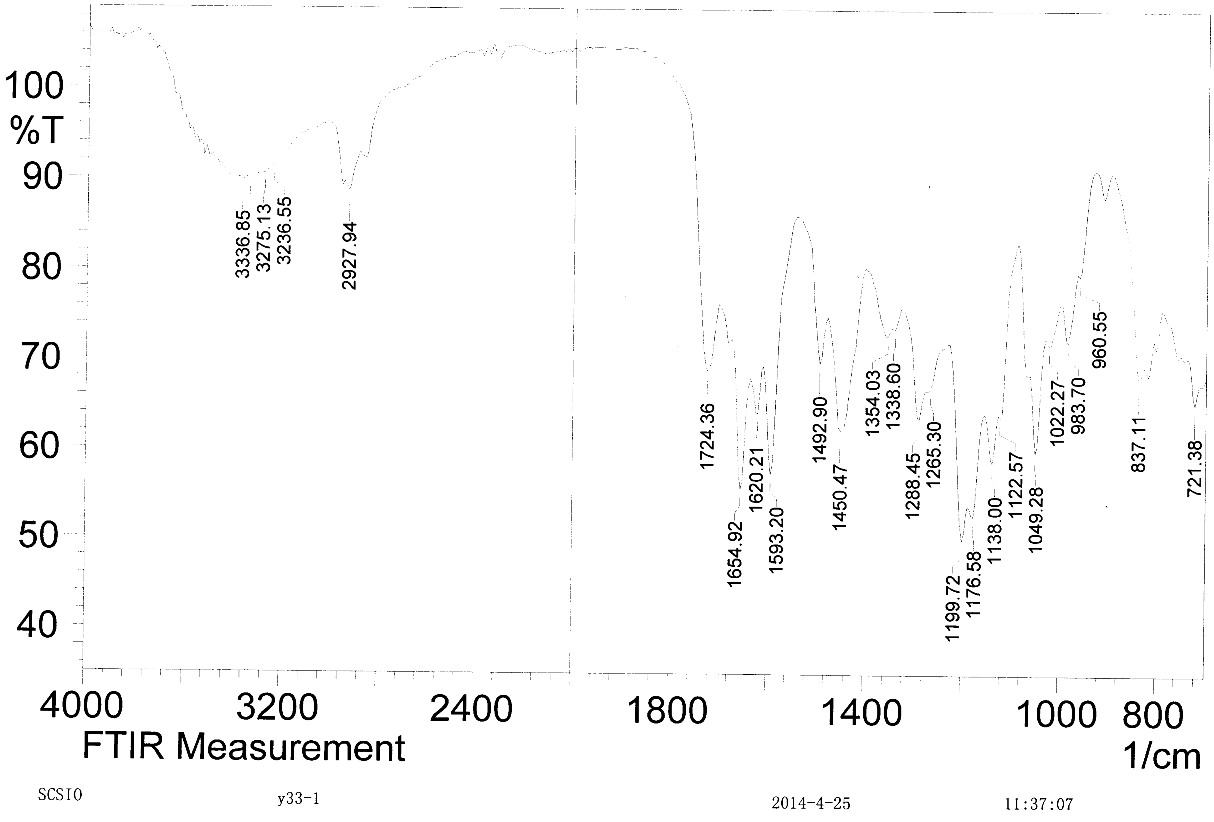

Figure S31. ^{1}H NMR spectrum of **6**.

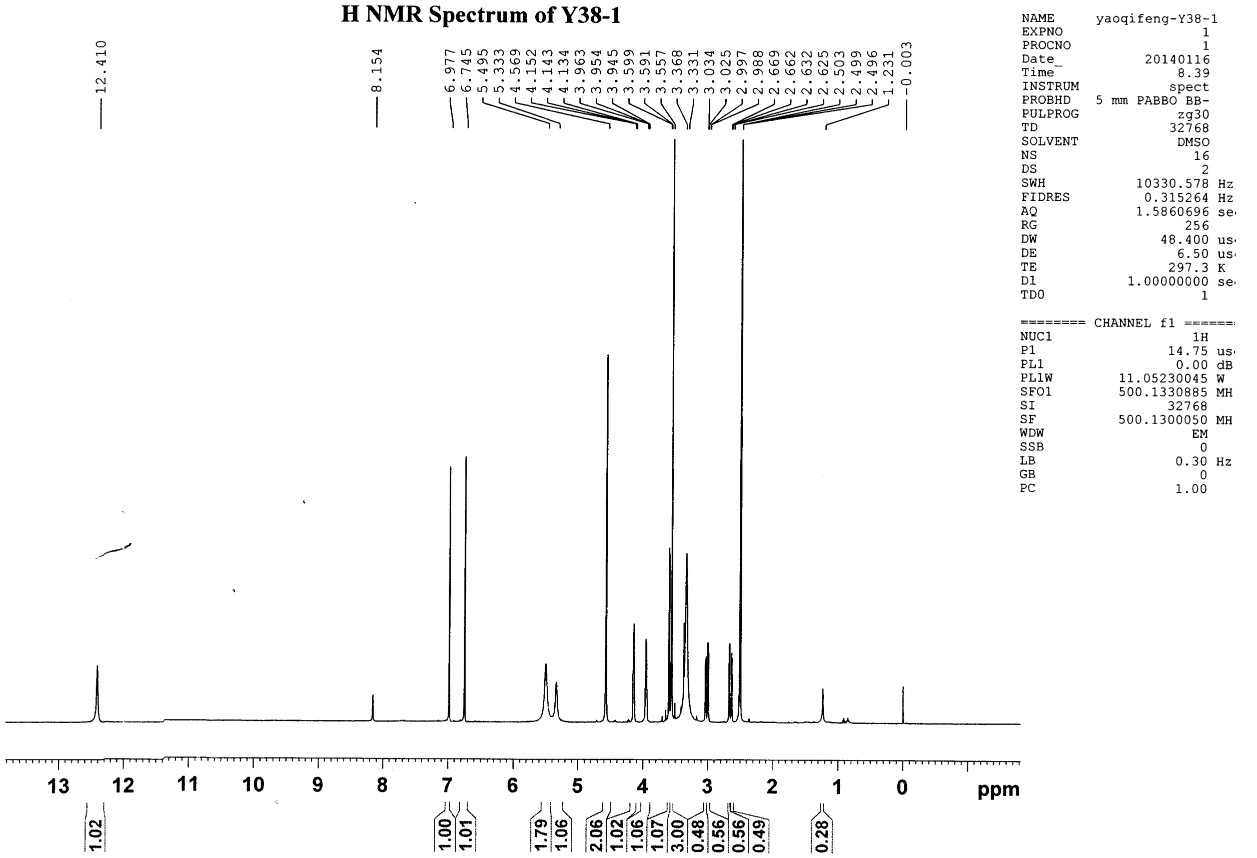

Figure S32. ^{13}C NMR spectrum of **6**.

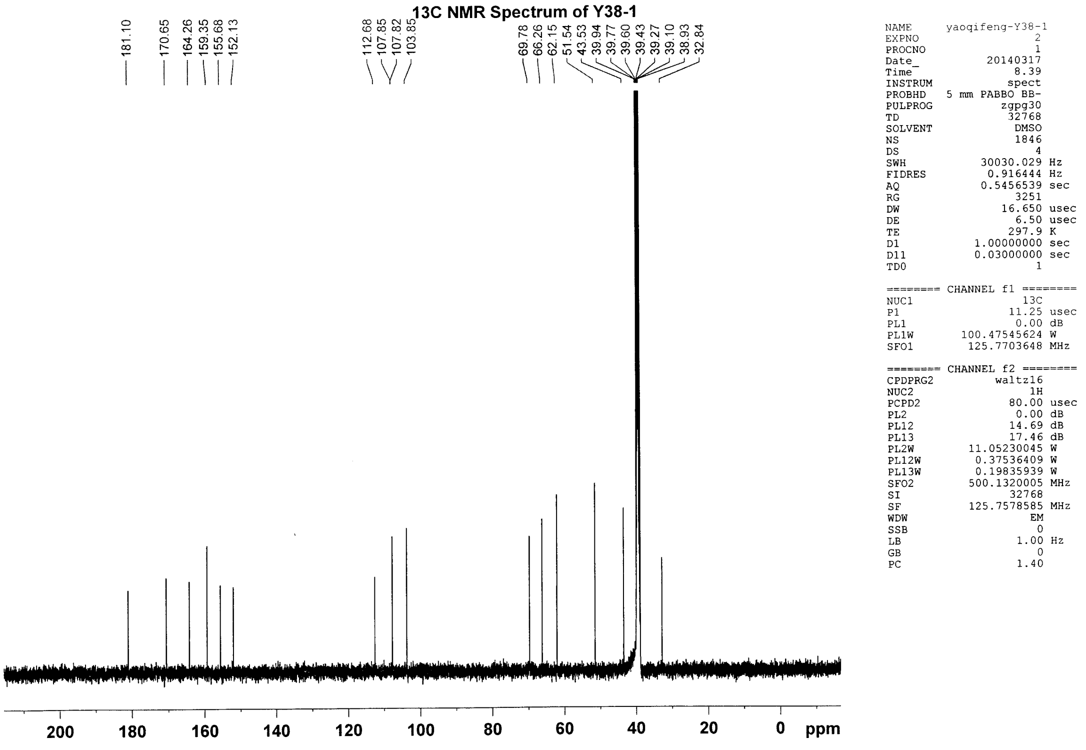

Figure S33. HSQC spectrum of **6**.

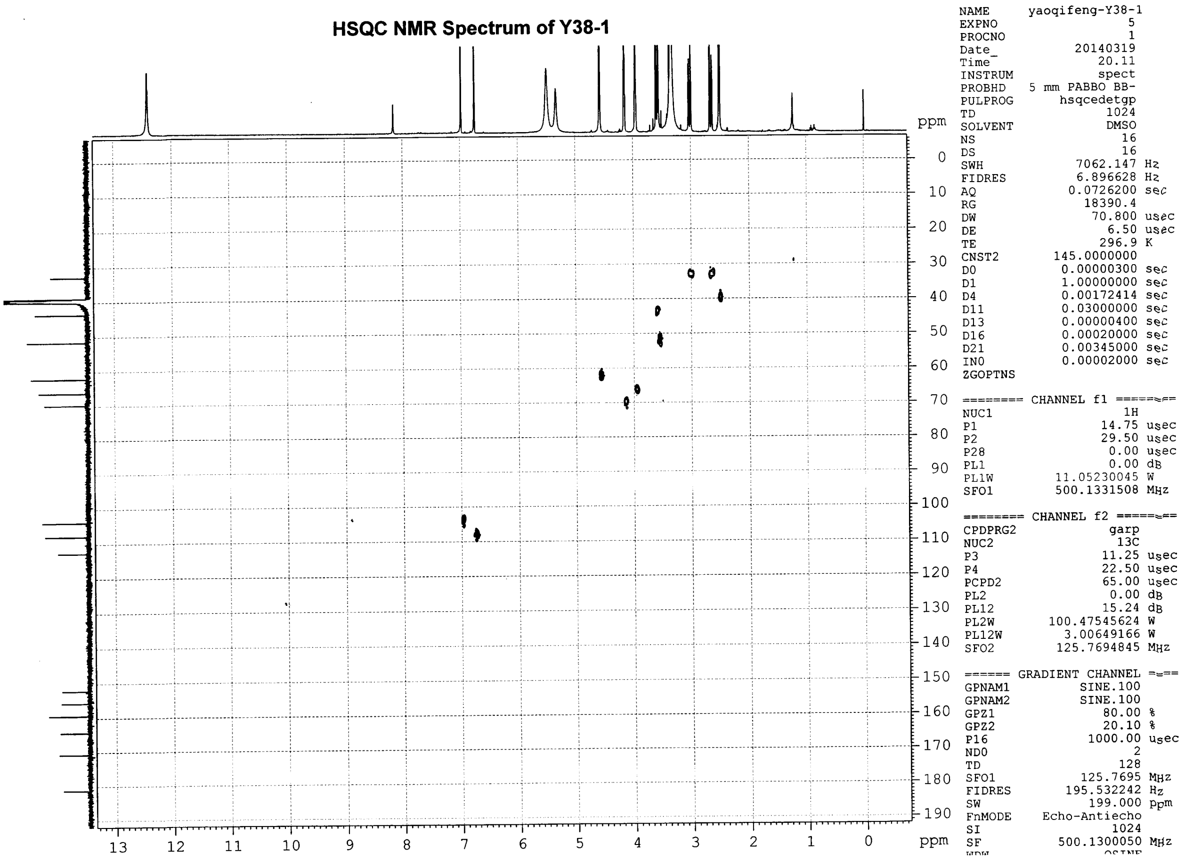

Figure S34. HMBC spectrum of **6**.

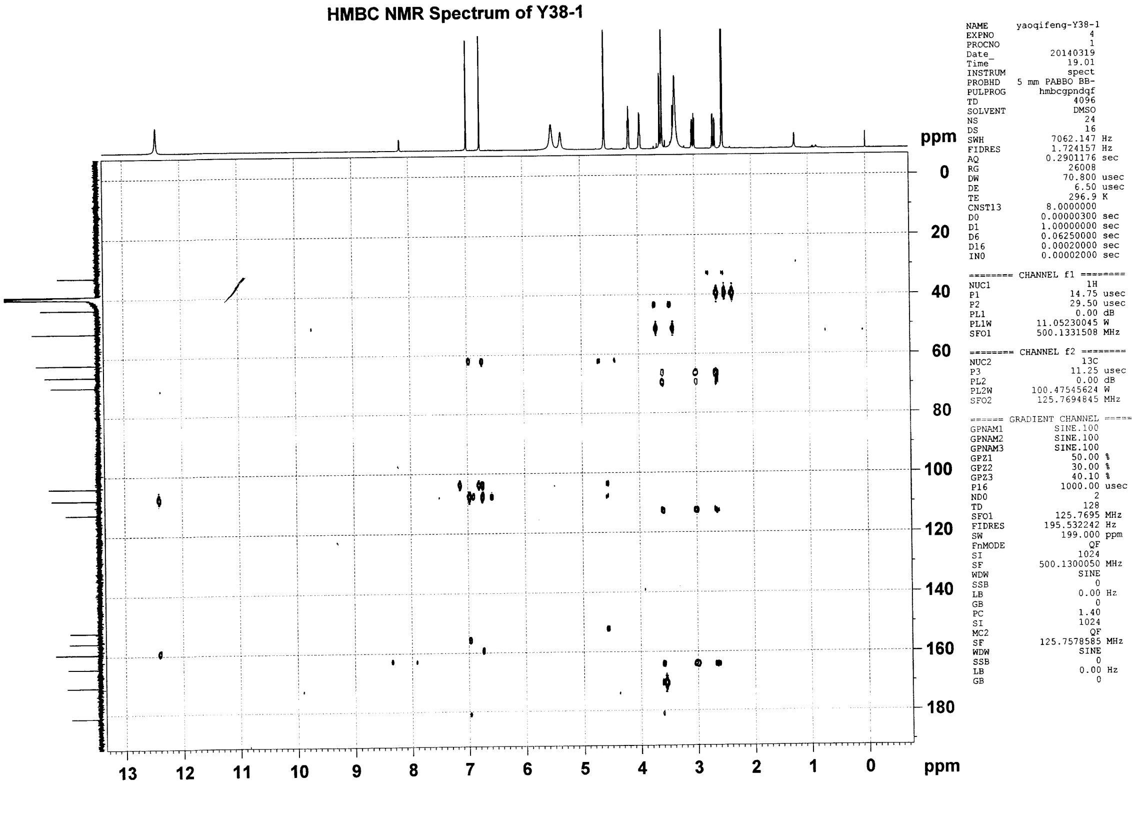

Figure S35. H-H COSY spectrum of **6**.

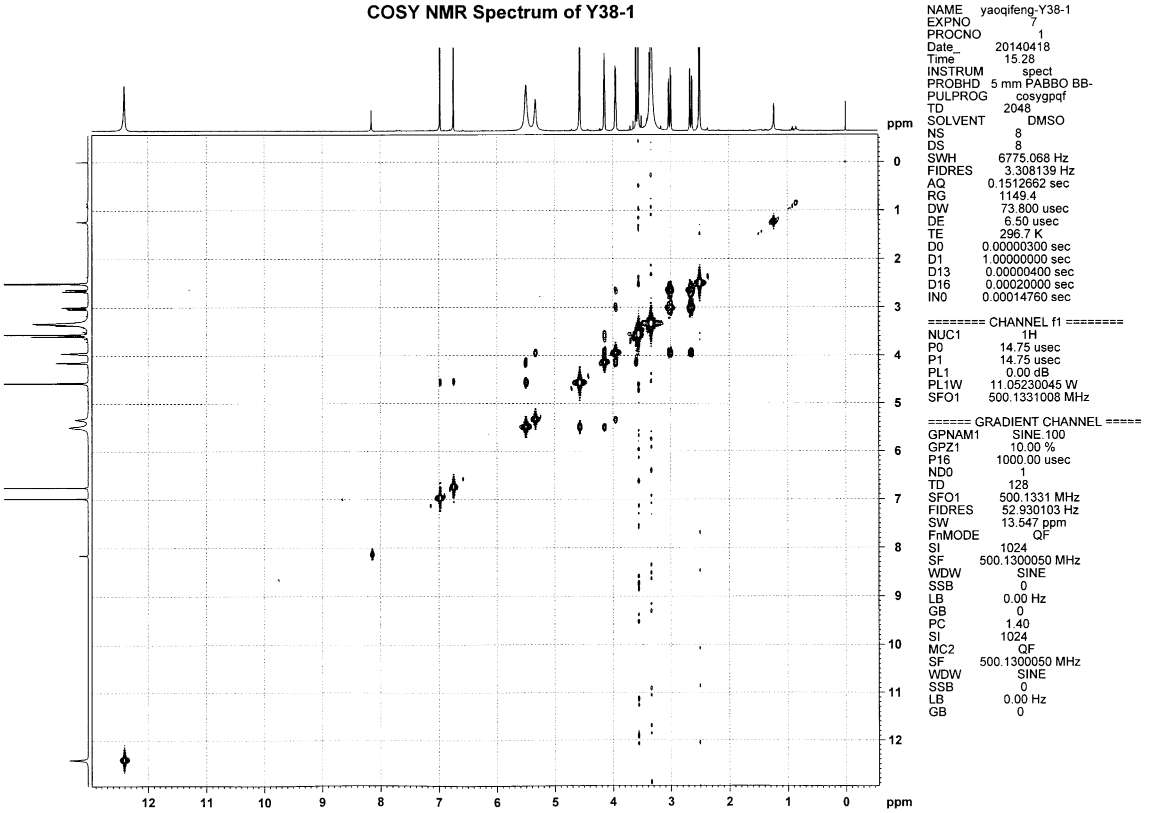

Figure S36. NOESY spectrum of **6**.

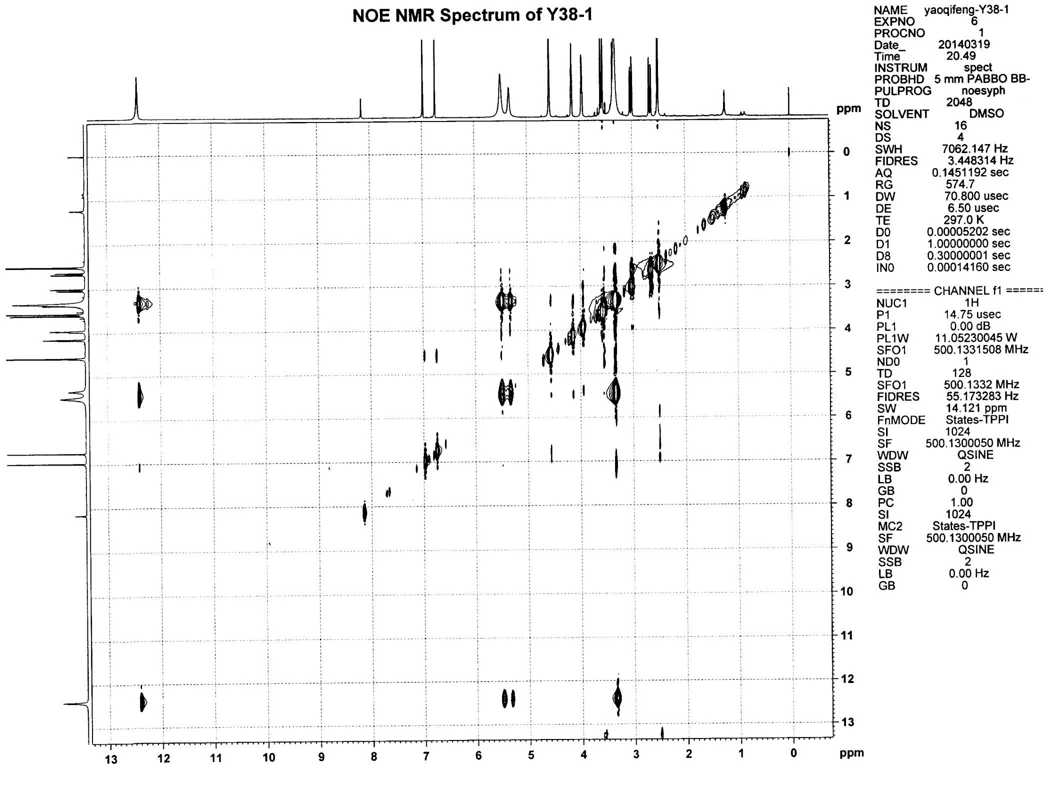

Figure S37. HRESIMS spectrum of **6**.

Mass Spectrum SmartFormula Report

Analysis Info

Analysis Name	D:\Data\MS\data\201404\yaoqifeng-Y38-1_pos.d	Acquisition Date	4/23/2014 3:10:23 PM
Method	POS_100-2000_Dirrect Infusion.m	Operator	SCSIO
Sample Name	SCSIO	Instrument / Ser#	maXis 29
Comment			

Acquisition Parameter

Source Type	ESI	Ion Polarity	Positive	Set Nebulizer	0.4 Bar
Focus	Not active	Set Capillary	4500 V	Set Dry Heater	180 °C
Scan Begin	100 m/z	Set End Plate Offset	-500 V	Set Dry Gas	4.0 l/min
Scan End	2000 m/z	Set Collision Cell RF	2000.0 Vpp	Set Divert Valve	Waste

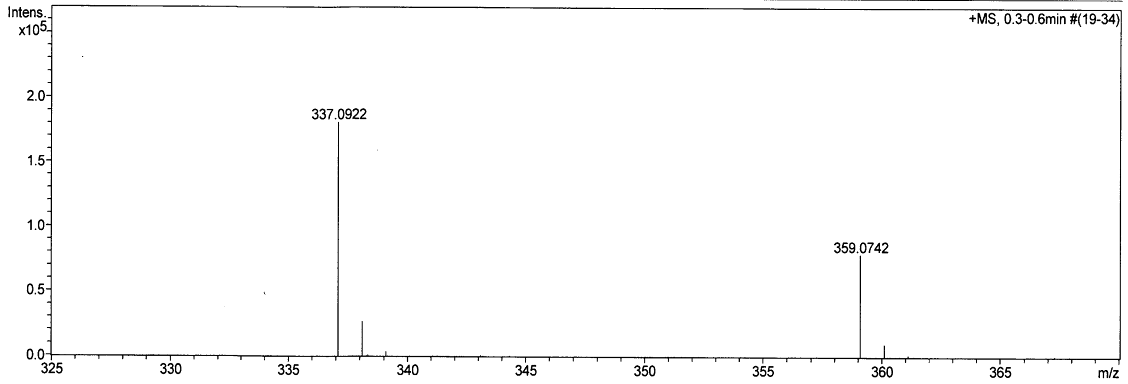

Meas. m/z	#	Formula	Score	m/z	err [mDa]	err [ppm]	mSigma	rdb	e⁻ Conf	N-Rule
337.0922	1	C 16 H 17 O 8	100.00	337.0918	-0.4	-1.3	16.4	8.5	even	ok
359.0742	1	C 16 H 16 Na O 8	100.00	359.0737	-0.4	-1.2	30.2	8.5	even	ok

Figure S38. HRESIMS spectrum of **6**.

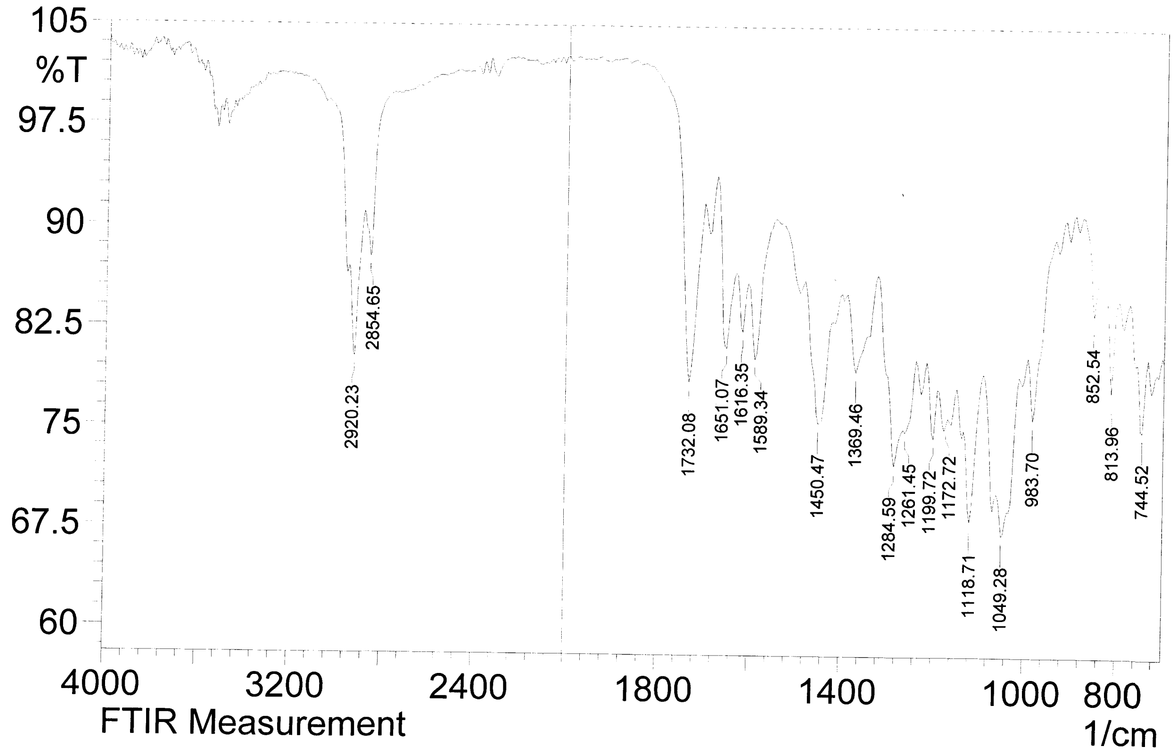

SCSIO y38-1 2014-4-25 11:41:42

Figure S39. ^{1}H NMR spectrum of **7**.

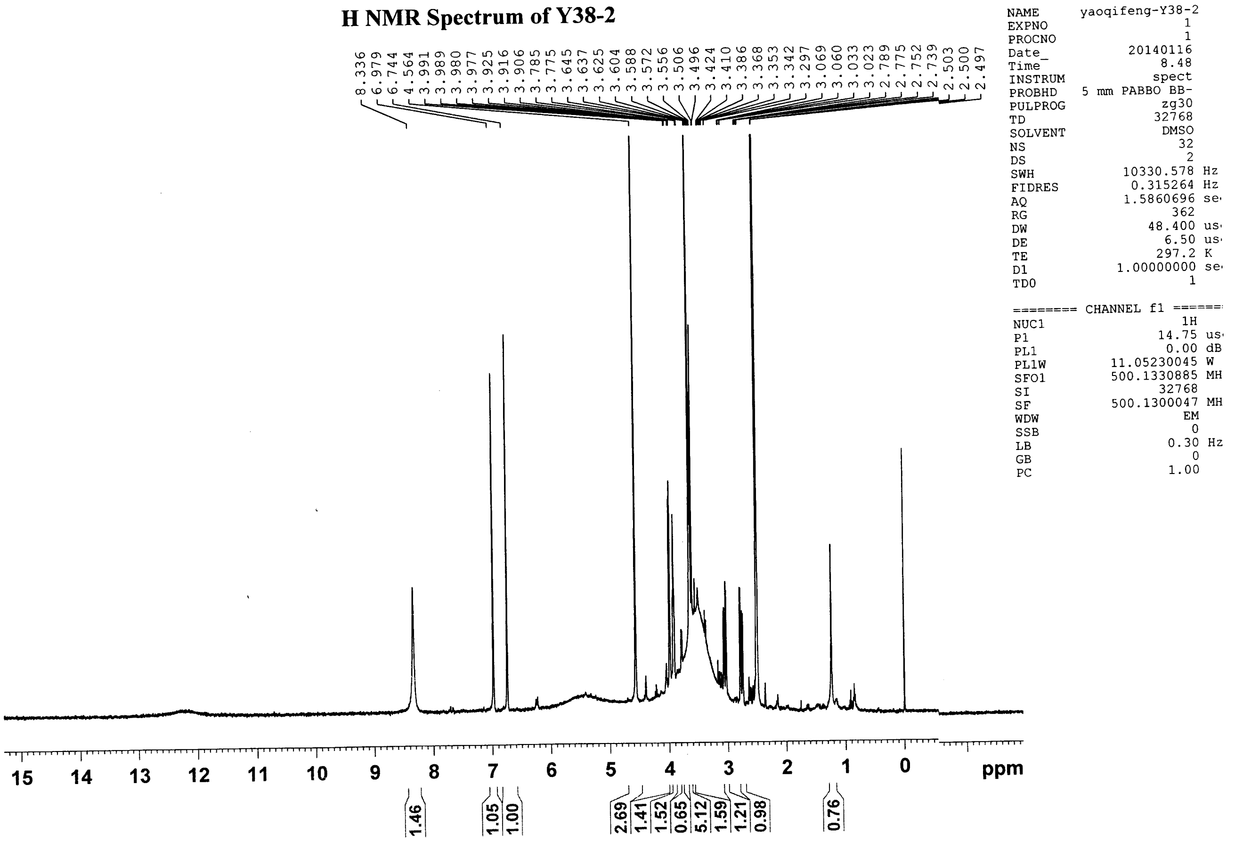

Figure S40. ^{13}C NMR spectrum of **7**.

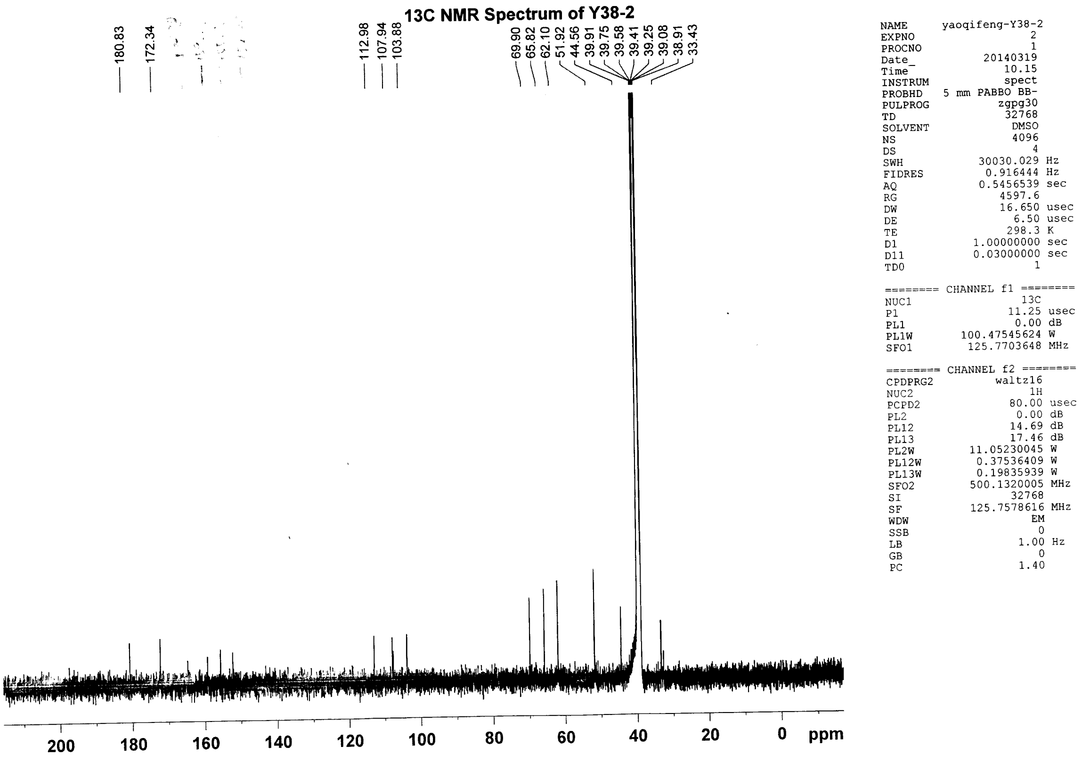

Figure S41. HSQC spectrum of **7**.

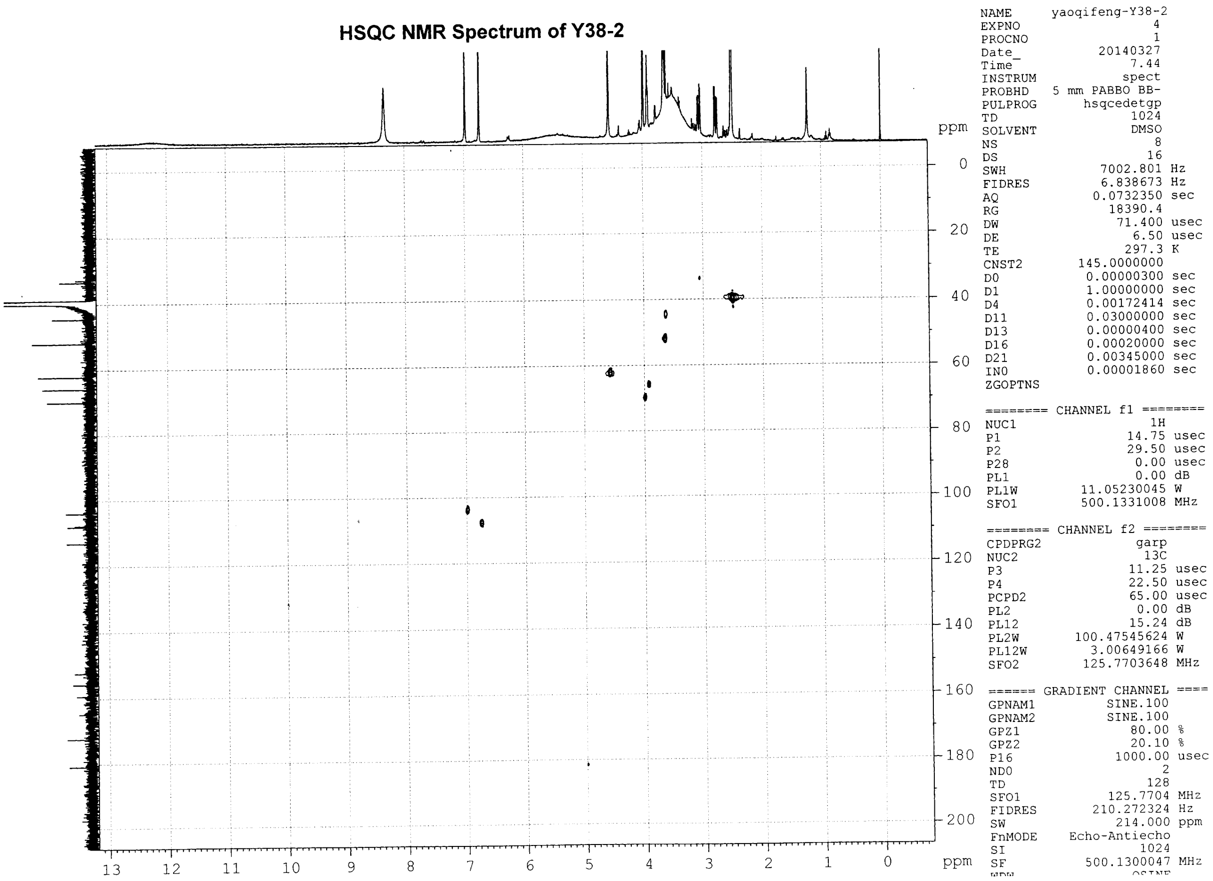

Figure S42. HMBC spectrum of **7**.

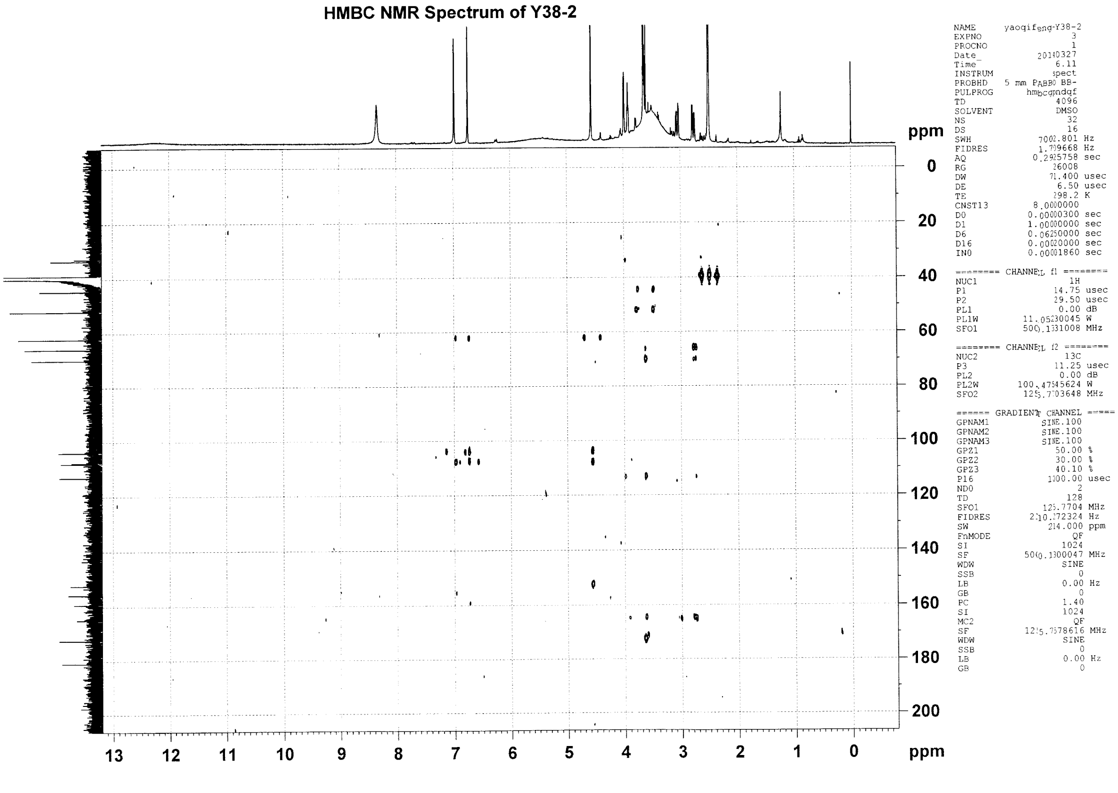

Figure S43. NOESY spectrum of **7**.

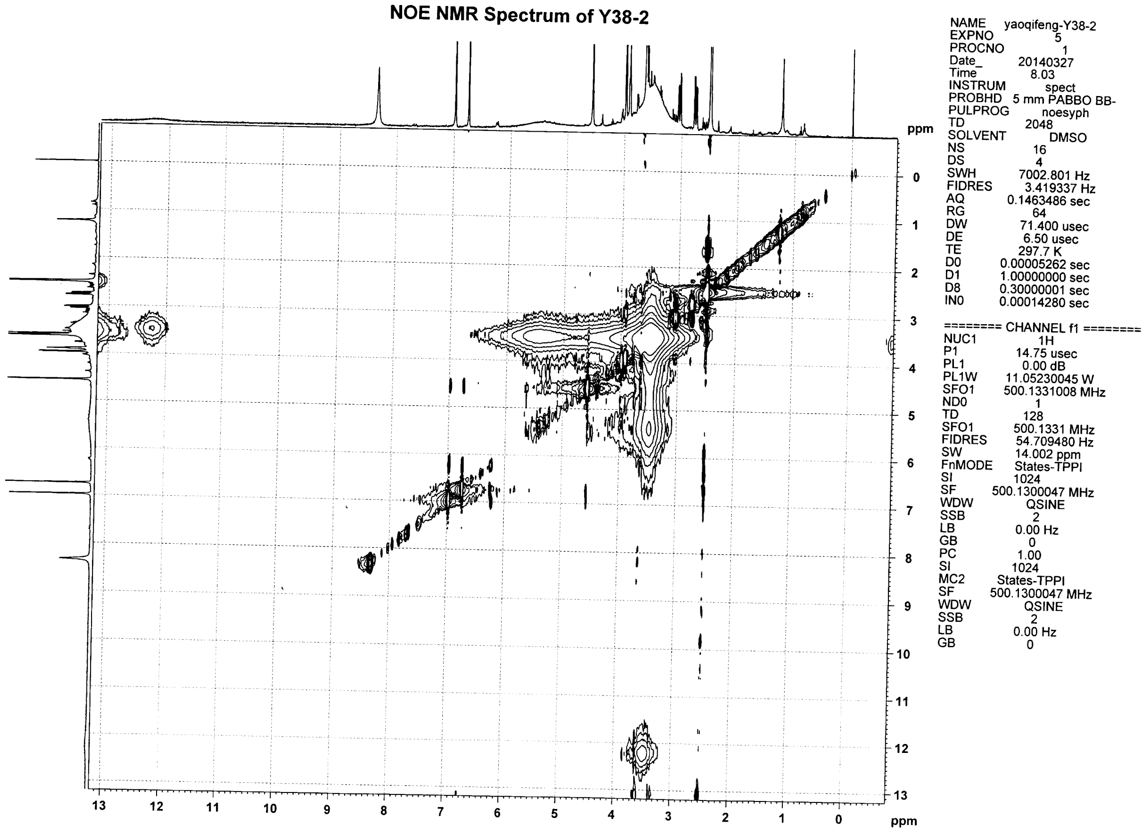

Figure S44. HRESIMS spectrum of **7**.

Mass Spectrum SmartFormula Report

Analysis Info

Analysis Name	D:\Data\MS\data\201404\yaoqifeng-Y38-2_pos.d	Acquisition Date	4/23/2014 3:32:12 PM
Method	POS_100-2000_Dirrect Infusion.m	Operator	SCSIO
Sample Name	SCSIO	Instrument / Ser#	maXis 29
Comment			

Acquisition Parameter

Source Type	ESI	Ion Polarity	Positive	Set Nebulizer	0.4 Bar
Focus	Not active	Set Capillary	4500 V	Set Dry Heater	180 °C
Scan Begin	100 m/z	Set End Plate Offset	-500 V	Set Dry Gas	4.0 l/min
Scan End	2000 m/z	Set Collision Cell RF	2000.0 Vpp	Set Divert Valve	Waste

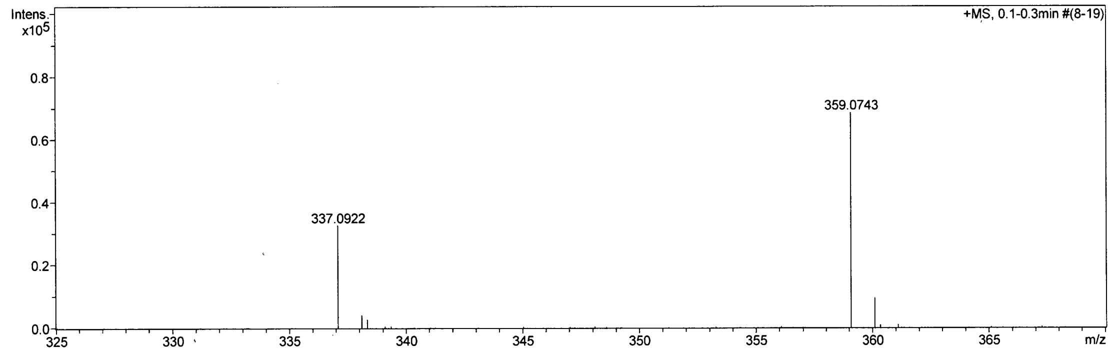

Meas. m/z	#	Formula	Score	m/z	err [mDa]	err [ppm]	mSigma	rdb	e⁻ Conf	N-Rule
337.0922	1	C 16 H 17 O 8	100.00	337.0918	-0.4	-1.2	27.8	8.5	even	ok
359.0743	1	C 16 H 16 Na O 8	100.00	359.0737	-0.5	-1.5	22.2	8.5	even	ok

Figure S45. IR spectrum of **7**.

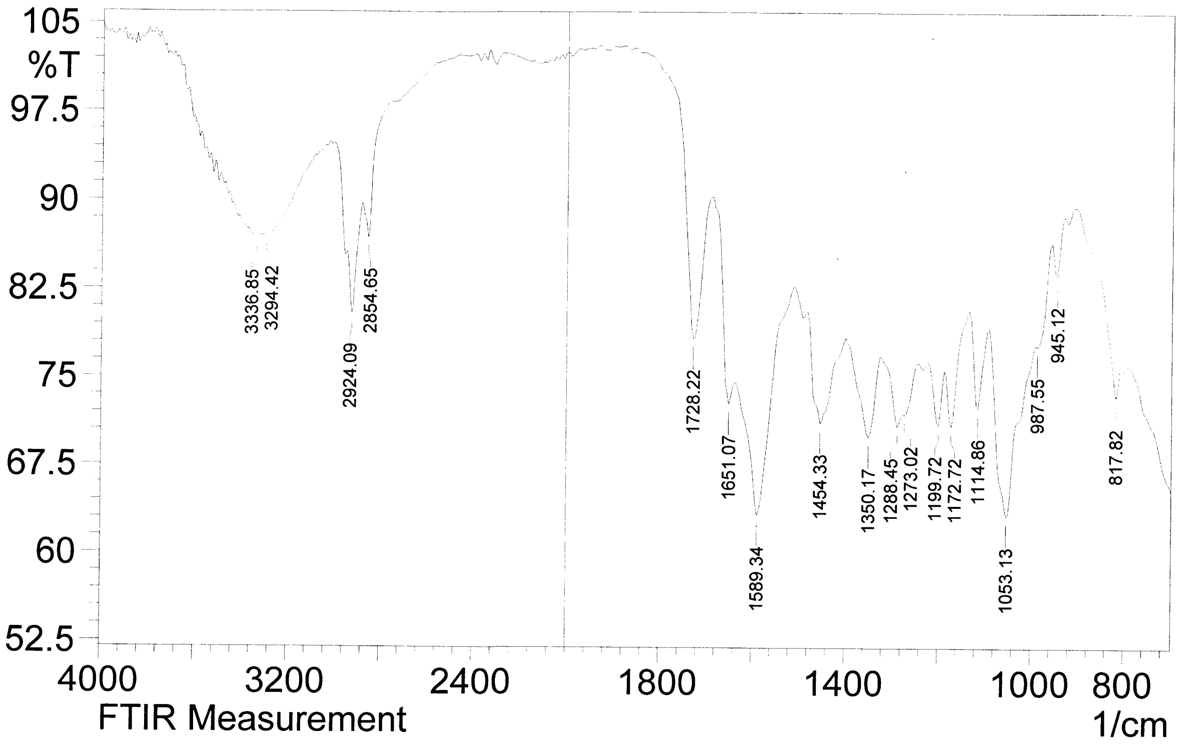

Figure S46. ^{1}H NMR spectrum of **8**.

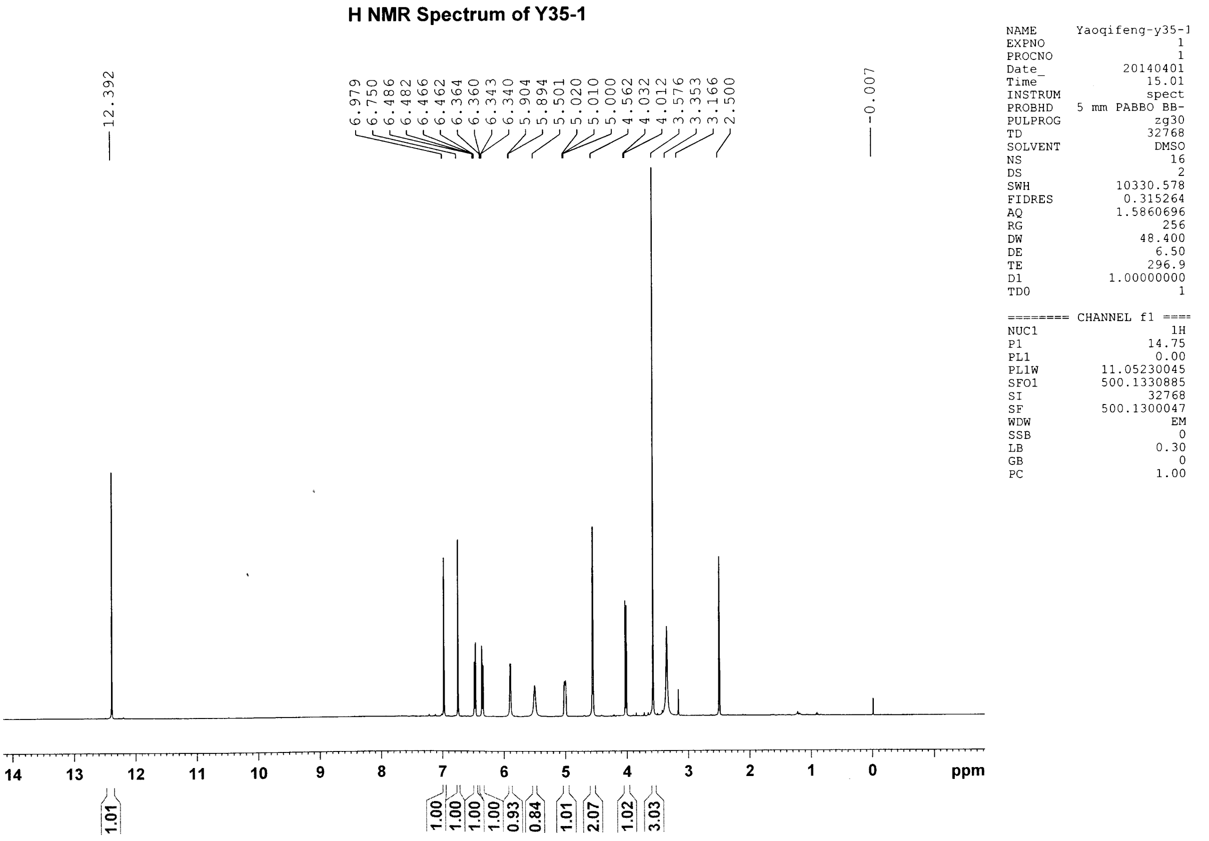

Figure S47. ^{13}C NMR spectrum of **8**.

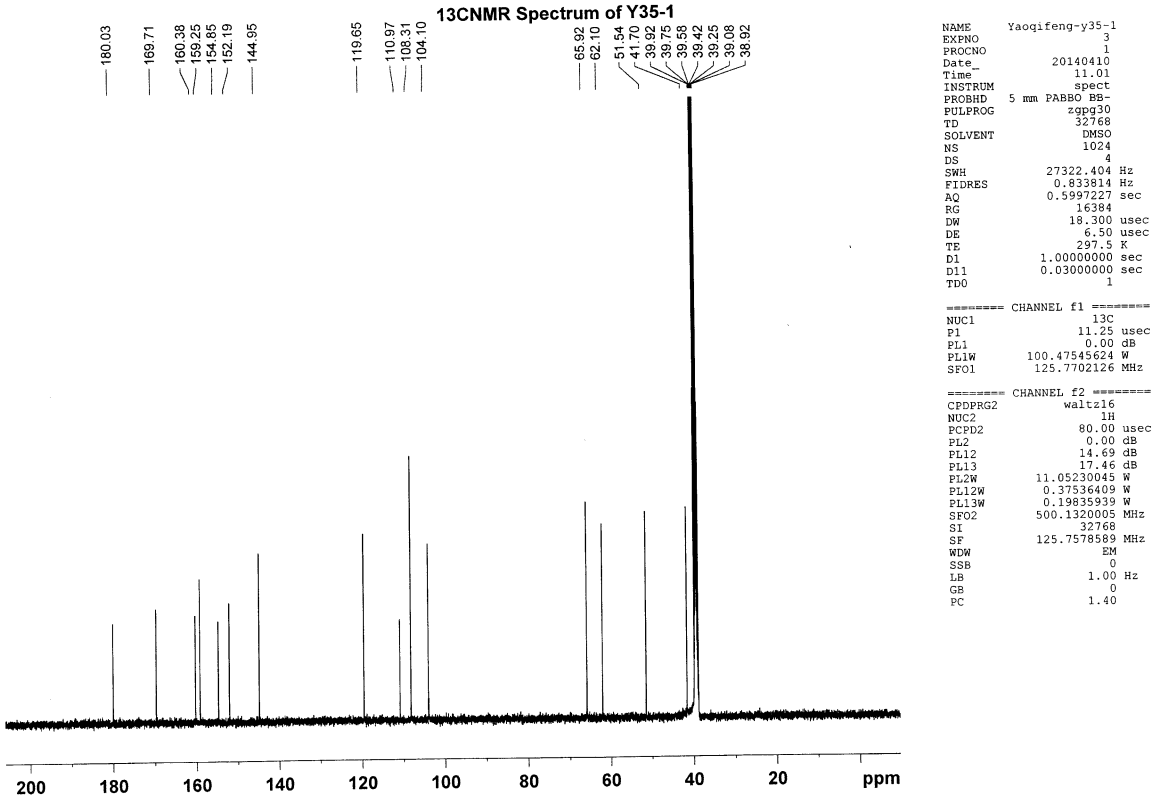

Figure S48. HMBC spectrum of **8**.

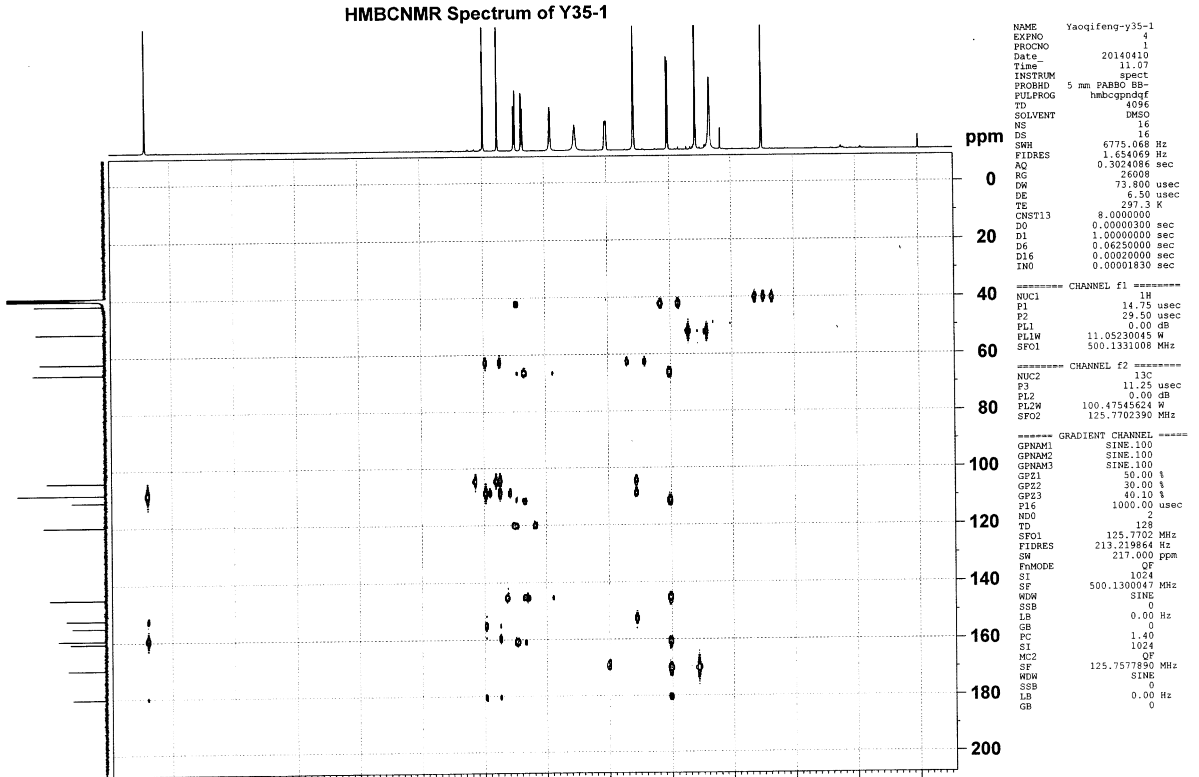

Figure S49. NOESY spectrum of **8**.

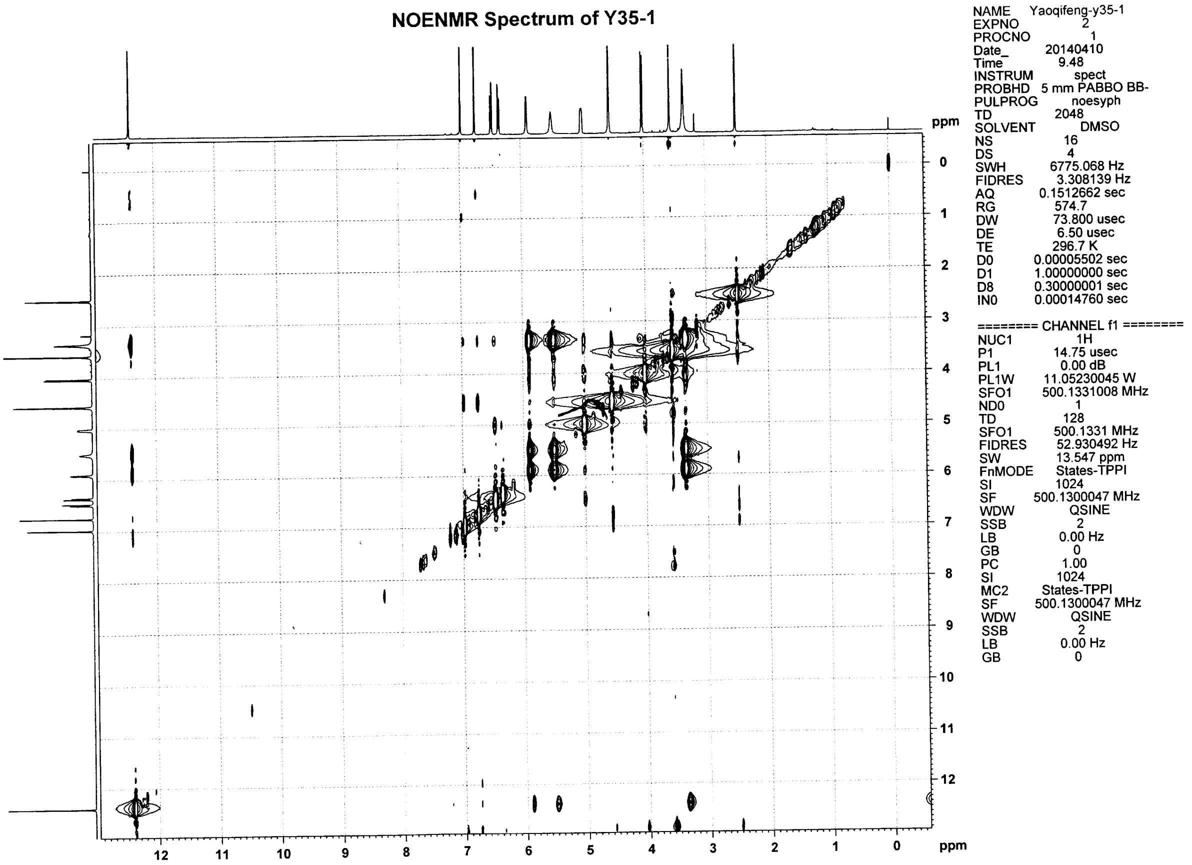

Figure S50. HRESIMS spectrum of **8**.

Mass Spectrum SmartFormula Report

Analysis Info

Analysis Name	D:\Data\MS\data\201404\yaoqifeng-Y35-1_pos.d	Acquisition Date	4/23/2014 3:22:03 PM
Method	POS_100-2000_Dirrect Infusion.m	Operator	SCSIO
Sample Name	SCSIO	Instrument / Ser#	maXis 29
Comment			

Acquisition Parameter

Source Type	ESI	Ion Polarity	Positive	Set Nebulizer	0.4 Bar
Focus	Not active	Set Capillary	4500 V	Set Dry Heater	180 °C
Scan Begin	100 m/z	Set End Plate Offset	-500 V	Set Dry Gas	4.0 l/min
Scan End	2000 m/z	Set Collision Cell RF	2000.0 Vpp	Set Divert Valve	Waste

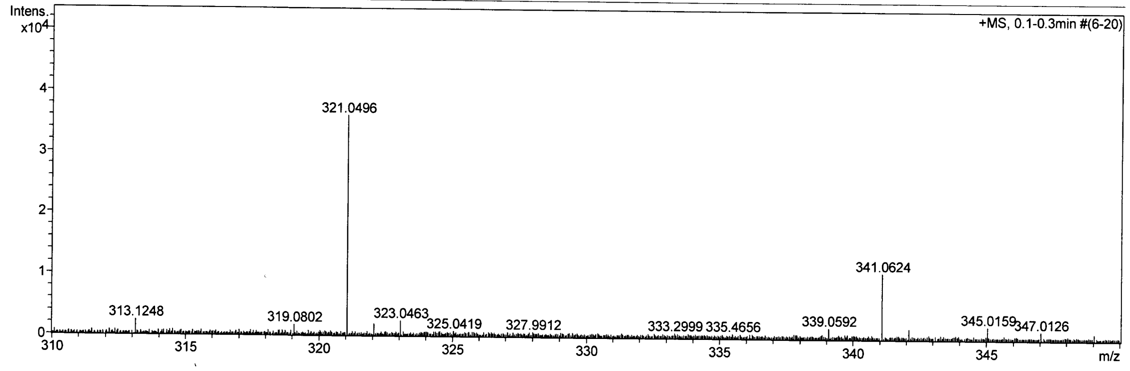

Meas. m/z	#	Formula	Score	m/z	err [mDa]	err [ppm]	mSigma	rdb	e⁻ Conf	N-Rule
319.0802	1	C 16 H 15 O 7	100.00	319.0812	1.0	3.3	74.8	9.5	even	ok
341.0624	1	C 16 H 14 Na O 7	100.00	341.0632	0.7	2.1	20.0	9.5	even	ok

Figure S51. IR spectrum of **8**.

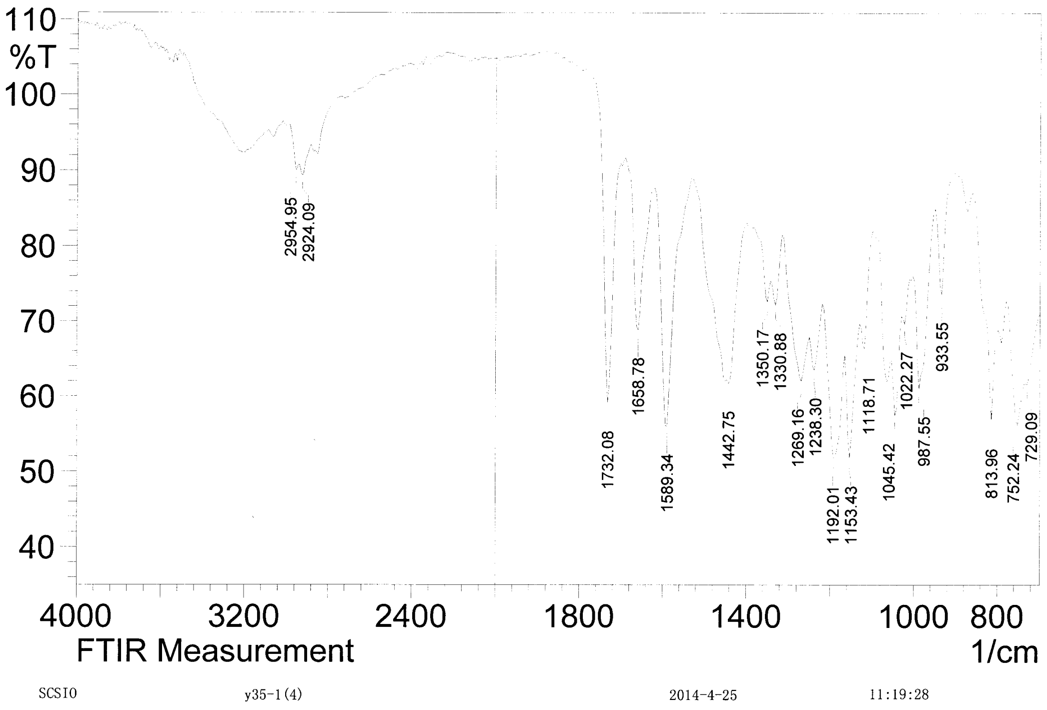

Figure S52. ^{1}H NMR spectrum of **9**.

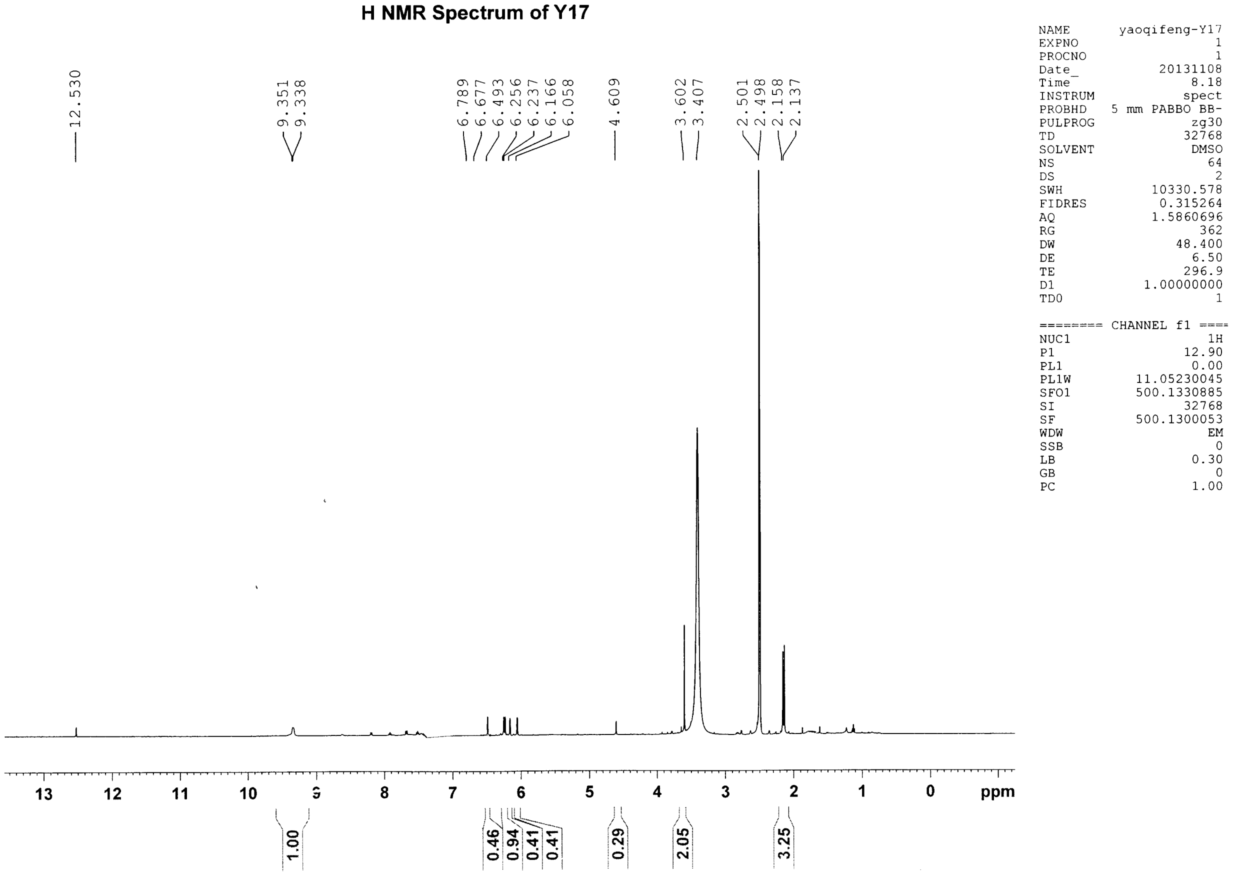

Figure S53. ^{13}C NMR spectrum of **9**.

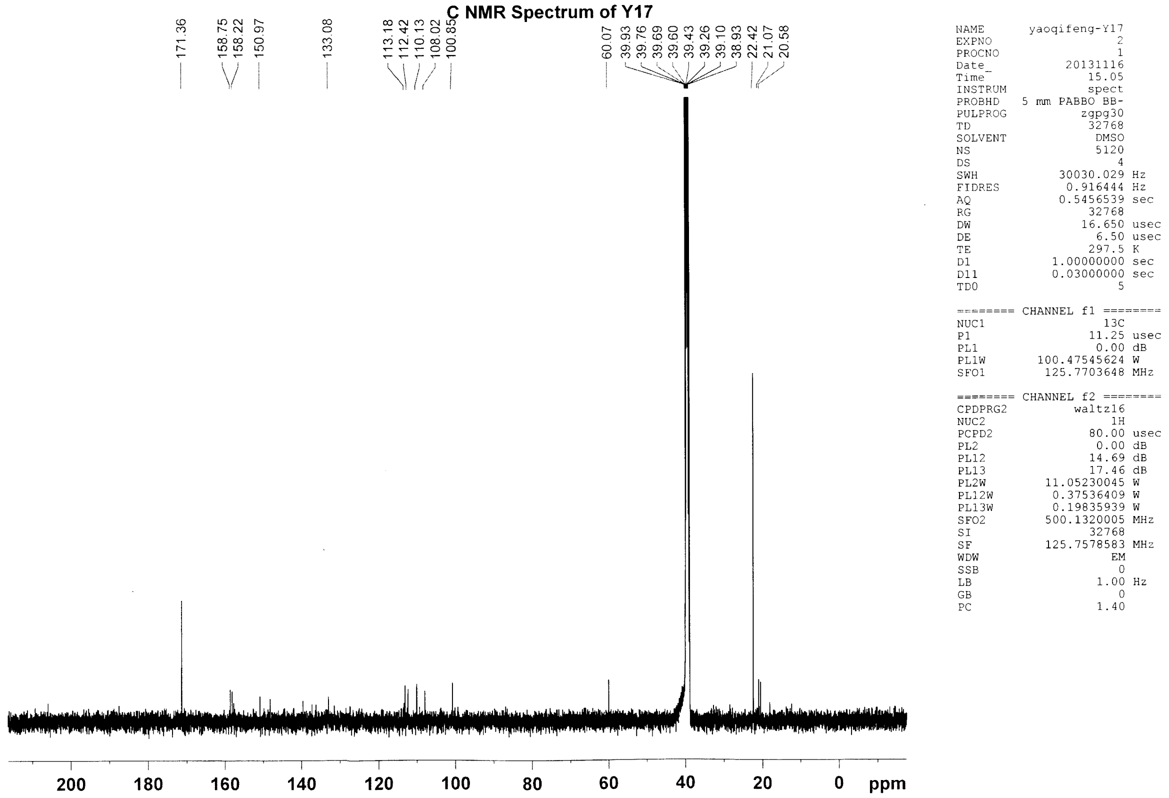

Figure S54. HMBC spectrum of **9**.

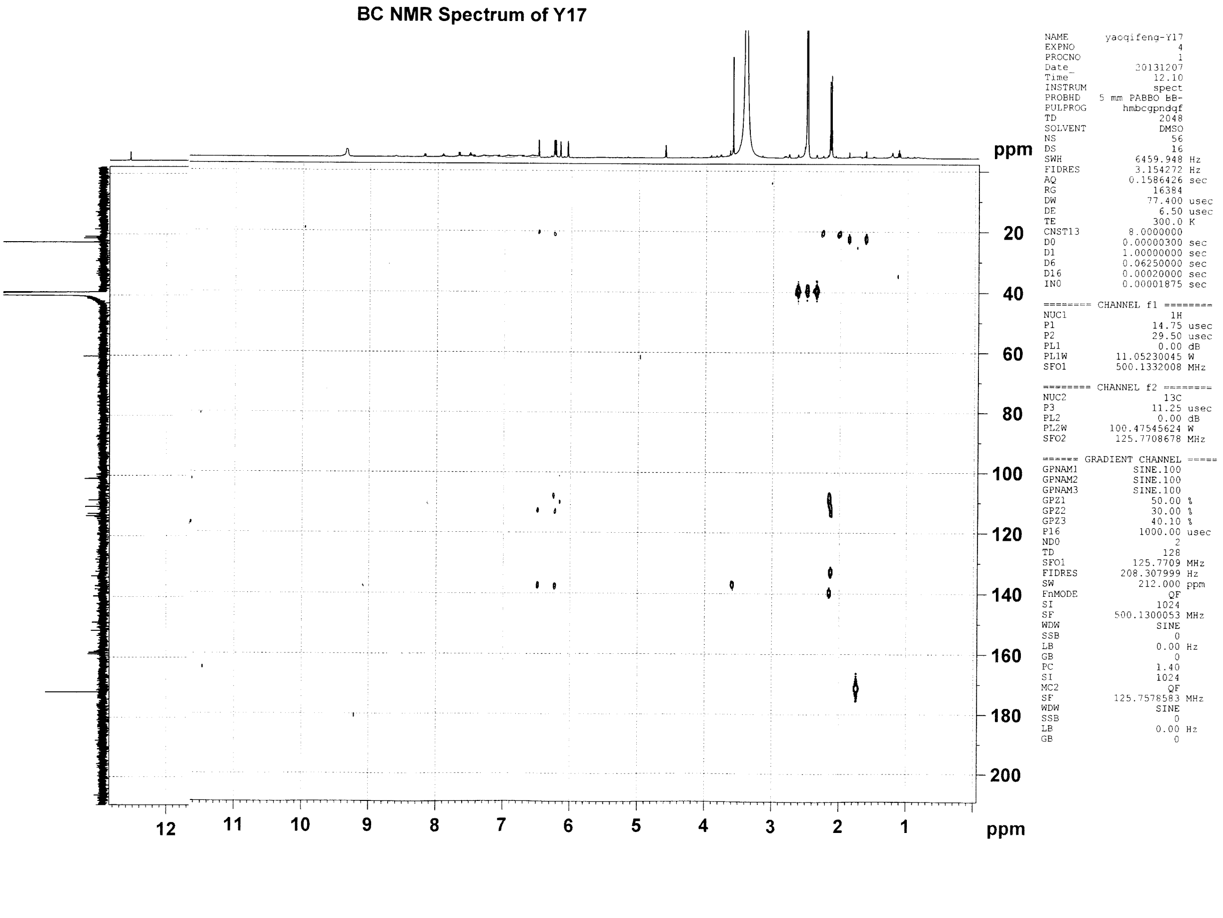

Figure S55. HRESIMS spectrum of **9**.

Mass Spectrum SmartFormula Report

Analysis Info

Analysis Name	D:\Data\MS\data\201404\yaoqifeng-Y17_pos.d	Acquisition Date	4/23/2014 3:03:15 PM
Method	POS_100-2000_Dirrect Infusion.m	Operator	SCSIO
Sample Name	SCSIO	Instrument / Ser#	maXis 29
Comment			

Acquisition Parameter

Source Type	ESI	Ion Polarity	Positive	Set Nebulizer	0.4 Bar
Focus	Not active	Set Capillary	4500 V	Set Dry Heater	180 °C
Scan Begin	100 m/z	Set End Plate Offset	-500 V	Set Dry Gas	4.0 l/min
Scan End	2000 m/z	Set Collision Cell RF	2000.0 Vpp	Set Divert Valve	Waste

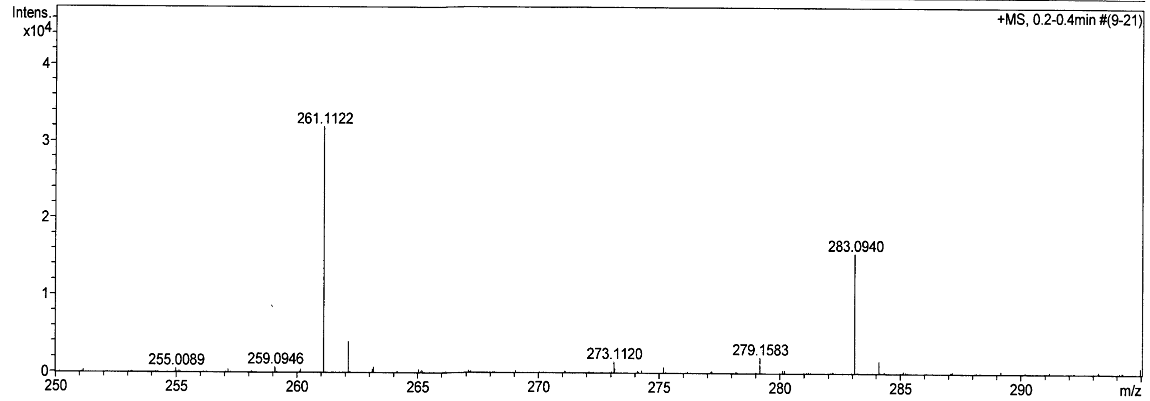

Meas. m/z	#	Formula	Score	m/z	err [mDa]	err [ppm]	mSigma	rdb	e⁻ Conf	N-Rule
261.1122	1	C 15 H 17 O 4	100.00	261.1121	-0.0	-0.1	23.0	7.5	even	ok
283.0940	1	C 15 H 16 Na O 4	100.00	283.0941	0.1	0.4	35.5	7.5	even	ok

Figure S56. IR spectrum of **9**.

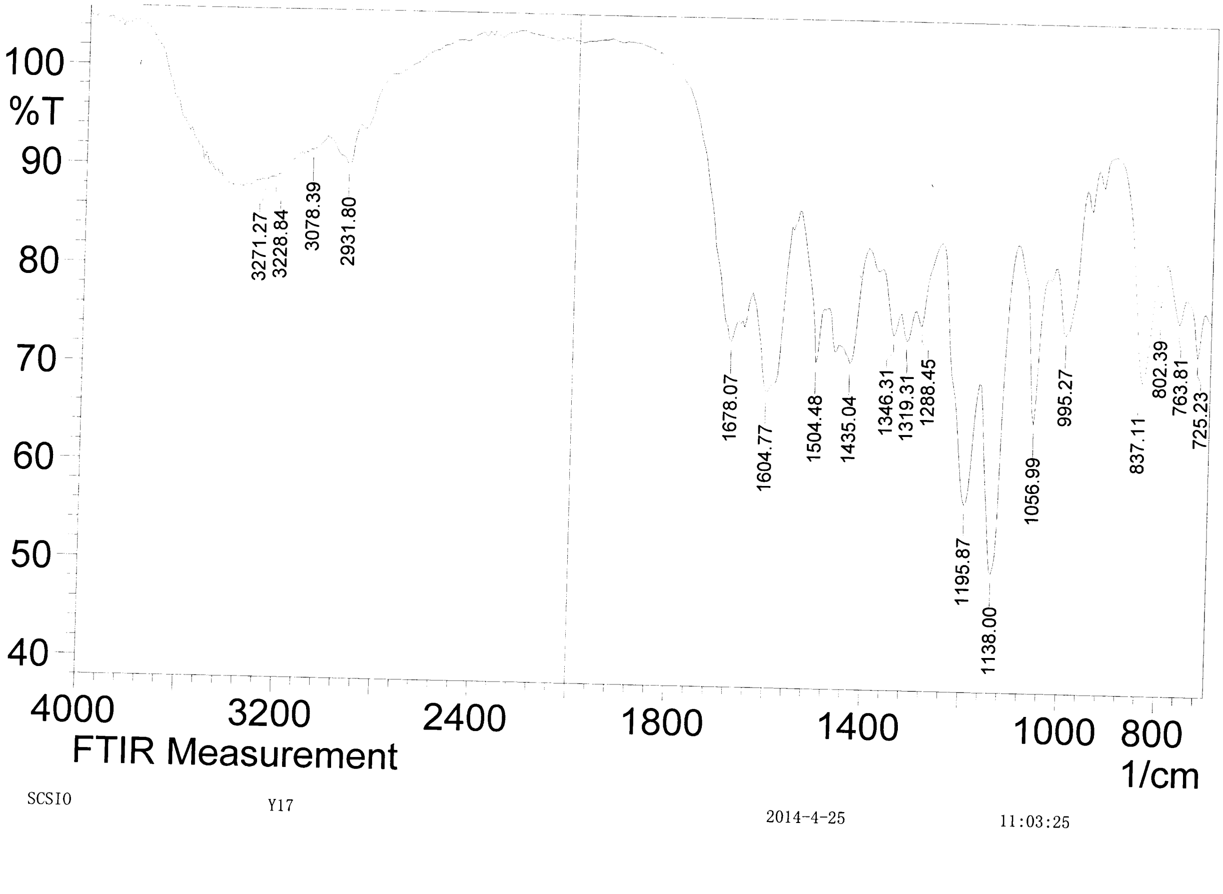

SCSIO Y17 2014-4-25 11:03:25

Figure S57. ^{1}H NMR spectrum of **10**.

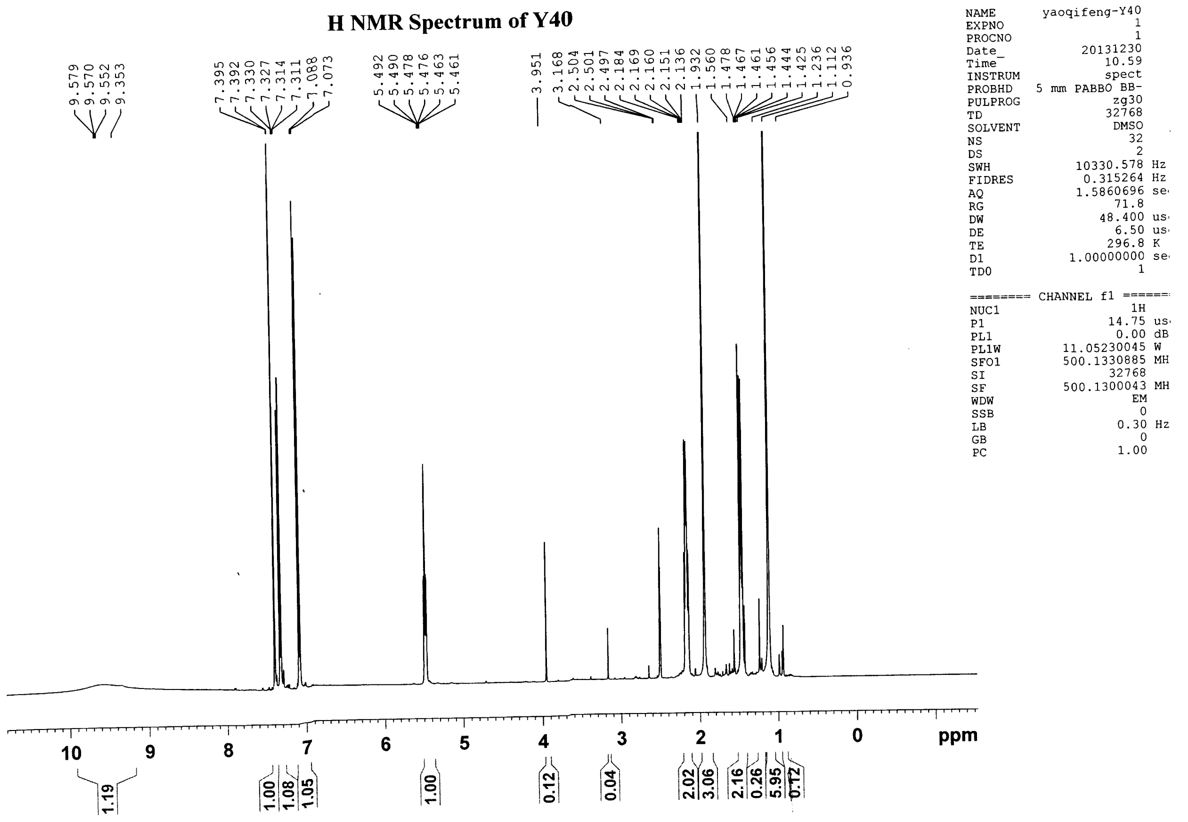

Figure S58. ^{13}C NMR spectrum of **10**.

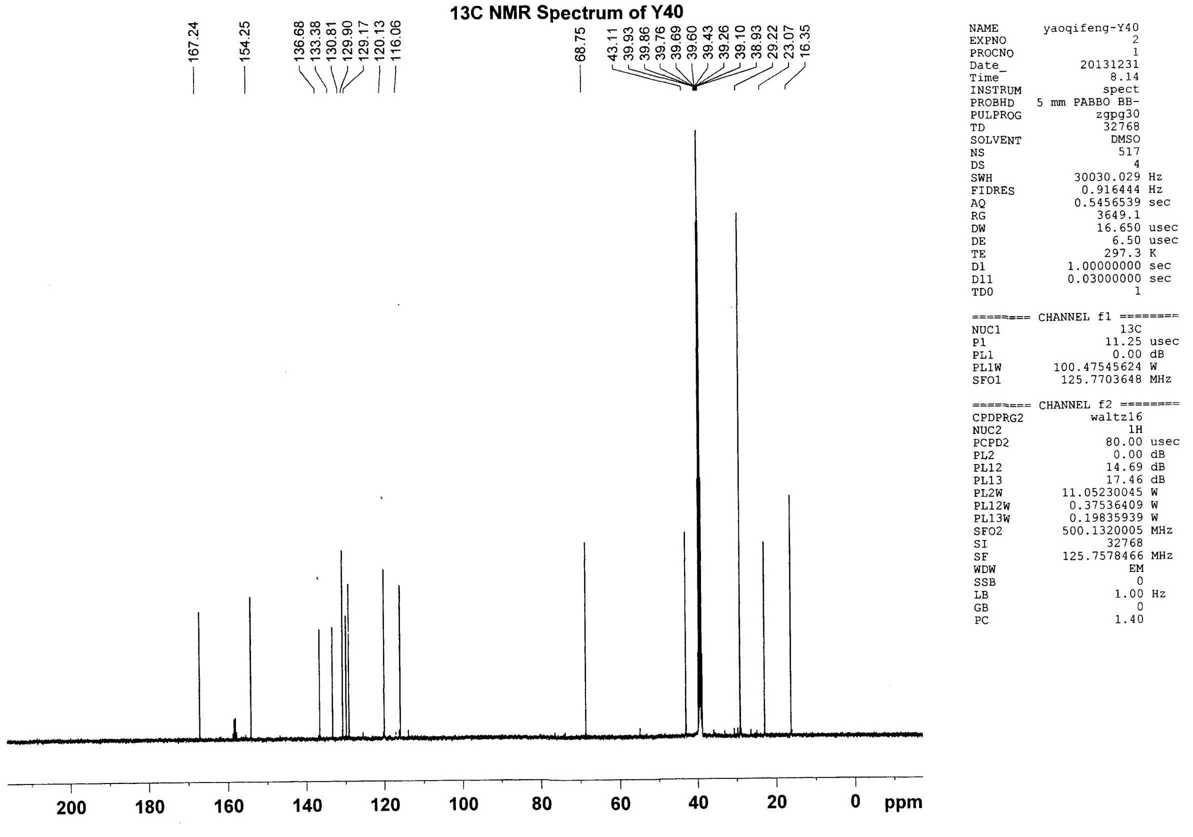

Figure S59. HMBC spectrum of **10**.

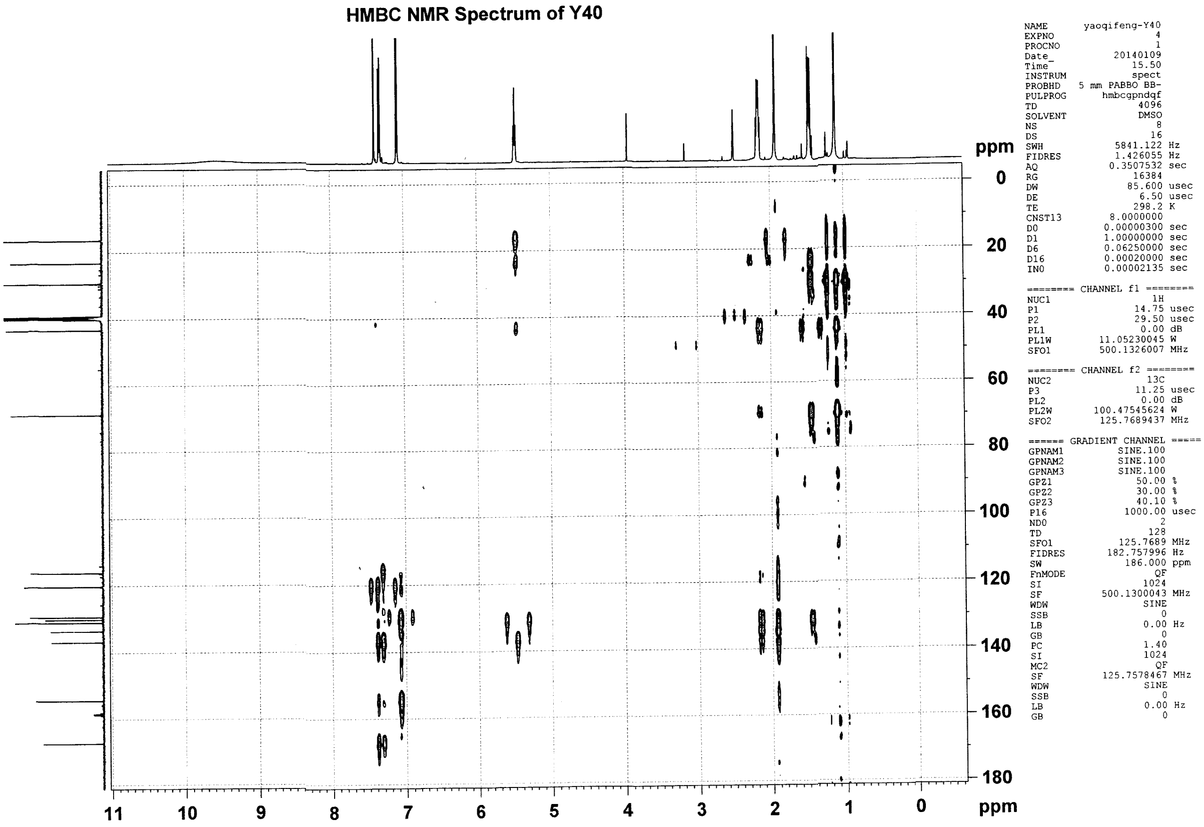

Figure S60. H-H COSY spectrum of **10**.

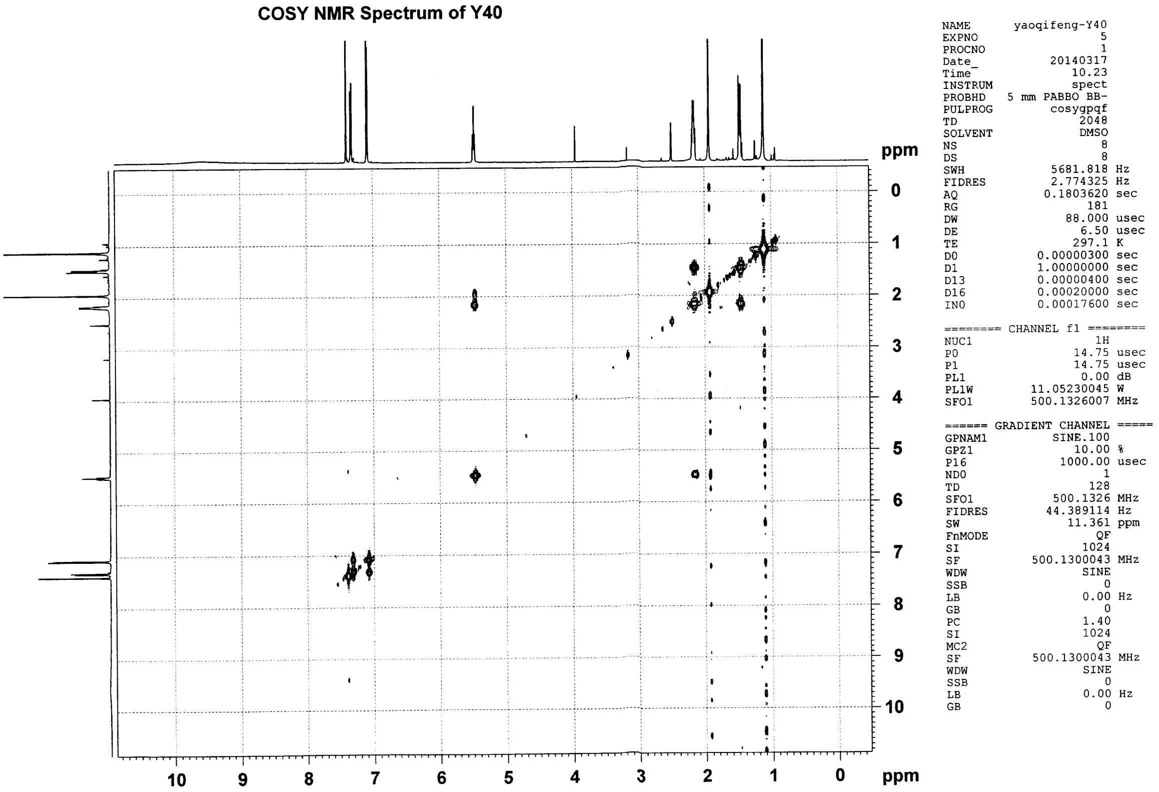

Figure S61. HRESIMS spectrum of **10**.

Mass Spectrum SmartFormula Report

**Analysis Info**		Acquisition Date	4/23/2014 3:19:38 PM
Analysis Name	D:\Data\MS\data\201404\yaoqifeng-Y40_pos.d		
Method	POS_100-2000_Dirrect Infusion.m	Operator	SCSIO
Sample Name	SCSIO	Instrument / Ser#	maXis 29
Comment			

Acquisition Parameter

Source Type	ESI	Ion Polarity	Positive	Set Nebulizer	0.4 Bar		
Focus	Not active	Set Capillary	4500 V	Set Dry Heater	180 °C		
Scan Begin	100 m/z	Set End Plate Offset	-500 V	Set Dry Gas	4.0 l/min		
Scan End	2000 m/z	Set Collision Cell RF	2000.0 Vpp	Set Divert Valve	Waste		

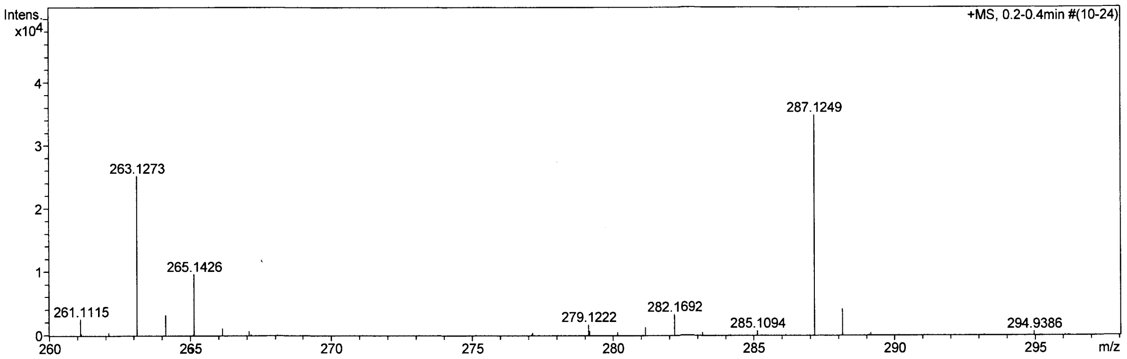

Meas. m/z	#	Formula	Score	m/z	err [mDa]	err [ppm]	mSigma	rdb	e⁻ Conf	N-Rule
265.1426	1	C 15 H 21 O 4	100.00	265.1434	0.9	3.3	25.2	5.5	even	ok
287.1249	1	C 15 H 20 Na O 4	100.00	287.1254	0.5	1.7	26.4	5.5	even	ok

Figure S62. IR spectrum of **10**.

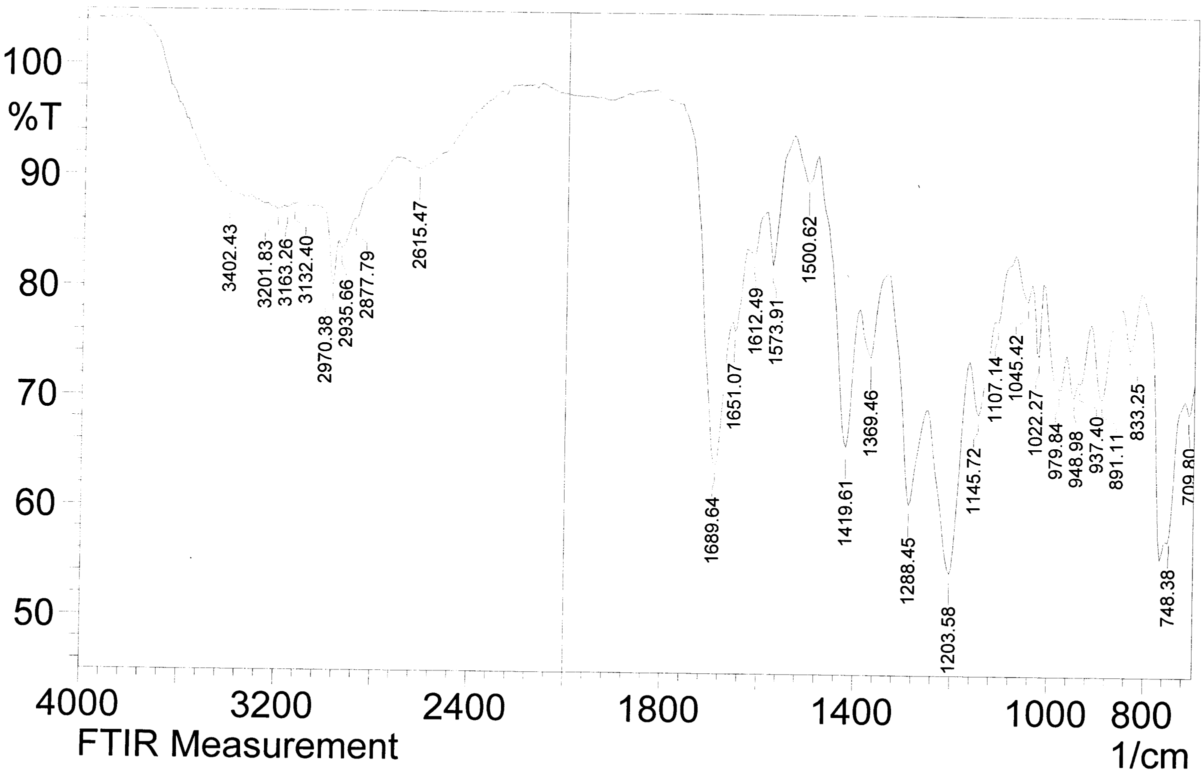

SCSIO Y40 2014-4-25 10:40:13

Figure S63. ^{1}H NMR spectrum of **11**.

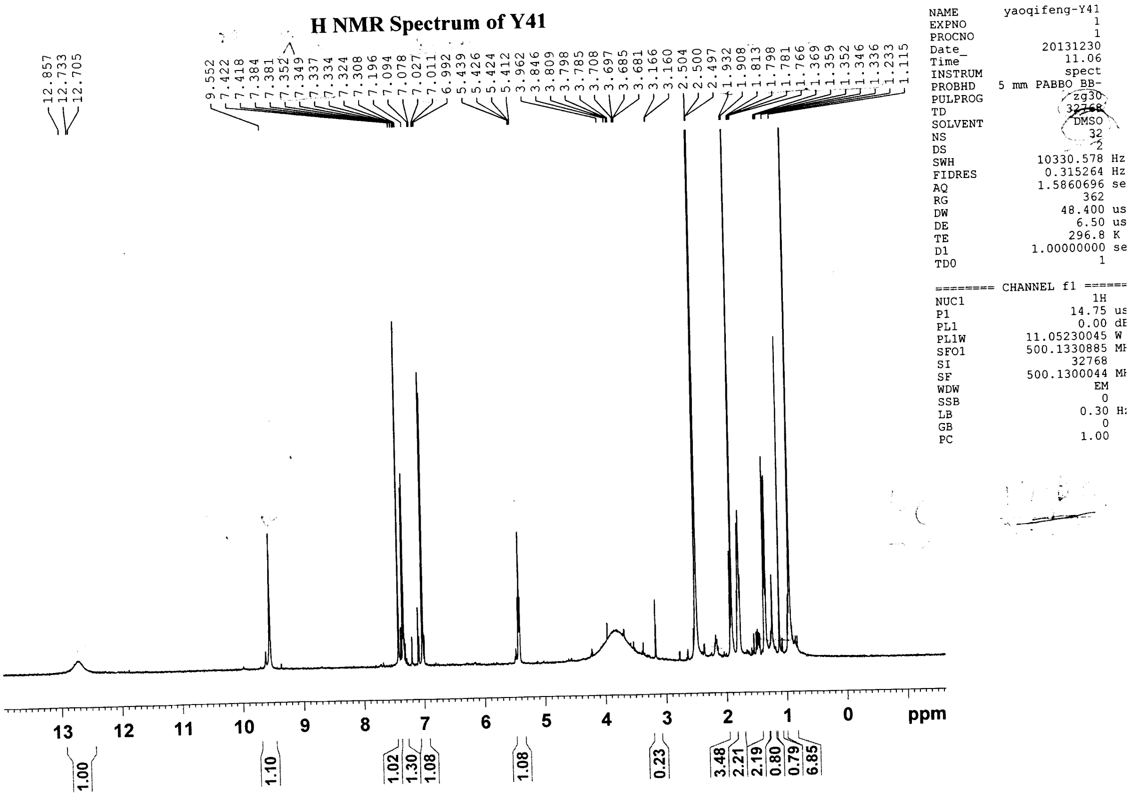

Figure S64. ^{13}C NMR spectrum of **11**.

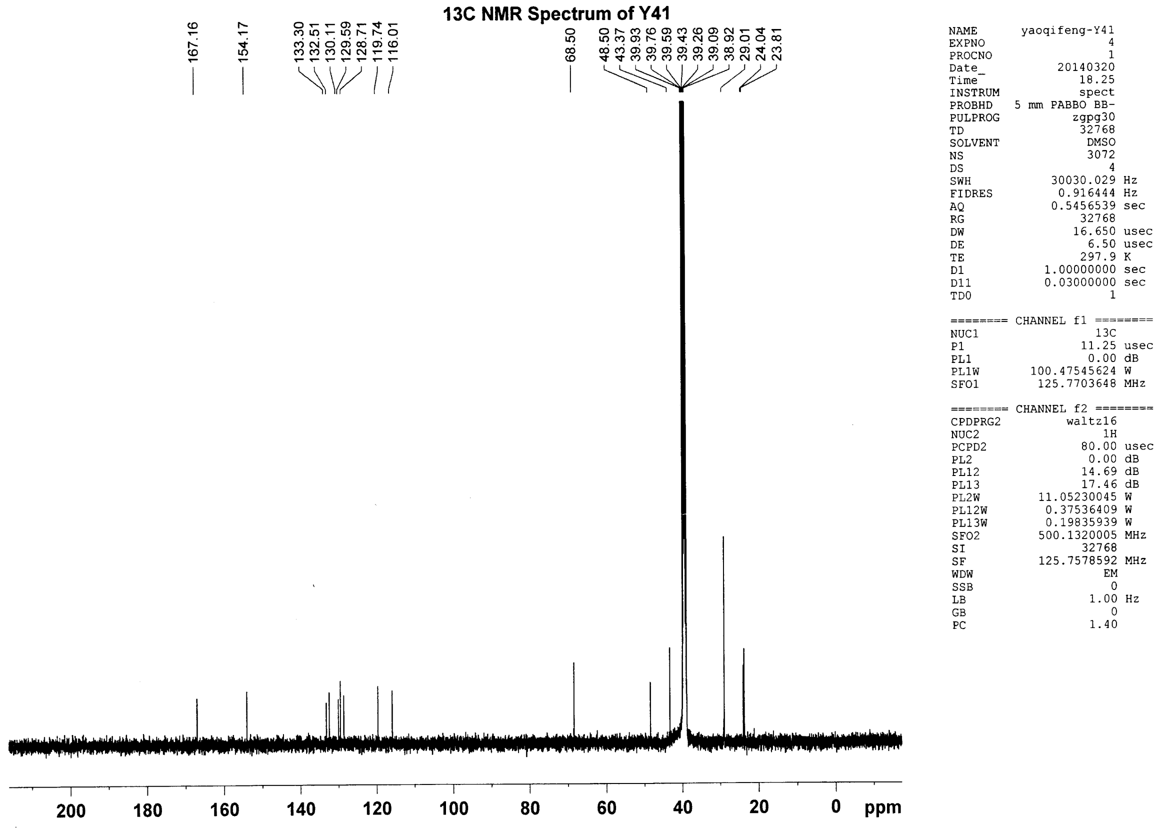

Figure S65. HMBC spectrum of **11**.

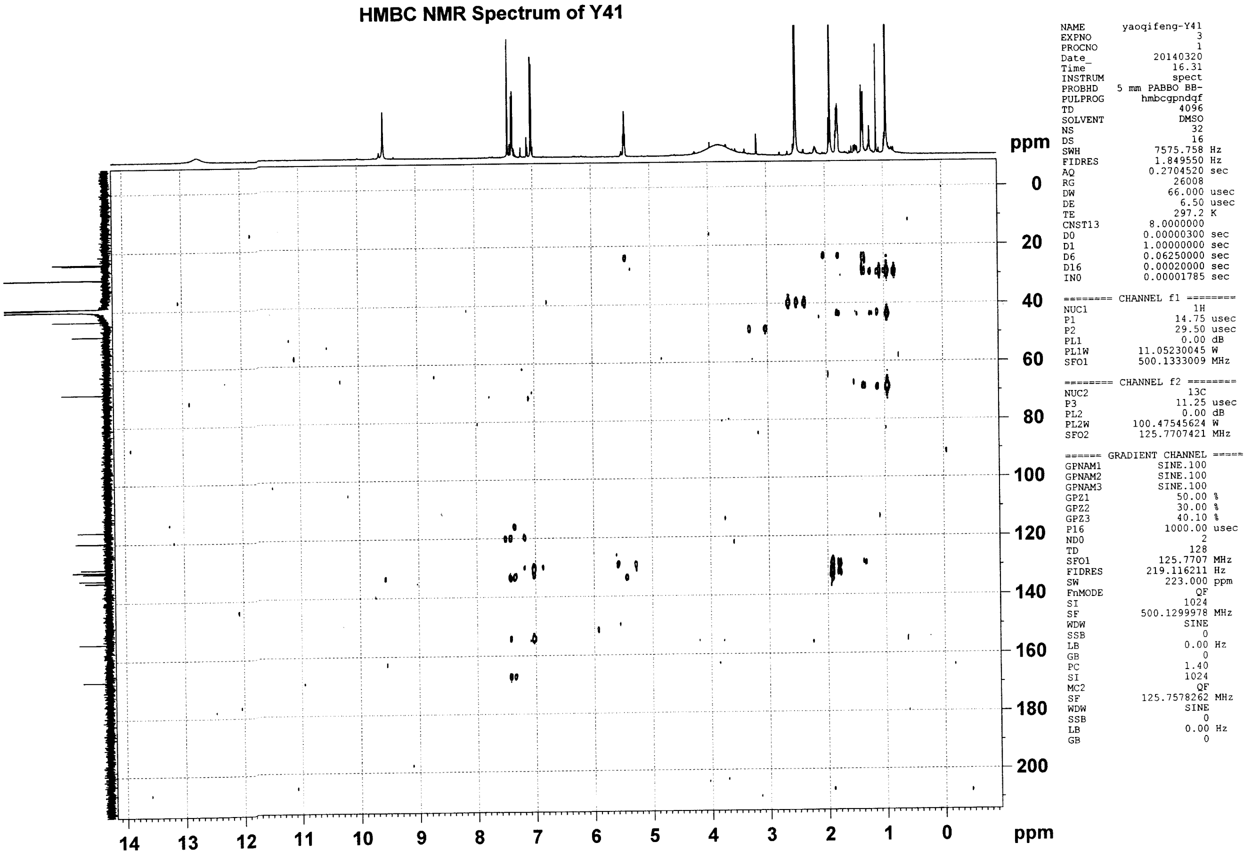

Figure S66. HRESIMS spectrum of **11**.

Mass Spectrum SmartFormula Report

**Analysis Info**		Acquisition Date	4/23/2014 3:29:20 PM
Analysis Name	D:\Data\MS\data\201404\yaoqifeng-Y41-1_pos.d		
Method	POS_100-2000_Dirrect Infusion.m	Operator	SCSIO
Sample Name	SCSIO	Instrument / Ser#	maXis 29
Comment			

Acquisition Parameter

Source Type	ESI	Ion Polarity	Positive	Set Nebulizer	0.4 Bar
Focus	Not active	Set Capillary	4500 V	Set Dry Heater	180 °C
Scan Begin	100 m/z	Set End Plate Offset	-500 V	Set Dry Gas	4.0 l/min
Scan End	2000 m/z	Set Collision Cell RF	2000.0 Vpp	Set Divert Valve	Waste

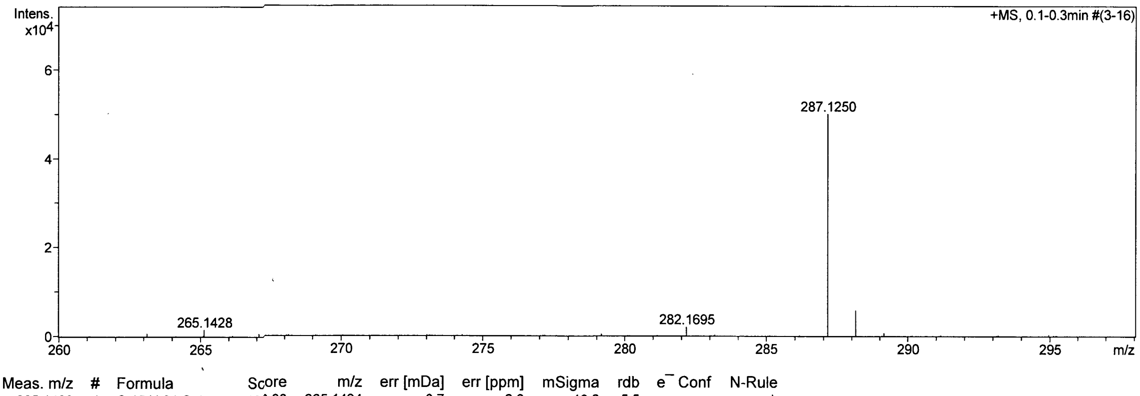

Meas. m/z	#	Formula	Score	m/z	err [mDa]	err [ppm]	mSigma	rdb	e⁻ Conf	N-Rule
265.1428	1	C 15 H 21 O 4	100.00	265.1434	0.7	2.6	18.2	5.5	even	ok
287.1250	1	C 15 H 20 Na O 4	100.00	287.1254	0.3	1.2	28.4	5.5	even	ok

Figure S67. IR spectrum of **11**.

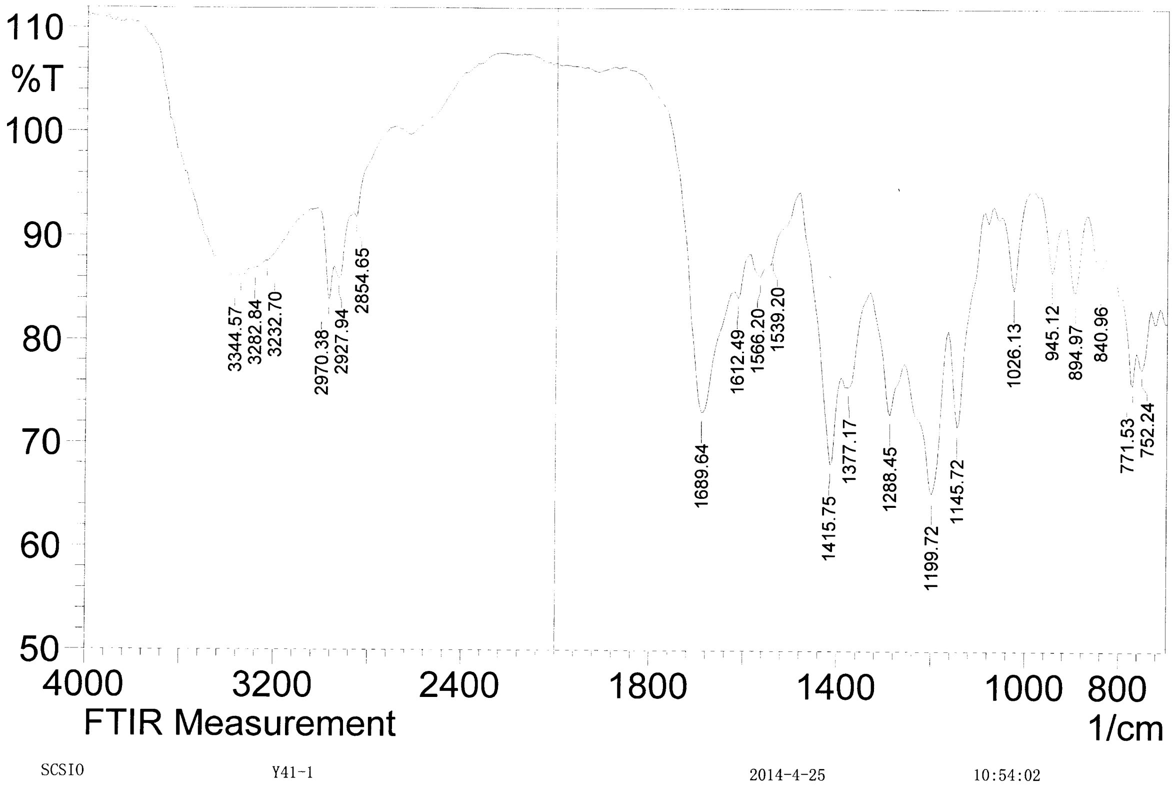

Figure S68. HPLC analysis of compound **5** on a chiral column OD-H (Daicel corporation, Osaka, Japan) (IPA/ n-hexane 30:70) and a C18 column (impurity substance was drought in after several transfer). (**a**) HPLC analysis of compound **5** on a chiral column OD-H; (**b**) HPLC analysis of compound **5** on a C18 column.

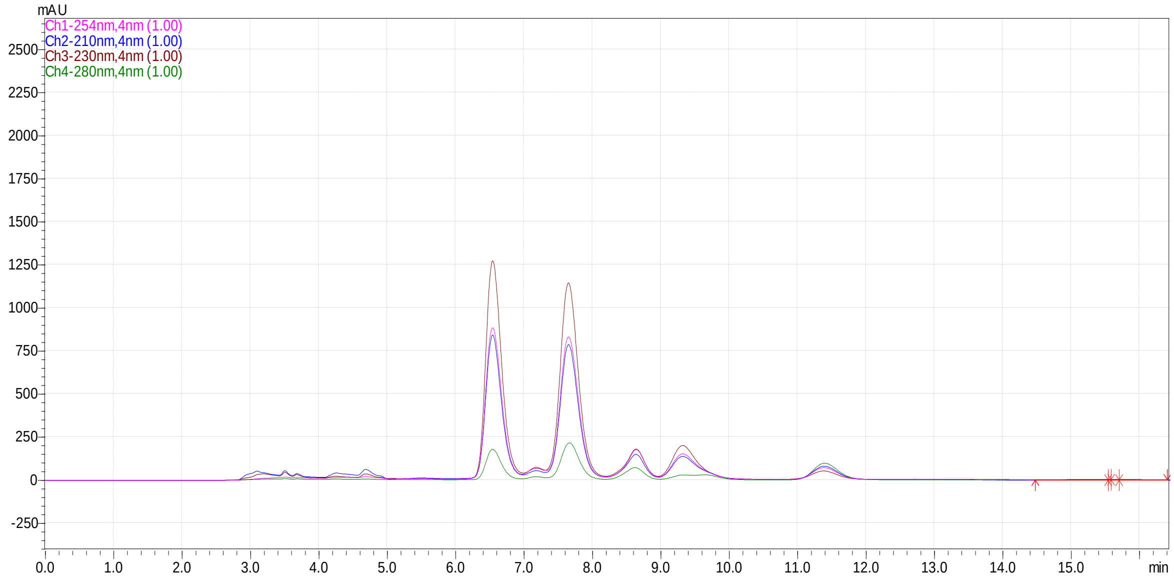

(**a**)

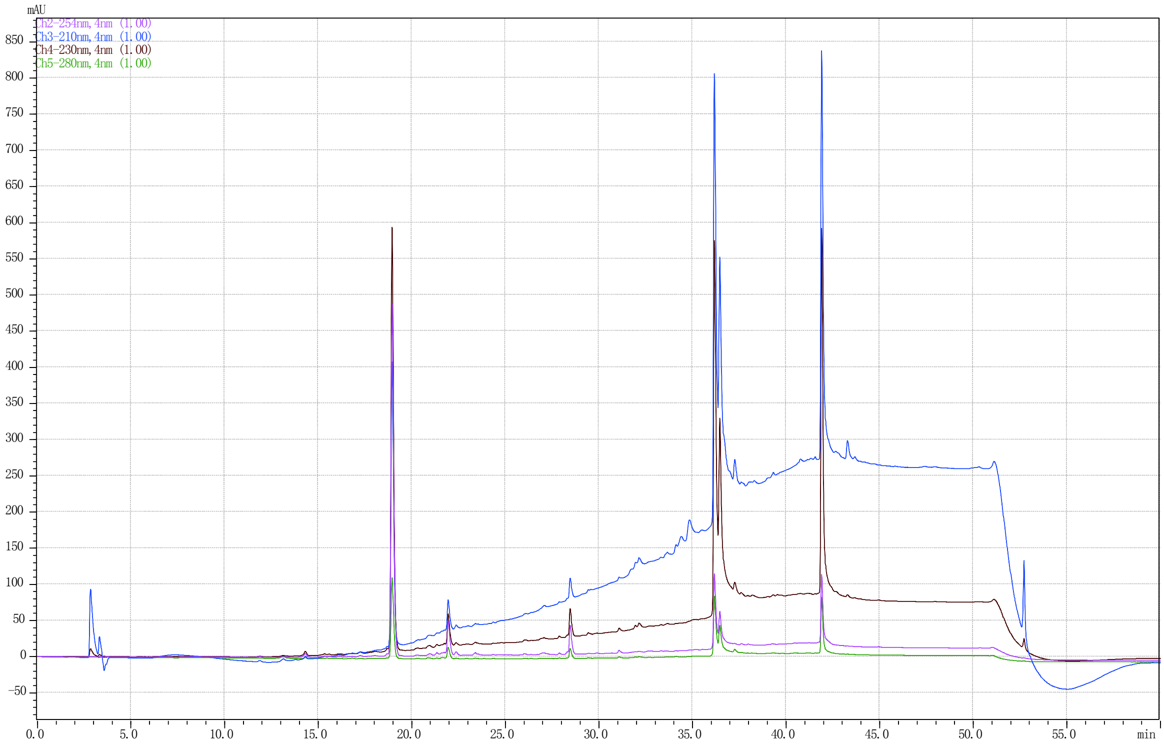

(**b**)

Figure S69. HPLC analysis of compound **6** on a chiral column (IPA/ n-hexane 30:70) and a C18 column. (**a**) HPLC analysis of compound **6** on a chiral column OD-H; (**b**) HPLC analysis of compound **6** on a C18 column.

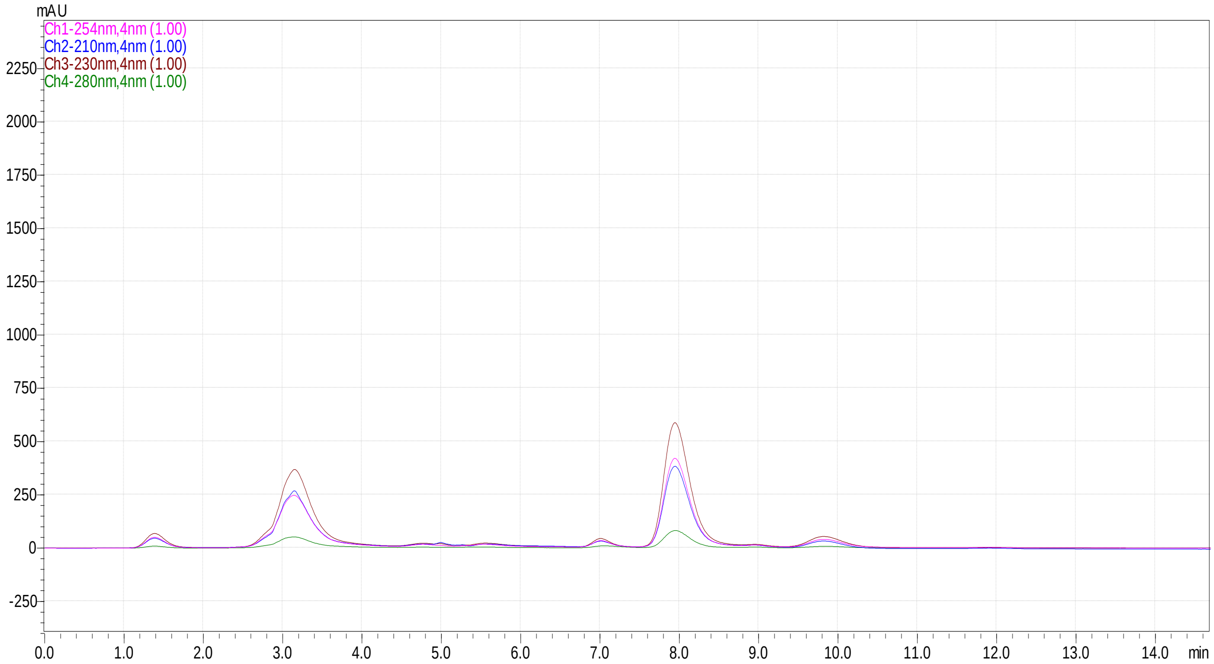

(**a**)

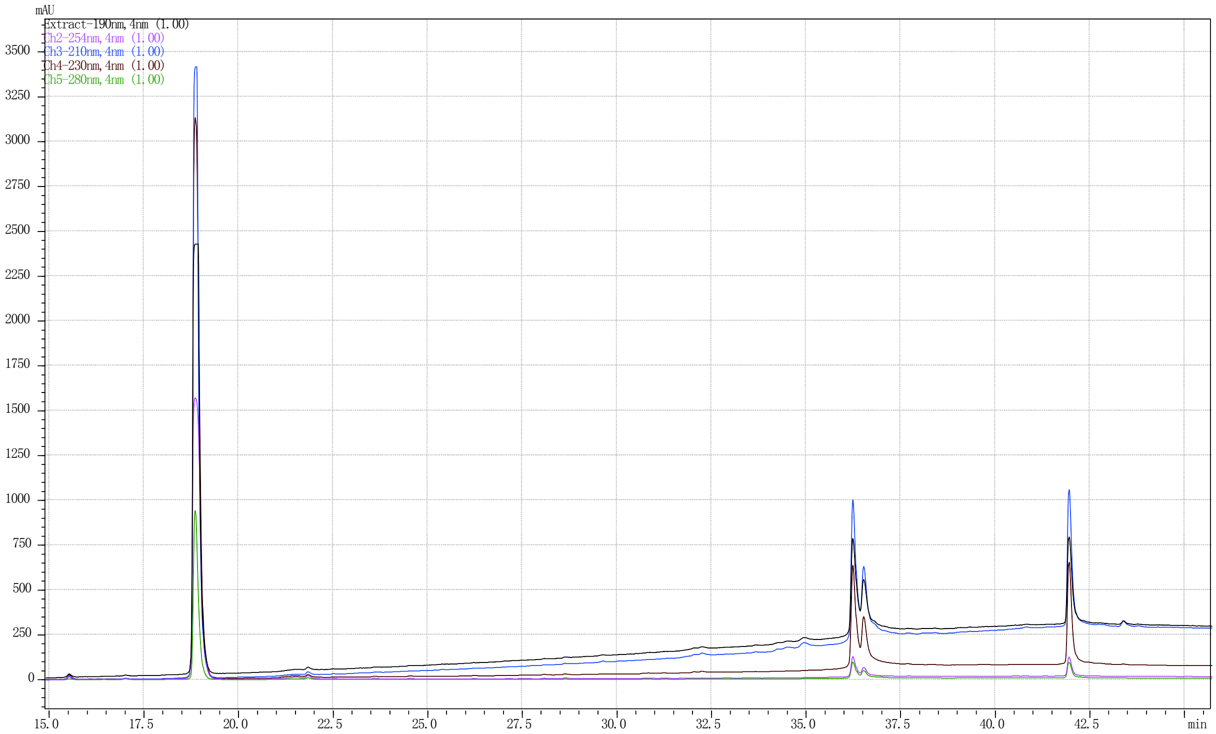

(**b**)

Figure S70. HPLC analysis of compound **7** on a chiral column (IPA/ n-hexane 22:78) and a C18 column (impurity substance was drought in after several transfer). (**a**) HPLC analysis of compound **7** on a chiral column OD-H; (**b**) HPLC analysis of compound **7** on a C18 column.

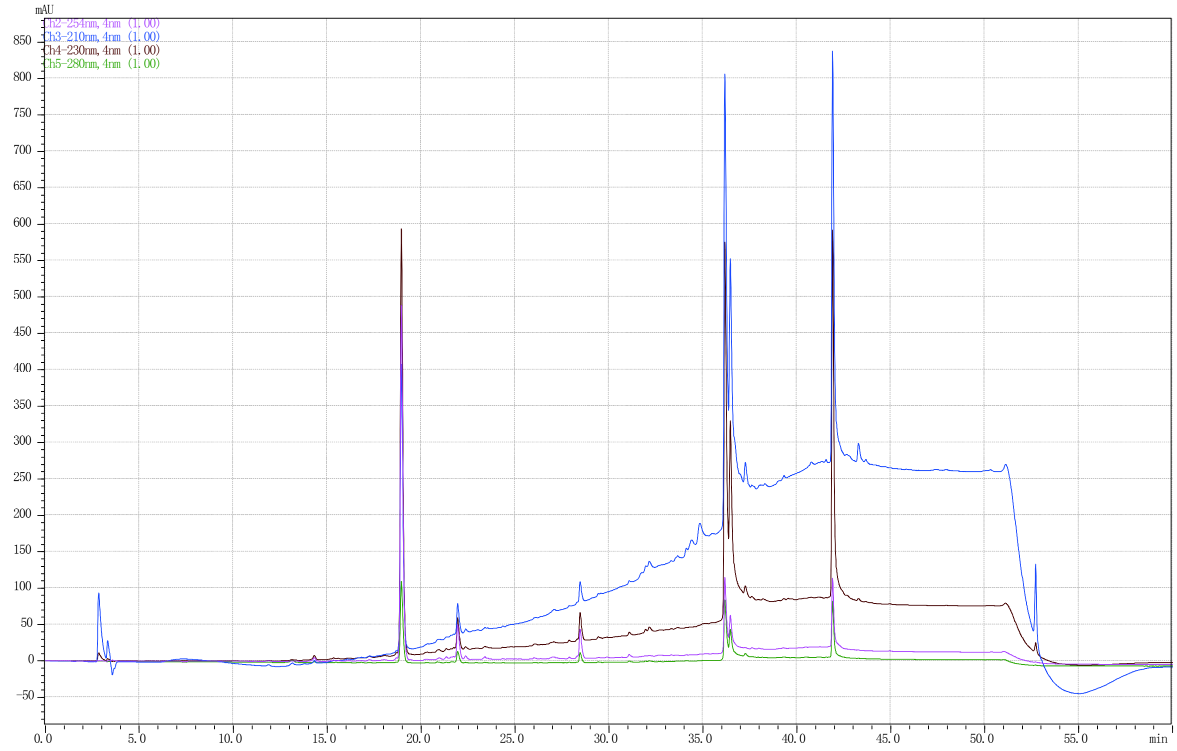

(**a**)

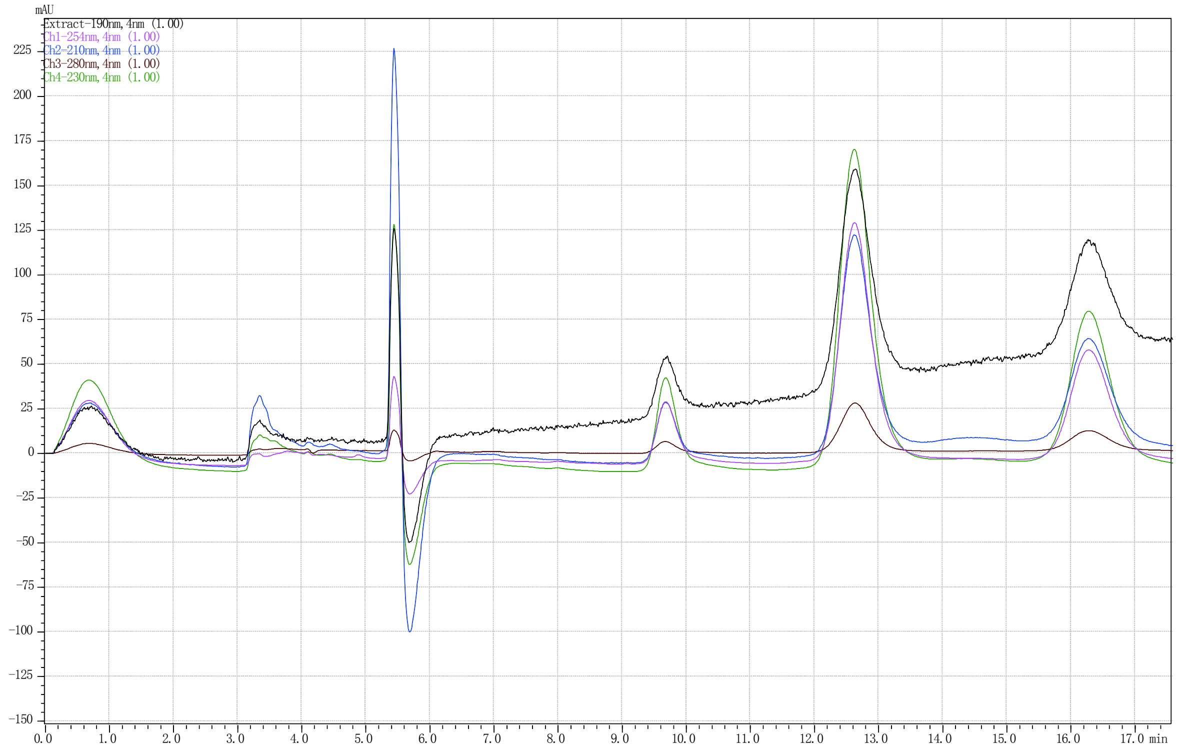

(**b**)

Figure S71. Macro- and micro-morphological observations of strain DFFSCS021.

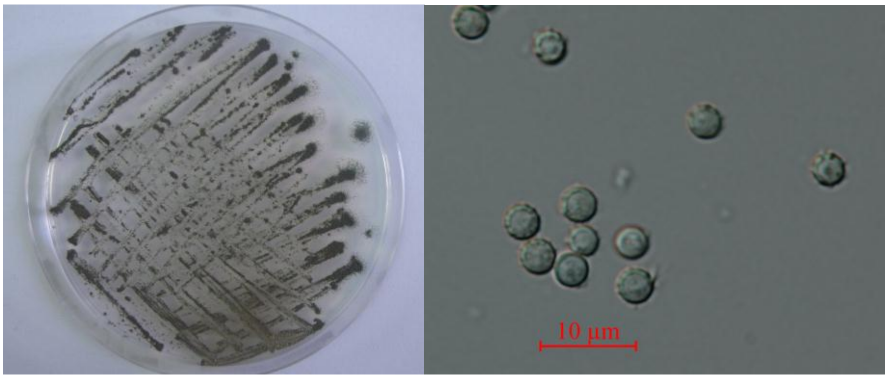

© 2014 by the authors; licensee MDPI, Basel, Switzerland. This article is an open access article distributed under the terms and conditions of the Creative Commons Attribution license (http://creativecommons.org/licenses/by/4.0/).
